# Comparative Effectiveness of Ascorbic Acid vs. Calcium Ascorbate Ingestion on Pharmacokinetic Profiles and Immune Biomarkers in Healthy Adults: A Preliminary Study

**DOI:** 10.3390/nu16193358

**Published:** 2024-10-02

**Authors:** Broderick Dickerson, Drew E. Gonzalez, Ryan Sowinski, Dante Xing, Megan Leonard, Jacob Kendra, Victoria Jenkins, Siddharth Gopalakrishnan, Choongsung Yoo, Joungbo Ko, Syamkumar Sivasankara Pillai, Jigna R. Bhamore, Bhimanagouda S. Patil, Gus A. Wright, Christopher J. Rasmussen, Richard B. Kreider

**Affiliations:** 1 Exercise & Sport Nutrition Lab, Department of Kinesiology and Sport Management, Texas A&M University, College Station, TX 77843, USA; dickersobl5@tamu.edu (B.D.); dg18@tamu.edu (D.E.G.); rjs370@tamu.edu (R.S.); dantexing@tamu.edu (D.X.); meganleonard10@tamu.edu (M.L.); jkendra@tamu.edu (J.K.); victoria.jenkins@tamu.edu (V.J.); sidgopal29@tamu.edu (S.G.); choongsungyoo@tamu.edu (C.Y.); joungboko10@tamu.edu (J.K.); crasmussen@tamu.edu (C.J.R.); 2 Vegetable and Fruit Improvement Center, Department of Horticulture, Texas A&M University, College Station, TX 77843, USA; syam.pillai@skpt.com (S.S.P.); jignabhamore@gmail.com (J.R.B.); bhimanagouda.patil@ag.tamu.edu (B.S.P.); 3 Flow Cytometry Facility, Department of Veterinary Pathobiology, Texas A&M University, College Station, TX 77843, USA; wrightga@tamu.edu

**Keywords:** bioavailability, nutrient absorption, vitamin supplementation

## Abstract

Background: Previous trials have displayed augmented intracellular vitamin C concentrations in the leukocytes at 24 h after acute supplementation with 1000 mg calcium ascorbate (CA), compared to ascorbic acid (AA). Objective: The primary objective was to evaluate comparative leukocyte vitamin C accumulation kinetics over 32 h following acute 250 mg or 500 mg doses from the two sources. Secondary objectives were to evaluate neutrophil phagocytic function and lymphocyte differentiation between the two sources of vitamin C. Methods: Ninety-three healthy females (250 mg, *n* = 27; 500 mg, *n* = 24) and males (250 mg, *n* = 19; 500 mg, *n* = 23) were assigned to ingest a single dose of CA or AA providing 250 mg or 500 mg of vitamin C in two separate double-blind, randomized crossover trials. Results: There were no significant differences in the primary or secondary outcomes between the two treatments in the 250 mg low-dose study. Conversely, there was evidence that ingestion of 500 mg of CA increased DHA in plasma, increased neutrophil functionality during the first 8 h of the PK study, promoted increased natural killer cells, and altered weight-adjusted PK profiles, suggesting greater volume distribution and clearance from the blood. Conclusions: These findings indicate that 500 mg of CA may promote some immune benefits compared to 500 mg of AA ingestion.

## 1. Introduction

Vitamin C is nutritionally essential for collagen, glutathione, and L-carnitine synthesis and metabolism. Various forms of vitamin C are used in supplement formulations (i.e., calcium ascorbate (CA), liposomal encapsulated ascorbic acid (AA), liposomal encapsulated lipid metabolite AA, slow-release AA, lipid metabolite AA) with companies making claims these forms are superior to AA in absorptive ability, bioavailability, retention in leukocytes, and tolerability. Most comparative trials assess the efficacy of CA against AA [1,2,3,4,5,6,7,8]; however, several trials have shown higher retention, greater absorption, and tolerance in liposomal encapsulated vitamin C [8,9,10,11,12,13], but equivocal findings with sustained release vitamin C in plasma vitamin C concentration compared to AA [14,15,16].

Acute doses at 1000 mg of calcium ascorbate (CA), compared to AA, have been shown to enhance vitamin C retention in leukocytes as long as 24 h following ingestion [1,2]. In addition, Van and colleagues [5] found reduced frequency and severity of cold-like symptoms in those supplementing 1000 mg CA for 60 days, which could be attributed to leukocyte function. Neutrophils are a heavily abundant leukocyte type. It is believed that the presence of vitamin C in neutrophils protects them from oxidative burst [16]. Studies have found neutrophil function can be impaired when vitamin C is depleted. Vitamin C supplementation, alone or coupled with other micronutrients, can augment neutrophil functions [16,17,18]. Therefore, increased vitamin C retention in leukocytes and neutrophils may augment functional responses.

Studies incorporating lower doses (250–500 mg) are scant, with most trials testing the effects of higher doses (≥1000 mg) on plasma vitamin C status and immune function. The primary purpose of these trials was to evaluate comparative leukocyte and plasma vitamin C accumulation kinetics over 32 h following acute doses of 250 mg and 500 mg of AA and CA. Secondary objectives were to evaluate comparative neutrophil phagocytic function, lymphocyte differentiation, and natural killer cells in lymphocytes between the two sources. We hypothesized that acute supplementation with CA will elicit superior plasma and leukocyte pharmacokinetics and 24 h neutrophil phagocytic function in comparison to equimolar doses of vitamin C from AA. Subsequently, a more detailed analysis of leukocyte cell differentiation markers (CD3, CD4, CD8, CD38, regulatory T cells) and their expression of inflammatory markers (IFNg, TNFa) and cytokines (IL2, IL6) and natural killer cells (NK) on PBMC was performed on 0 and 24 h following exposure to a mitogen. This provides additional insight into whether these forms of vitamin C differentially affect the lymphocyte response to a standard immune challenge. The following describes the methods and results observed.

## 2. Methods

### 2.1. Experimental Design

Researchers adopted a randomized, counterbalanced, double-blind, crossover approach for this study. Approval was given by the University Human Research Protection Program Institutional Review Board (IRB2021-0020F) for the study, which followed moral principles regarding the conduct of human research as designated in the Declaration of Helsinki. This trial was also registered in the International Standard Randomized Control Number Registry (ISRCTN49416083). The independent variable was the type of vitamin C. The co-primary outcomes were a concentration–time curve for plasma and leukocyte ascorbic acid and pharmacokinetic profiles. Secondary outcomes included baseline and 24 h post-supplementation phagocytic activity of neutrophils and leukocyte differentiation markers, inflammatory markers, cytokines, and natural killer cells in PBMC in response to exposure to a mitogen.

### 2.2. Participants

Healthy individuals were enlisted to partake in this pharmacokinetic trial. Participants who met the following inclusion criteria were deemed eligible for the study: (1) Providing written, voluntary, informed consent to partake in the trial; (2) Healthy adults (male and female, aged 18 to 60); (3) Body Mass Index (BMI) of 18–30 kg/m^2^; (4) Non-smokers or have stopped smoking for more than a year; and (5) Willingness to ingest a low-vitamin C diet during the trial. Exclusion criteria included: (1) Being pregnant, breastfeeding, or having a desire for pregnancy during the study; (2) Use of over the counter or prescribed drugs/medications known to affect vitamin C metabolism within 72 h of trial onset and during the trial, such as non-steroidal anti-inflammatory drugs (NSAIDs), aspirin, antacids with aluminum, and iron; (3) Proton-pump inhibitor (e.g., Omeprazole, Esomeprazole, Lansoprazole) use within the past month; (4) Multivitamin supplementation with vitamin C within seven days of trial onset and during the trial; (5) Type 1 or 2 diabetes diagnosis; (6) Gastroesophageal reflux disease (GERD) within the past three months; and (7) Gastrointestinal disease, malabsorption condition, or any other condition that may adversely affect the participant’s capability to finish the trial or its analytes or pose a major risk to the participant.

**Figure 1:**
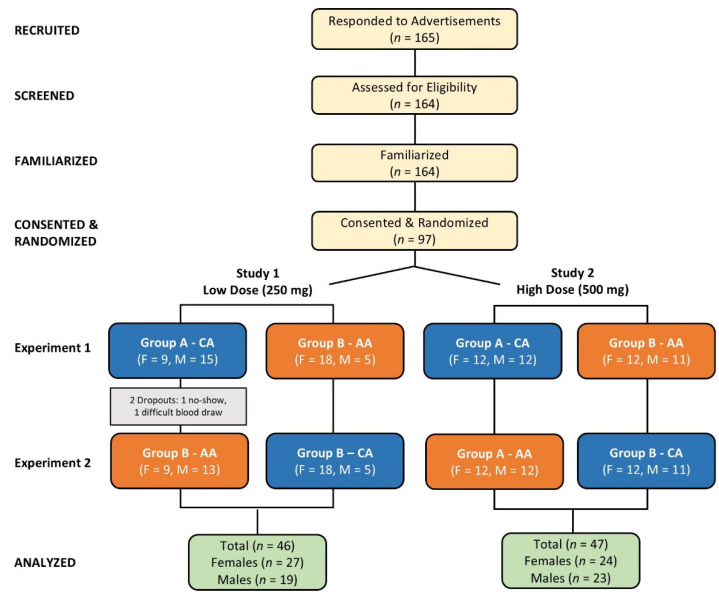
A Consolidated Standards of Reporting Trials (CONSORT) chart for the low- and high-dose studies. CA represents calcium ascorbate; AA represents ascorbic acid.

Figure 1 depicts a Consolidated Standards of Reporting Trials (CONSORT) chart. A total of 165 interested individuals replied to advertisements/announcements and had eligibility examined, of which 164 met preliminary screening eligibility requirements and were familiarized with the study. Ninety-seven individuals consented to participate and were randomized for testing into 250 mg study (low-dose study) or 500 mg study (high-dose study). After the study assignment, participants were randomized and counterbalanced into their respective treatments. Treatment designations are designated by experimental sessions with the quantity of females and males examined (*n*) displayed. One participant was a no-show after completing their first testing session. Another participant discontinued the study due to difficulty obtaining blood samples. Forty-six participants (27 females, 19 males) completed the low-dose study, and 47 (24 females, 23 females) completed the high-dose study. Statistical analyses were performed on each study separately.

### 2.3. Experimental Timeline

**Figure 2:**
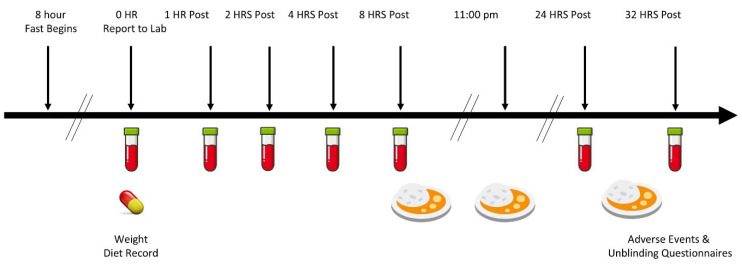
Overview of the experimental trial timeline.

Figure 2 shows the experimental timeline. Participants were recruited by emailing, posting, and/or publishing advertisement leaflets in online and/or local print locations. Those who conveyed interest in study participation were consulted via email or phone to determine general eligibility. Those who met preliminary eligibility criteria were invited to an in-person initial consultation, where they obtained information about the study protocols and provided written informed consent. Individuals meeting inclusion criteria were randomized to participate in either the low-dose or high-dose study and given diet recording instructions as well as a list of foods and beverages containing high levels of vitamin C. Participants were asked to record four-day food and beverage logs before testing so they could duplicate dietary intake before each testing session, as well as to refrain from consuming high vitamin C food/supplements within seven days before starting the study and during the trial. Participants were also asked to fast the night before the test sessions starting at midnight, so they appeared at the clinical laboratory site in the early morning in a fasted state for a venous blood draw. Participants arrived at the lab in a fasted state with their four -day food logs, then obtained a fasting blood sample and ingested the assigned treatment. Venous blood samples were collected after 1, 2, 4, and 8 h. Participants then completed an adverse event questionnaire and an unblinding questionnaire. Participants could read, study, and/or watch movies during the experiment. Low vitamin C-containing standardized meals were administered to participants after the 8th-hour blood sample (about 4:00 pm) was provided and before retiring (about 11:00 pm). Participants then returned to the lab in a fasted state the next morning (about 8:00 am) for a 24 h blood sample. They were then given a standardized low-vitamin C-containing breakfast. They then donated a 32 h post-ingestion blood sample (about 4:00 pm). Participants were instructed not to engage in any structured exercise during this time. A 7–28-day washout period (evaluated at the same time of the menstrual cycle for women) was observed by the participants, who then repeated the experiment with the alternate form.

### 2.4. Supplementation Procedure

The supplement order was counterbalanced with a balanced Latin Square method, and the treatments were administered in a double-blind and crossover manner [19]. Ascorbic acid (AA) and calcium ascorbate (CA) raw materials, along with certificates of analysis and purity, were provided by The Bountiful Co. (Ronkonkoma, NY, USA). The CA contained about 11% calcium by molecular weight. The Bountiful Co. (Ronkonkoma, NY, USA) encapsulated nutrients in opaque white bovine capsules. Microcrystalline cellulose, croscarmellose sodium, and stearic acid powder were used to match volume, taste, and color. Participants were matched based on gender and body mass and randomized into low (250 mg)- or high-dose (500 mg) treatments in a counterbalanced manner. The studies were conducted independently, so once this initial randomization occurred, the participants ingested the remaining low-dose or high-dose treatments.

## 3. Procedures

### 3.1. Descriptives

Height and body mass were measured with a calibrated (±0.02 kg) digital scale (Health-O-Meter Professional 500KL, Pelstar LLC, Alsip, IL, USA). Systolic and diastolic blood pressure (SBP and DBP, respectively) at the brachial artery were measured using a digital, mobile blood pressure device (Connex^®^ ProBP™ 3400, Welch Allyn, Tilburg, NL, USA) obtained in an upright, seated position after a five-minute rest period. Palpation of the radial artery was used to calculate resting heart rate according to standard procedures [20].

### 3.2. Blood Collection

Participants donated a post-absorptive blood sample before treatment consumption. Approximately 40 mL of whole blood was obtained at 0 and 24 h, and 20 mL of whole blood was obtained after 1, 2, 4, 8, and 32 h from either an antecubital, forearm, wrist, or hand vein using standard phlebotomy procedures [21,22]. Blood collection was performed using tubes held at room temperature in the following order: (1) Serum Separator Tube (SST) with clot activator; (2) Lithium-Heparin tubes (Hep); (3) Cell Preparation Tubes (CPT); and (4) Dipotassium ethylenediaminetetraacetic acid tubes (K_2_ EDTA) Vacuette^®^ tubes (Becton, Dickinson and Company, Franklin Lakes, NJ, USA). Cell preparation tubes were used only at 0 and 24 h to conduct neutrophil and PBMC extraction from whole blood. Tubes were wiped with alcohol before the blood draw and inverted 8–10 times after to properly mix.

The SST samples were incubated for 15 min at 18–25 °C (room temperature), while the other samples were centrifuged in a refrigerated Thermo Scientific Heraeus MegaFuge 40R Centrifuge (4 °C) (Thermo Electron North America LLC, West Palm Beach, FL, USA) or held in the refrigerator for processing. The SST, K_2_ EDTA, and Hep samples were centrifuged between 4–20 °C at 3000× *g* for 10 min. The CPT samples were centrifuged at room temperature and 1700× *g* for 30 min. All samples were centrifuged within 2 h of collection. Plasma and buffy coat samples were obtained from Hep and aliquoted using standard pipetting techniques into 2 mL cryovials (VWR, Radnor, PA, USA) with a perchloric acid/diethylenetriaminepentaacetic acid (PCA/DTPA) solution in a 1:1 ratio, to prevent oxidation. Samples were stored at −80 °C until they were transported on dry ice for analysis.

Supplemental File S2 shows the detailed methods of extracting and treating peripheral blood mononuclear cells (PBMCs). Briefly, PBMCs were extracted from the CPT plasma samples, isolated, and mixed with a 20% Dimethyl sulfoxide (DMSO-20%) solution in 2 mL cryovials. The DMSO-20% was made with DMSO and a 20% Fetal Bovine Serum mixture (FBS-20%), while FBS-20% was made by adding FBS to a supplemented Roswell Park Memorial Institute Medium solution (sRPMI, VWR, Radnor, PA, USA), a mixture of RPMI with penicillin/streptomycin, 4-(2-hydroxyethyl)-1-piperazineethanesulfonic acid (HEPES), and L-glutamine. NALGENE™ Cryo 1 °C Freezing Containers (Sigma Aldrich, St. Louis, MO, USA) were utilized to freeze cryovials at −80 °C for up to 24 h, which were then transferred into liquid nitrogen (<−180 °C) for storage. Upon analysis, cells were thawed, stimulated with a leukocyte-activating cocktail with BD GolgiPlug™ (BD Biosciences, Franklin Lakes, NJ, USA), stained with surface and intracellular antibodies, and measured with a flow cytometer within 48 h using the methods described below.

Polymorphonuclear leukocytes (PMNs) were extracted from the CPT samples, isolated, and combined with a PMN resuspension fluid (PNM-RS, VWR, Radnor, PA, USA), which was a combination of a non-essential amino acid solution (MEM-NEAA 1%) and complete RPMI (cRPMI), a mixture of RPMI, penicillin/streptomycin, and 10% FBS. PMNs were then plated, infected, fixated, and collected for analysis as described in Supplemental File S2. PMN samples were stored, protected from light, at the store at 4 °C for up to 3 days until analysis via flow cytometer.

### 3.3. Whole Blood and Serum Analysis

Clinical Pathology Labs, Inc. (CPL) (Austin, TX. CLIA #45D0505003, CAP Accreditation #21525-01) measured serum and whole blood samples for complete blood counts (CBC) with percent differentials using an automated multichannel hematology analyzer.

### 3.4. Vitamin C Analysis

The plasma and buffy coat vitamin C were measured using Agilent 1220 series high-performance liquid chromatography (HPLC) equipped with a G1330B FC/ALS autosampler thermostat to maintain the sample temperature between 6–8 °C throughout the analysis. A total of 10 µL of the plasma/buffy coat samples were injected into an Eclipse Plus C18 (250 mm × 4.6 mm, 5 μm) column (Agilent, Santa Clara, CA, USA) maintained at 25 °C for the separation of ascorbates. Two mobile phases, (A) 0.03 mol L^−1^ phosphoric acid and (B) methanol, were used with a 0.5 mL/min flow rate. A gradient program with 100–0% B (0–8 min), 0–10% B (3 min), 10–90% B (4 min), 90–0% B (1 min), and remaining isocratic for 4 min was employed for the separation of vitamin C. The peak was monitored at 243 nm, both ascorbic acid (ASC) and dehydroascorbic acid (DHA) were measured separately to quantify the total vitamin C content, and the results were expressed as ascorbic acid equivalents in µg/mL of plasma/buffy coat sample.

### 3.5. Pharmacokinetic Analysis

Vitamin C dosage, participant weight, and plasma vitamin C values measured for both experiments were input into a two-term, single-analysis system using the Pharmacokinetic Solutions 2.0 pharmacokinetic (PK) software (Summit Research Services, Montrose, CO, USA). The software calculates single-dose AAQS. Each experiment’s calculated values under the respective treatments were analyzed to determine whether CA differentially affects concentration max (Cmax), time max (Tmax), mean residence time (MRT), the area under the curve (AUC), the area under the moment curve (AUMC), volume distribution area (Vd), distribution, elimination rates, steady-state volume distribution area (Vss), absorption rates, clearance area (CL), disappearance/appearance slope, rate, half-life, and elimination phase.

### 3.6. Neutrophil Phagocytosis and Cell Differentiation Analysis

Neutrophil phagocytosis was quantified as described by Smirnov et al. [21] using a Cytek/Amnis Image Stream X Mark II flow cytometer imager (Seattle, WA, USA) with version 201.1.0.765 software (Seattle, WA, USA). The Cytek/Amnis IDEAS image analysis version 6.3 software (Seattle, WA, USA) was utilized to analyze neutrophil phagocytosis data. The T lymphocyte and NK cell differentiation (CD) panel was determined on a subset of 33 participants in the high-dose study using a Cytek/Amnis Cell Stream flow cytometer (Seattle, WA, USA) with version 1.4.76 software (Seattle, WA, USA). Version 10.8.1 BD FlowJo flow cytometry analysis software (Ashland, OR, USA) was utilized to measure the cell data. We used the T cell and NK staining methods described by Lamoreaux and coworkers [22] while portions of the methods described by Mahnke and colleagues [23] and Swanson et al. [24] were combined into one workflow for T cell and NK analysis.

### 3.7. Statistical Analysis

The low-dose and high-dose studies were analyzed independently. A power analysis was conducted to determine sample size, assuming an anticipated 5% enhancement with 80% power in primary outcome variables. Forty participants were deemed sufficiently powered in this crossover design study. Participant randomization and counterbalancing were conducted with a balanced Latin Square design program to treatments in a crossover manner [19]. Data analysis was conducted with IBM^®^ Version 29 SPSS^®^ statistical analyses software (IBM Corp., Armonk, NY, USA). Multivariate and univariate general linear model (GLM) analyses using repeated measures of treatment (between subjects), and time (within subjects) were employed to examine serum vitamin C levels. Mauchly’s test was utilized to calculate sphericity, while distribution normality was evaluated with skewness and kurtosis statistics. Greenhouse–Geisser and Wilks’ Lambda univariate correction tests were employed to assess Time and Treatment × Time interaction effects to adjust for F-value inflation if the assumption of sphericity was violated. Fisher’s Least Significant difference (LSD) tests and 95% upper and lower confidence intervals (CIs) at pre-planned contrasts of interest were used to assess pairwise comparisons of means and post-hoc tests. Since the Greenhouse–Geisser correction was used to adjust for F-value inflation, additional tests to correct for multiple comparisons were not employed, as recommended [25,26]. Type I error probability (*p*-level) was equal to or less than 0.05. Statistical tendencies toward significance were identified when *p*-values were between 0.05 and 0.10 to reduce the likelihood of type II error. The data are presented as means ± standard deviations (SD) or 95% CI’s. The effect size was examined using partial Eta squared ($\eta_{p}^{2}$) values, where values of 0.01 or below represented a small effect, 0.06 to 0.13 represented a medium effect, and 0.14 and above represented a large effect size [27]. Mean changes from baseline with 95% confidence intervals (CI) were analyzed to evaluate the clinical significance of the findings, where means and 95% CI completely below or above baseline values were considered clinically significant [28,29,30,31,32,33,34]. This statistical analysis approach follows recommended methods for analyzing and transparently reporting research [28,29,31] and assessing the clinical significance of the results [28,29,30,31,32,33,34].

## 4. Results

### 4.1. Descriptive Data

Participant descriptive data are shown in Tables S1a and S2b for the low-dose and high-dose studies, respectively. Participants in the low-dose study were 25.5 ± 10 years old, weighed 65.9 ± 11 kg, 168.0 ± 8 cm tall, with a body mass index (BMI) of 23.2 ± 3.1 kg/m^2^, a resting heart rate of 72.0 ± 10 bpm, a SBP of 112.2 ± 11 mmHg, and a DBP of 72.8 ± 7 mmHg. Sex differences were observed in weight, height, and systolic blood pressure. Participants in the high-dose study were 25.1 ± 9 years old, weighed 70.2 ± 13.5 kg, 170.5 ± 9.4 cm tall, had a BMI of 23.9 ± 3.2 kg/m^2^, a resting heart rate of 69.9 ± 9 bpm, a SBP of 115.3 ± 12 mmHg, and a DBP of 71.6 ± 7 mmHg. Sex differences were noted in all baseline measures except age.

### 4.2. Baseline Serum Health Markers

Tables S2a and S2b present baseline serum health markers for the low- and high-dose studies. This included assessing serum creatinine and estimated glomerular filtration rate (eGFR) as markers of kidney function. One-way ANOVA revealed some sex differences in both studies. However, the values observed were within normal ranges for healthy men and women.

### 4.3. Plasma Vitamin C Concentrations

**Figure 3:**
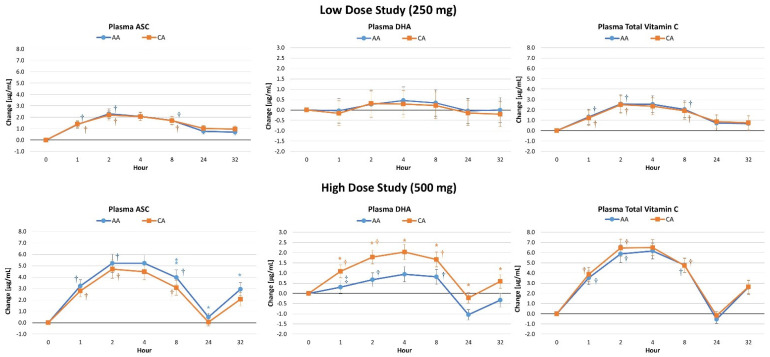
Changes in plasma ascorbic acid (ASC), dehydroascorbic acid (DHA), and total vitamin C levels for the low-dose and high-dose studies. Data are means ± 95% confidence intervals. AA represents ascorbic acid, CA represents calcium ascorbate, † represents *p* < 0.05 difference from baseline, * represents *p* < 0.05 difference between treatments, while ⁑ represents *p* > 0.015 to *p* < 0.10 difference between treatments.

**Figure 4:**
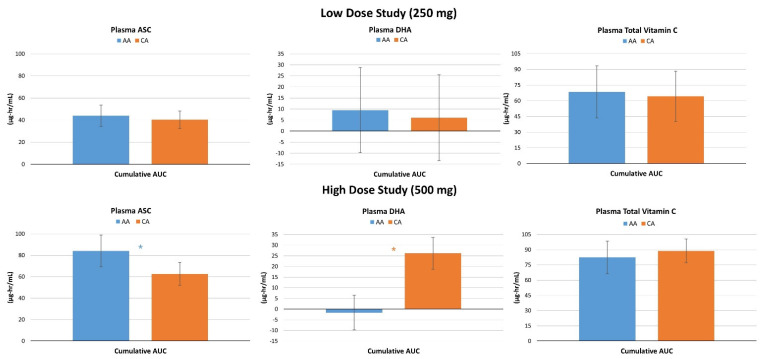
The area under the curve (AUC) changes from baseline for plasma ascorbic acid (ASC), dehydroascorbic acid (DHA), and total vitamin C levels for the low-dose and high-dose studies. Data are means ± 95% confidence intervals. AA represents ascorbic acid, CA represents calcium ascorbate. * represents *p* < 0.05 difference between treatments.

Tables S3a and S3b present plasma ASC, DHA, and total vitamin C levels for the low- and high-dose studies. Analysis of low-dose results revealed multivariate Wilk’s Lambda time (*p* < 0.001, η_p_^2^ = 0.329, large effect) but no treatment x time effects (*p* = 0.883, η_p_^2^ = 0.006, small effect). Significant time (*p* < 0.01) but no treatment x time effects in plasma ASC (*p* = 0.478, η_p_^2^ = 0.010, small effect), DHA (*p* = 0.779, η_p_^2^ = 0.002, small effect), or total vitamin C (*p* = 0.817, η_p_^2^ = 0.002, small effect) were shown via univariate analysis. Plasma ASC and total vitamin C levels were augmented above baseline at each data point (see Figure 3). Conversely, no significant between-treatment differences were observed. Likewise, no significant differences in plasma ASC, DHA, or total vitamin C AUC values (see Figure 4) were found. Analysis of the high-dose study results conveyed a significant multivariate time (*p* < 0.001, η_p_^2^ = 0.447, large effect) and treatment x time effect in plasma ASC, DHA, and total vitamin C levels (*p* < 0.001, η_p_^2^ = 0.031, small effect). Significant time (*p* < 0.001) but no treatment x time effects in plasma ASC (*p* = 0.439, η_p_^2^ = 0.010, small effect) or total vitamin C (*p* = 0.815, η_p_^2^ = 0.004, small effect) were revealed by univariate analysis. A significant interaction was noted in DHA levels (*p* = 0.001, η_p_^2^ = 0.049, small to moderate effect). Pairwise comparison conveyed baseline DHA levels were significantly lower with CA treatment (−0.84 µg/mL [−1.3, −0.5], *p* < 0.001). The average rate of change from baseline analysis with 95% CIs showed that ASC and total vitamin C levels significantly increased over time with no between-treatment differences, while DHA levels significantly augmented over time with CA treatment while not increased with AA treatment. No between-treatment differences were seen in low-dose ASC, DHA, or total vitamin C AUC values. However, CA yielded significantly higher DHA AUC values compared to AA, while AA yielded higher ASC compared to CA, when consuming 500 mg doses.

### 4.4. Lymphocyte Concentrations

Tables S4a and S4b present lymphocyte ASC, DHA, and total vitamin C concentrations for the low and high-dose studies. No overall multivariate time (*p* = 0.295, η_p_^2^ = 0.013) or treatment x time effects (*p* = 0.870, η_p_^2^ = 0.006) or univariate treatment x time effects in ASC (*p* = 0.618), DHA (*p* = 0.698), or total vitamin C (*p* = 0.553) levels were observed following low-dose supplementation. Similarly, no significant differences were observed between treatments in total AUC values for ASC (*p* = 0.935), DHA (*p* = 0.296), or total vitamin C (*p* = 0.729) after low-dose ingestion. Conversely, high-dose supplementation resulted in significant multivariate time (*p* < 0.001, η_p_^2^ = 0.156, large effect) and treatment x time effects (*p* < 0.001, η_p_^2^ = 0.034, small effect). Significant treatment x time effects in DHA (*p* < 0.001) and total vitamin C (*p* = 0.052) were demonstrated via univariate analysis. The pairwise comparison revealed that ASC was lower in the CA treatment at baseline (*p* = 0.015) and tended to be lower at 32 h (*p* = 0.069) after ingestion; DHA levels were lower with CA at 24 h (*p* = 0.039); and total vitamin C levels were lower with CA after 24 h (*p* = 0.012) and 32 h (*p* = 0.041) after ingestion. Analysis of AUC values revealed significant differences between treatments in ASC (*p* = 0.047), DHA (*p* = 0.026), and total vitamin C (*p* = 0.026) levels.

**Figure 5:**
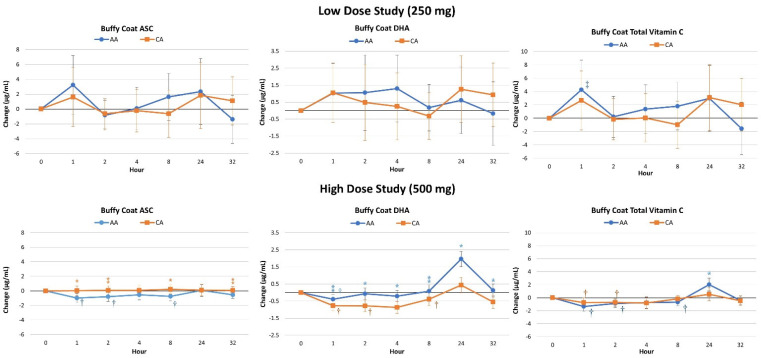
Changes in lymphocyte plasma ascorbic acid (ASC), dehydroascorbic acid (DHA), and total vitamin C levels for low- and high-dose studies. Data are means ± 95% confidence intervals. AA represents ascorbic acid, CA represents calcium ascorbate, † represents *p* < 0.05 difference from baseline, ‡ represents *p* > 0.05–*p* < 0.10 difference from baseline. * represents *p* < 0.05 difference between treatments while ⁑ represents *p* > 0.015 to *p* < 0.10 difference between treatments.

Figure 5 shows the average rate of change from baseline with 95% CI’s. Significant multivariate time effects (*p* < 0.001, η_p_^2^ = 0.329, large effect) were observed with no overall interaction effects (*p* = 0.883, η_p_^2^ = 0.006, small effect) in the low-dose study. Univariate analysis paralleled these findings. No significant differences were demonstrated between treatments in ASC (*p* = 0.742), DHA (*p* = 0.913), or total vitamin C (*p* = 0.793) AUC values after ingesting the low-dose ingestion. Conversely, significant time (*p* < 0.001, η_p_^2^ = 0.477, large effect) and treatment x time effects (*p* = 0.001, η_p_^2^ = 0.031) were observed in the high-dose study. Univariate analysis found time effects in ASC, DHA, and total vitamin C concentrations. However, a significant interaction effect was observed in changes in DHA levels (*p* < 0.001, η_p_^2^ = 0.049). Pairwise comparisons indicated that all markers of vitamin C metabolism were enhanced at most time points. Ingestion of CA resulted in lower ASC but higher DHA levels with slightly higher total vitamin C levels during the first four hours after ingestion. However, no significant between-treatment differences were displayed at individual data points. Analysis of delta AUC values revealed significant differences between treatments in DHA (*p* < 0.001) with no significant differences in ASC (*p* = 0.123) or total vitamin C (*p* = 0.346) levels.

### 4.5. Cell Blood Count Analysis

Tables S5a and S5b present the CBC results for both studies, respectively. In the low-dose study, significant overall effects over time were observed (*p* < 0.001, η_p_^2^ = 0.160) with no significant differences between treatments (*p* = 0.885, η_p_^2^ = 0.021). Significant effects over time in white blood cells (*p* <0.001, η_p_^2^ = 0.148, large effect), red blood cells (*p* < 0.001, η_p_^2^ = 0.130), hemoglobin (*p* < 0.001, η_p_^2^ = 0.111), hematocrit (*p* < 0.001, η_p_^2^ = 0.088), neutrophils (*p* < 0.001, η_p_^2^ = 0.294), lymphocytes (*p* < 0.001, η_p_^2^ = 0.273), monocytes (*p* < 0.001, η_p_^2^ = 0.163), eosinophils (*p* = 0.011, η_p_^2^ = 0.064), basophils (*p* = 0.001, η_p_^2^ = 0.047), and platelets (*p* < 0.001, η_p_^2^ = 0.057) were revealed via univariate analysis. Contrarily, no significant treatment x time interaction effects were noted among any whole cell blood counts. Pairwise comparisons revealed no significant between-treatment differences in any CBC marker.

**Figure 6:**
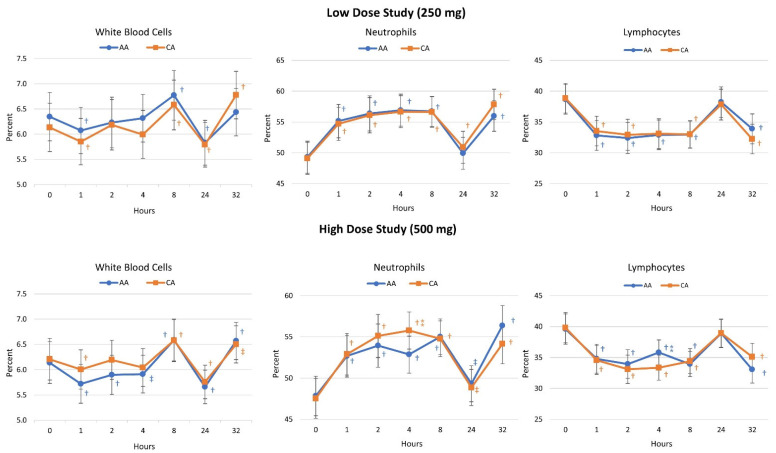
Changes in selected cell blood counts for the low and high-dose studies. Data are means ± 95% confidence intervals. AA represents ascorbic acid, CA represents calcium ascorbate, † represents *p* < 0.05 difference from baseline, ‡ represents *p* > 0.05–*p* < 0.10 difference from baseline. ⁑ represents *p* > 0.015 to *p* < 0.10 difference between treatments.

In the high-dose study, significant overall effects over time were observed (*p* < 0.001, η_p_^2^ = 0.169) with no significant differences between treatments (*p* = 0.564, η_p_^2^ = 0.024). Significant time effects in white blood cells (*p* < 0.001, η_p_^2^ = 0.184), red blood cells (*p* < 0.001, η_p_^2^ = 0.117), hemoglobin (*p* < 0.001, η_p_^2^ = 0.131), hematocrit (*p* < 0.001, η_p_^2^ = 0.104), mean corpuscular hemoglobin content (*p* = 0.034, η_p_^2^ = 0.025), neutrophils (*p* < 0.001, η_p_^2^ = 0.313), lymphocytes (*p* < 0.001, η_p_^2^ = 0.292), monocytes (*p* < 0.001, η_p_^2^ = 0.122), eosinophils (*p* < 0.001, η_p_^2^ = 0.338), basophils (*p* = 0.002, η_p_^2^ = 0.040), and platelets (*p* = 0.002, η_p_^2^ = 0.042, small to) were revealed via univariate analysis. Significant treatment x time interaction effects were demonstrated in neutrophils (*p* = 0.028, η_p_^2^ = 0.030) and lymphocytes (*p* = 0.030, η_p_^2^ = 0.029) with no other interaction effects seen. Pairwise comparisons indicated that 4 h after ingestion, neutrophils tended to be higher (2.92% [−0.25, 6.1], *p* = 0.072) while lymphocytes tended to be lower 2.45% [−5.3, 0.4], *p* = 0.093) with CA ingestion (see Figure 6).

### 4.6. Pharmacokinetic Analysis

Table S5 presents the comprehensive PK analysis on plasma values adjusted for body mass and dosage. In the low-dose study, no significant between-treatment differences were demonstrated in vitamin C elimination, disappearance /appearance slope or rate, half-life, Cmax, Tmax, AUC, AUMC, MRT, Vd, Vss, CL area, elimination rates, or distribution/absorption rates. The Vd tended to be lower (*p* = 0.099) with CA, while the remaining PK variables were not significantly different between treatments. Total vitamin C half-life was significantly faster with CA (−17.41 h [−33.9, −0.903], *p* = 0.039), with no significant between-treatment differences observed in other PK variables. In the high-dose study, analysis of vitamin C distribution/absorption rate (0.059 1/h [−0.002, 0.121], *p* = 0.058) and slope (−0.026 1/h [−0.052, 0.001], *p* = 0.058) tended to show between-treatment differences. No significant between-treatment differences were demonstrated in PK DHA parameters. A significant decrease was noted in total vitamin C Vd (−94,999 mL [−180,784, −9216], *p* = 0.030) while Vd area (−29,743 mL [−62,002, 2517] *p* = 0.070) and relative Vd area (−427 mL/kg [222, −869], *p* = 0.058) tended to decrease. CL observed area (13.2 mL/h [−1.2, 27.7], *p* = 0.073) and CL exponential (3251 mL/h [−116, 6.617], *p* = 0.058) tended to be higher with CA. The remaining PK variables were not significantly different between treatments.

### 4.7. Neutrophil Functionality

**Figure 7:**
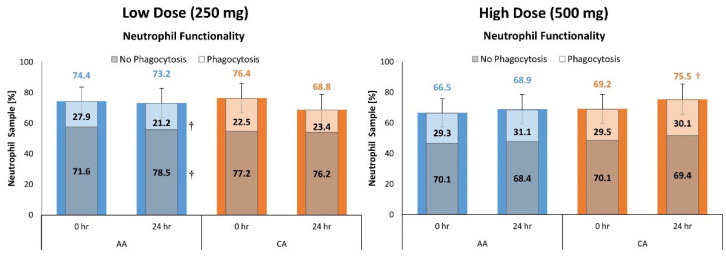
Comparison of Polymorphonuclear Leukocyte (PMN) functionality at baseline (hour 0) and 24 h for the low-dose (left panel) and high-dose (right panel) studies between treatment groups. Data are expressed as percent (%) of total PMN counts; the percentage of PMNs with or without phagocytosed bacteria are depicted within each total PMN count (%). Significant differences are indicated from baseline: † represents *p* < 0.05 difference from baseline.

Tables S6a and S6b present PMN functional assessment results for the low- and high-dose studies. The percentages indicate the percentage of neutrophils with and without phagocytosed bacteria. Overall, multivariate analysis revealed no time (*p* = 0.210, η_p_^2^ = 0.052) or treatment x time (*p* = 0.208, η_p_^2^ = 0.052) effects in the low-dose study. Univariate analysis showed a trend in neutrophil percent with (*p* = 0.070, η_p_^2^ = 0.038) and without phagocytosed bacteria (*p* = 0.068, η_p_^2^ = 0.038), which tended to interact with the phagocytosed-containing neutrophil percent in the CA group increasing significantly from baseline (6.78% [0.8, 12.8), *p* = 0.027), with no effects seen from AA ingestion (see Figure 7).

### 4.8. Lymphocyte Cell Differentiation

Given the more robust effects observed in the high-dose study, we performed a detailed lymphocyte cell differentiation (CD) analysis on 0 and 24 h post-ingestion samples in a subset of 33 participants. Table S7 presents the results of the cell differentiation (CD) analysis. Overall, multivariate analysis displayed time (*p* < 0.001, η_p_^2^ = 0.707) with no treatment x time interaction effects observed (*p* = 0.457, η_p_^2^ = 0.217). Time effects (*p* < 0.05) were revealed via univariate analysis in CD4+ | T helper cells, CD4+; IL2+ | T helper cells expressing IL2; CD4+; TNFa+ | T helper cells expressing TNFa, CD8+; CD38+ | Activated Cytotoxic T cells, CD8+; IFNg+ | Cytotoxic T cells expressing IFNg; and CD8+; TNFa+ | Cytotoxic T cells expressing TNFa. Treatments tended to interact over time in CD8+ | Cytotoxic T cells (*p* = 0.091, η_p_^2^ = 0.044) and CD16+ & CD56+ | Natural Killer Cells (*p* = 0.062, η_p_^2^ = 0.054). A trending decrease was noted for CD25+ & CD127+ | Regulatory T cells at 24 h after CA treatment (−6.09 [12.8, 0.6], *p* = 0.072), while CD16+ & CD56+ |Natural Killer Cells increased significantly at 24 h post-CA supplementation (12.2 [2.7, 21.7], *p* = 0.012).

### 4.9. Side Effects

No side effects from treatment ingestion were reported by any participants at any time or after the study.

## 5. Discussion

Individuals are generally recommended to consume fruits and vegetables to provide enough vitamin C to prevent deficiencies, other antioxidants, and support the immune system [35]. However, studies evaluating the effects of vitamin C supplementation on immune function and the incidence and severity of the common cold and some infections have been mixed, particularly at lower doses [36,37,38,39,40]. One reason for the inconsistent findings may be related to the bioavailability of different forms of vitamin C studied. Vitamin C trials comparing the PK profiles and immune effects of CA vs. AA or other forms of vitamin C have shown favorable responses to CA in plasma and leukocyte vitamin C retention and increased bioavailability in leukocytes in acute supplemental protocols [1,2,3,4,5,6]. Vitamin C is present in high concentrations in leukocytes. It is an important indicator of immune status and white blood cell functionality as it protects the cell from rapidly releasing reactive oxygen species (ROS) [41,42]. Neutrophils, the body’s most abundant leukocyte, are typically highly concentrated with vitamin C for optimal protection against invading pathogens [43]. In the present study, two simultaneous crossover studies were conducted to determine if low (250 mg) or higher (500 mg) doses of CA would differentially affect PK profiles and/or markers of immunity compared to AA in healthy male and female adults. No overall differences were observed between treatments in plasma or leukocyte ASC, DHA, or total vitamin C values when ingesting 250 mg of CA or AA. However, in concurrence with our hypothesis, supplementation with 500 mg of CA promoted greater conversion of ASC to DHA, significantly higher lymphocyte ASC levels, greater production of neutrophils and phagocytosis compared to pre-supplementation, a greater increase in certain natural killer cells, thereby enhancing immune function during a standard immune challenge.

### 5.1. Primary Outcomes

We examined the effects of 250 mg and 500 mg CA vs. AA on the pharmacokinetic profiles via concentration–time curves for plasma and lymphocyte AA and DHA. Findings indicate no differences between the different forms of vitamin C in AUC values of ASC, DHA, or total vitamin C with 250 mg supplementation. However, acute supplementation with 500 mg CA significantly enhanced ASC conversion to DHA, indicated by higher AUC values, in the plasma and greater retention of ASC in lymphocytes. In this regard, results indicate acute supplementation with lower doses of CA is not sufficient to elicit enhanced absorption and retention of ASC and/or DHA in plasma and/or lymphocytes compared to commercially available AA; however, a higher dose (500 mg) of CA did significantly enhance vitamin C absorption kinetics. In the low-dose study, plasma ASC and total vitamin C did increase significantly at one, two, and eight hours post-supplementation. However, these responses were not different between treatments, thus indicating no benefit of CA over AA at 250 mg. Results from the 500 mg study show significantly increased plasma DHA at most time points post-supplementation from baseline and compared to AA, mirrored in the cumulative AUC analysis. These results were replicated in the lymphocyte analysis with a maintenance in ASC and a decrease in DHA with CA supplementation, but a significantly increased DHA at most timepoints after AA supplementation.

The increase in plasma DHA with CA supplementation has been shown in previous trials incorporating higher vitamin C doses. For example, Mitmesser and researchers [3] provided evidence that shows 1000 mg CA can significantly improve plasma ascorbate concentration at 4, 8, and 24 h after supplementation, with significant increases also seen in leukocyte ascorbate concentration. The same research group completed a follow-up study with 40 healthy adults who supplemented 1000 mg CA compared to AA and found higher Cmax plasma and leukocyte vitamin C concentrations, though the mean percent change from baseline in plasma vitamin C was similar compared to AA control [1]. Similarly, Moyad and colleagues [2] found an increase in plasma vitamin C concentration, though it was not different from AA supplementation, but leukocyte vitamin C concentration was higher after 24 h. Though the current trial utilized lower doses of CA, increases in plasma ASC, DHA, and total vitamin C were still seen with higher DHA plasma concentrations. In considering the plasma results, the increased lymphocyte DHA concentration suggests that more ASC was oxidized into DHA, suggesting it was used as an antioxidant or enzyme cofactor [44]. DHA has an important metabolic role because it can be absorbed into tissues readily through ascorbic acid and glucose transporters [45] and then used to regenerate ascorbic acid [44]. Consequently, compared to ASC, there may be metabolic advantages to increasing blood DHA levels. Interestingly, Purpura and colleagues [8] reported that ingesting 500 mg of a liposomal coated source of L-ascorbic acid promoted a significantly higher Cmax (plasma +27%, leukocytes +20%, and 24 h AUC (plasma +21%, leukocytes +8%) compared to AA. While ASC, DHA, and detailed PK analysis were not provided, these findings suggested greater vitamin C absorption into plasma and lymphocytes.

In the present study, we analyzed ASC, DHA, and total vitamin C levels and conducted a detailed PK analysis to assess appearance, absorption, and clearance. The metabolism of vitamin C from plasma to lymphocytes relies on a gradient-driven transport mechanism in which ASC is initially oxidized to DHA and then immediately reduced back to ASC upon cell entry, which could help explain the increased ASC in lymphocytes after 500 mg CA supplementation [46]. Since some tissues prefer DHA due to greater transport capacity, converting ASC to DHA and circulating it in the blood may offer some advantage [44,45]. Calcium ascorbate may also exhibit higher bioavailability compared to AA due to the neutralizing activity of the calcium salt, which was initially developed to circumvent adverse gastric effects associated with the low pH of AA [47].

### 5.2. Secondary Outcomes

Since CA ingestion has been shown to enhance the vitamin C status of leukocytes and immune function with acute [2,3] and prolonged supplementation [4,5], there has been interest in the function and status of various immune cells (PBMCs, neutrophils, etc.) with vitamin C supplementation. Therefore, we assessed the effects of acute CA supplementation on baseline and 24 h post-supplementation phagocytic activity of neutrophils and leukocyte differentiation markers, inflammatory markers, cytokines, and natural killer cells in PBMC in response to exposure to a mitogen. Results from the 250 mg study revealed no effect of CA on neutrophil functionality as determined by assessment of the percentage of phagocytosis in neutrophils. However, supplementation with 500 mg CA increased neutrophil phagocytosis function from pre-ingestion values and promoted a larger enhancement in CD16+ & CD56+ natural killer cells. The percentage of phagocytosed bacteria significantly increased at 24 h from baseline in the CA group, with no effect seen in the AA group, which was a novel finding, as these are the first studies to evaluate the phagocytic activity of neutrophils in response to vitamin C ingestion. Conversely, the low-dose study showed a significant reduction in phagocytosis in the AA group at 24 h compared to baseline, with no differing effects seen in the CA group. Neutrophils optimally function with adequate levels of vitamin C available [35,48], which could help explain the increase in neutrophils over time after CA and AA treatment. The increase in phagocytosis with CA treatment could be explained by the effect vitamin C has on neutrophils. Vitamin C promotes microtubule organelle assembly in leukocytes, thus increasing chemotactic activity and promoting phagocytosis [48]. Additionally, only at four hours post-supplementation did CA increase neutrophil concentration in the serum, however, this cannot be overlooked. Neutrophil elevation after four hours of CA supplementation promotes enhanced immune function over a prolonged, yet acute, period. These have implications for individuals who undergo high training volumes with prolonged exercise bouts and might help lessen the risk of URTI or other immune-related challenges in athletes. In addition, our results track with previous findings indicating a circadian rhythm in neutrophil response [49,50,51], showcased by the gradual increase over eight hours, then a near return to baseline after 24 h. However, the separation between treatments at four hours post-ingestion suggests possible enhancing effects of CA. More research is needed on how CA can affect neutrophil concentration in response to an immunological stressor compared to a placebo, especially in athletes or those exposed to chronic immune stress.

In addition to neutrophilic effects, a sub-analysis showed increased CD16+ & CD56+ natural killer cells after 500 mg CA ingestion, another in vivo finding with CA supplementation. Natural killer cells originate from lymphoblasts and are integral immune cells in identifying and removing pathogens [52,53]. Similarly, a previous clinical trial found enhanced natural killer cell production after 8–24 h post-AA ingestion in healthy subjects [54]. Heuser and Vojdani [55] corroborated these findings in patients with chronic toxin exposure history. Furthermore, an in vitro study showed an increased concentration of natural killer cells in a culture medium after treatment with AA [56]; however, another study indicated the inhibitory effects of AA on natural killer cell recruitment and activity [57]. CD16+ natural killer cells mediate antibody-dependent cytotoxicity in the cell [58], with CD56+ cells being stratified in two forms (CD56^bright^ and CD56^dim^) that modulate immunoregulation and pro-inflammatory cytokine production, with the latter being cytotoxic [53,59]. Together, the two cell types work to remove pathogens or other invading material and eliminate infected cells. Enhancing natural killer cell differentiation suggests that 500 mg CA promoted a beneficial immune response.

### 5.3. Limitations and Future Directions

Though significant findings were apparent from the high-dose study, the interventions lacked a true immune challenge. Participants rested passively for 32 h while undergoing blood donation and receiving standardized diets. Additional trials are needed to assess the immune effects of CA on immune status after an immune stressor (i.e., high-intensity exercise bout). Additionally, only apparently healthy adults aged 18–60 years old were recruited for the study. Future work might consider: (1) adding a true placebo group, (2) assessing whether supplementing with several smaller doses throughout the day may affect results, (3) whether a longer supplementation period may promote added benefit, (4) whether sex differences in immune responses (e.g., NK and CD markers) or plasma volume affect results, (5) examining mechanisms of AA and conversion to DHA and individual variability may affect responses, and (6) mechanisms of how calcium may protect absorption, delivery, and/or uptake of ascorbic acid. Moreover, consider evaluating athletes who undergo high training volumes or are highly susceptible to upper respiratory tract infections (i.e., ultra-endurance athletes, ironman competitors, etc.). Lastly, a protocol was developed for neutrophil and PBMC extraction and analysis based on the methodologies of previous studies. This protocol needs to be further validated in future work. It is imperative to delineate that this study was done in healthy individuals with no challenging immune stress. We expect the results to be more impressive if an immune challenge were provided. Since intense and prolonged exercise promotes short-term immunosuppression, we recommend following up this work with an exercise-induced immune challenge. While daily vitamin C supplementation can promote general benefits to the immune system to help individuals manage stress and better respond to random exposure to pathogens, showing that 500 mg of CA would provide more benefit to the immune system in the days following intense exercise could support better different needs for those individuals who have physically active work demands, military personnel, and athletes. The protocol used to isolate and analyze the neutrophils and PBMCs must be validated in further research. In addition, further work is warranted on the effects of various doses of CA on lymphocyte differentiation, given the findings on natural killer cells in the current study.

## 6. Conclusions

Ingesting CA at 250 mg does not appear to stimulate significant effect changes in plasma or lymphocyte vitamin C, PK, or neutrophil function compared to 250 mg AA. Conversely, there was evidence that ingestion of 500 mg CA significantly heightened ASC conversion to DHA, increased neutrophils during the first 8 h of the PK study, altered weight-adjusted PK profiles, suggesting greater volume distribution and clearance from the blood, increased neutrophil functionality, and promoted an increase in natural killer cells. These findings indicate that 500 mg of CA may promote additional immune benefits compared to 500 mg of AA ingestion. Additionally, individuals may not need to ingest 1000 mg of vitamin C to observe some benefits on immune markers.

## Data Availability

Data and statistical analyses are available for non-commercial scientific inquiry and/or educational if request and use does not violate IRB restrictions and/or research agreement terms.

## Supplementary Materials

The following supporting information can be downloaded at: https://www.mdpi.com/article/10.3390/nu16193358/s1, Supplemental Tables: Table S1a. Baseline demographics for low-dose study, Table S1b. Baseline demographics for high-dose study, Table S2a. Baseline serum health markers for the low-dose study, Table S2b. Baseline serum health markers for the high-dose study, Table S3a. Plasma vitamin C concentrations observed in the low-dose study, Table S3b. Plasma vitamin C concentrations observed in the high-dose study, Table S3c. Low dose study plasma vitamin C concentration Area Under the Curve (AUC), Table S3d. High-dose study plasma vitamin C concentration Area Under the Curve (AUC), Table S4a. Leukocyte AA, DHA, and total vitamin C levels for the low-dose study determined from buffy coat samples, Table S4b. Leukocyte AA, DHA, and total vitamin C levels for the high-dose study determined from buffy coat samples, Table S4c. Leukocyte AA, DHA, and total vitamin C levels for the low-dose study were determined from buffy coat samples, Table S4d. Leukocyte AA, DHA, and total vitamin C area under the curve values for the high-dose study determined from buffy coat samples, Table S5a. Whole blood markers in response to low-dose supplementation, Table S5b. Whole blood markers in response to high-dose supplementation, Table S6. Dose and body weight-adjusted plasma AA, DHA, and total vitamin C pharmacokinetic analysis from plasma values, Table S7a. Low-dose study polymorphonuclear leukocyte (PMN) functionality assessment, Table S7b. High-dose study polymorphonuclear leukocyte (PMN) functionality assessment, Table S8. Peripheral Blood Mononuclear Cells (PBMC) cell differentiation analysis results. Supplemental File S2: Detailed blood processing procedures.
