# Supplementary material for: Comparative Effectiveness of Ascorbic Acid vs. Calcium Ascorbate Ingestion on Pharmacokinetic Profiles and Immune Biomarkers in Healthy Adults: A Preliminary Study
Source: Nutrients. 2024 Oct 2;16(19):3358. doi: 10.3390/nu16193358 (PMC11479081; doi:10.3390/nu16193358)
Supplement: Supplementary file 1 [file nutrients-16-03358-s001.zip › Supplemental File S2.pdf]

Impacts of Ascorbic Acid vs Calcium Ascorbate Supplementation on Pharmacokinetic Status and Immune Biomarkers in Healthy Adults

Broderick Dickerson[1], Drew E. Gonzalez[1], Ryan Sowinski[1], Dante Xing[1], Megan Leonard[1], Jacob Kendra[1], Victoria Jenkins[1], Siddharth Gopalakrishnan[1], Choongsung Yoo[1], Joungbo Ko[1], Syamkumar Sivasankara Pillai[2], Jigna R. Bhamore[2], Bhimanagouda S. Patil[2], Gus A. Wright[3], Christopher J. Rasmussen[1], Richard B. Kreider[1,2*]

[1] Exercise & Sport Nutrition Lab, Department of Kinesiology and Sport Management, Texas A&M University, College Station, TX 77843, USA
[2] Vegetable and Fruit Improvement Center, Department of Horticulture, Texas A&M University, College Station, TX USA
[3] Flow Cytometry Facility, Department of Veterinary Pathobiology, Texas A&M University, College Station, TX, 77843-4467 USA

Detailed Blood Processing Procedures

PROTOCOL 1: **Blood Collection and Processing**

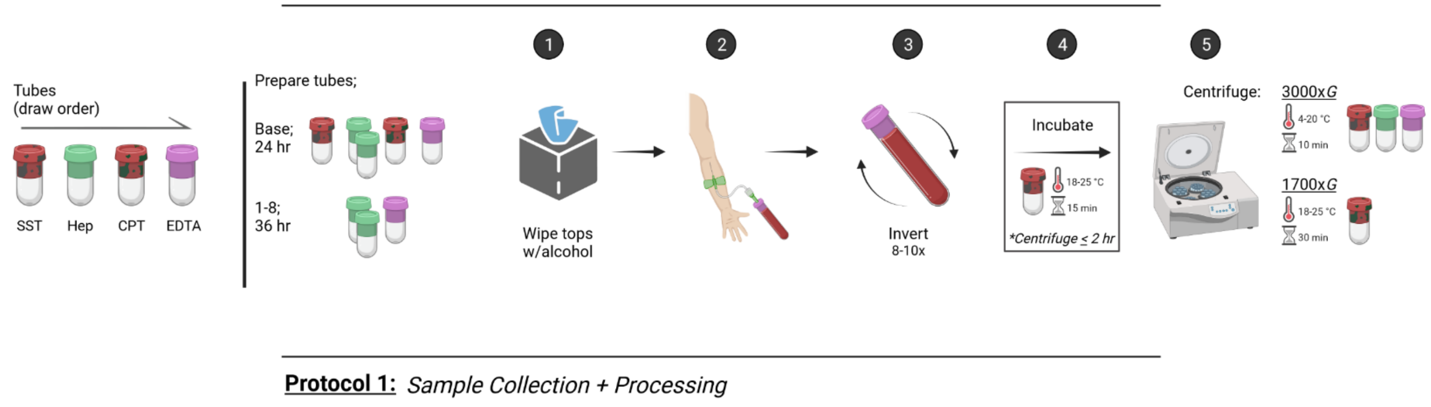

Blood Collection

Keep CP Tubes at **Room Temperature** and upright for at least 30 min before use
(Crucial for functionality of CPT Ficoll layer that processing be done at **room temperature, 18-25°C)*
Tubes (in draw order)

1| Wipe tops with alcohol
2| Collect Blood
3| Invert gently 8-10x each
| Incubate SST for 15 min at **Room Temperature**
| Centrifuge within **2 hr**
4| Centrifuge: G

a. SST; EDTA; Hep - 3,000, 10 min, 4-20°C
b. CPT - 1,700, 30 min, **Room Temperature** (18-25°C)

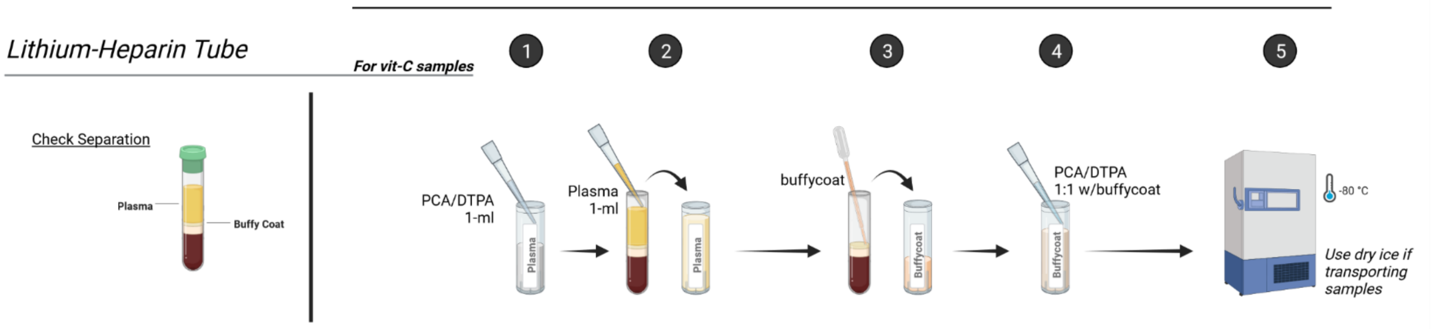

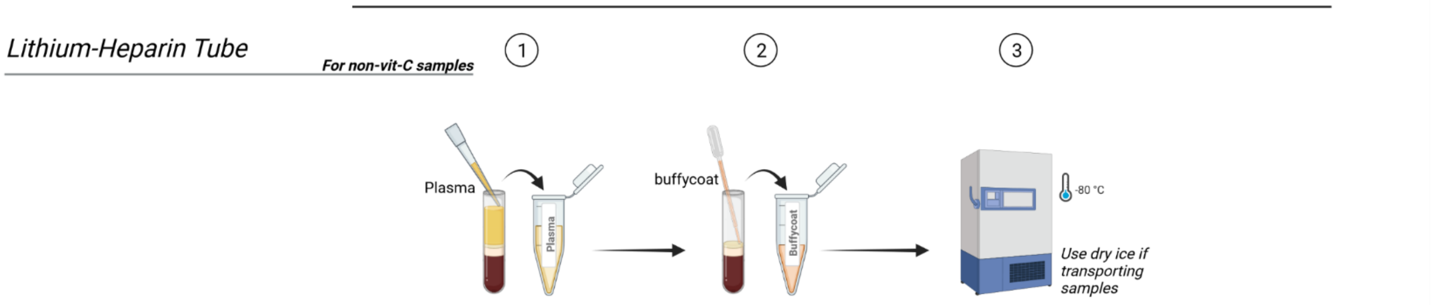

Protocol 1: *Sample Collection + Processing*

Lithium-Heparin Tube Processing

Prepare labeled 2-ml cryovials; per subject

a. 4-6x plasma

b. 2x buffycoat

1| Add 1-ml PCA/DTPA solution to each plasma vial

2| Aliquot 1-ml of plasma into each labeled vial

3| Aliquot buffycoat from each Hep tube into individual vials

4| Add PCA/DTPA solution to each vial, in a 1:1 ratio with the buffycoat

5| Store the sample at **-80°C**

| Use dry ice to transport samples to other labs

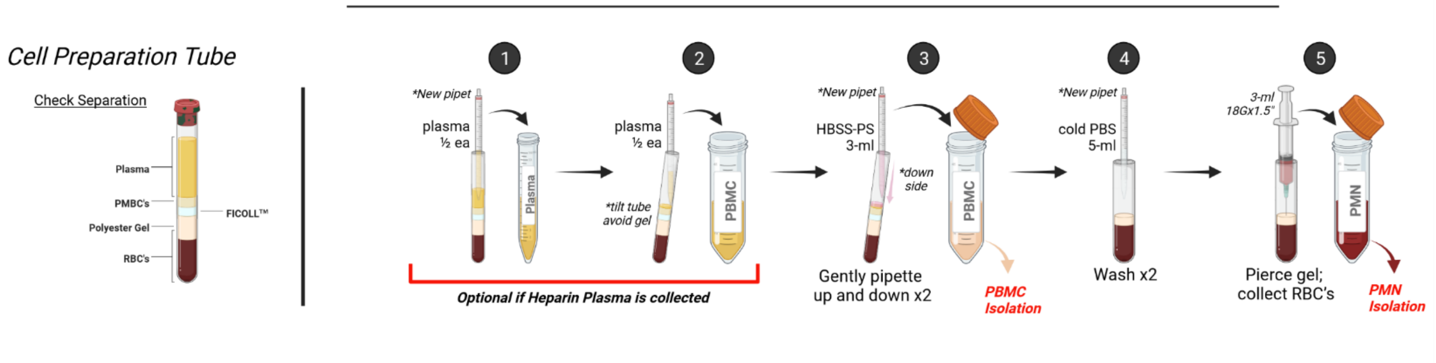

Protocol 1: *Sample Collection + Processing*

CP Tube Processing

Prepare 2x 50-ml, 1x 15-ml conical tubes per subject

Label as: 'PBMC', 'PMN', and 'Plasma', respectively

Check for proper separation of tube components by density gradient

1| With a sterile pipet, transfer ½ the plasma from each CP Tube to a 'Plasma" tube (**1/subject**)

PBMCs

2| Transfer remaining plasma to a 'PBMC' tube; tilting the tube, touching the side, avoid the gel

3| With a new sterile pipet, add 3-ml ***HBSS-PS*** to each CP Tube, dispense down the side of the tube, rinsing it

| Gently pipette up and down 2x, to capture all cells

| Using the same pipet, avoid pipetting gel, transfer all ***HBSS-PS***/sample mixture to 'PBMC' tube

PMNs

4| With a new sterile pipet, wash upper portion of the gel 2x with 5-ml of cold ***PBS***

5| Use a 10-ml syringe with a 1.5 in., 18 gauge, needle to pierce the gel and collect RBC's

| Transfer RBC's to a 'PMN' tube

PROTOCOL 2: **Isolation of PBMCs and PMNs**

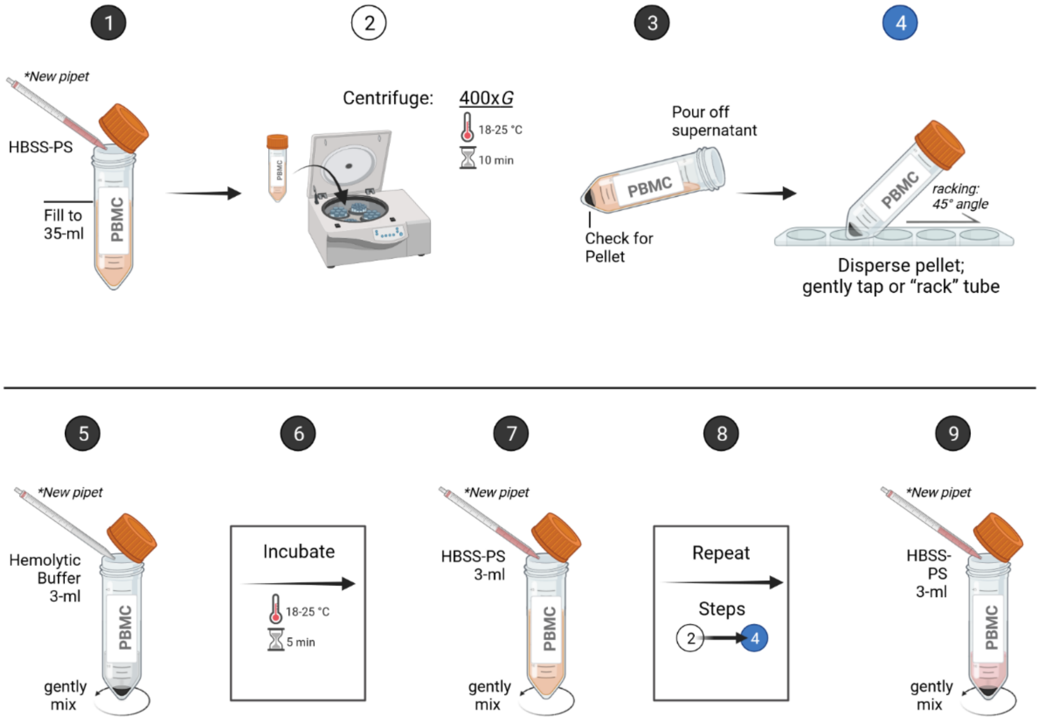

Go to Protocol 3

Protocol 2: *PBMC Isolation*

PBMC Isolation

1| Using a new sterile pipet, add ***HBSS-PS*** to 'PBMC' tube, bringing the volume to 35-ml
2| Centrifuge for 10 min at 330×*G*, **Room Temperature** (18-25°C)
3| Check for an intact pellet
4| Pour off supernatant and disperse cell pellet by gently tapping tube or "racking"
(racking = drawing conical tube, 45° angle, across the top of a plastic tube rack)
**(Do not vortex samples to minimize damage to cells)*
5| With a new sterile pipet, add 3-ml ***Hemolytic Buffer*** and gently mix
6| Incubate 5 min at **Room Temperature** (18-25°C)
7| With a new sterile pipet, add 3-ml ***HBSS-PS*** and invert, or mix gently
8| Centrifuge for 10 min at 330×*G*, **Room Temperature** (18-25°C)
| Check for an intact pellet
| Pour off supernatant and disperse cell pellet by gently tapping tube or "racking" *(Do not vortex)*
9| With a new sterile pipet, add 10-ml ***HBSS-PS*** to cell pellet

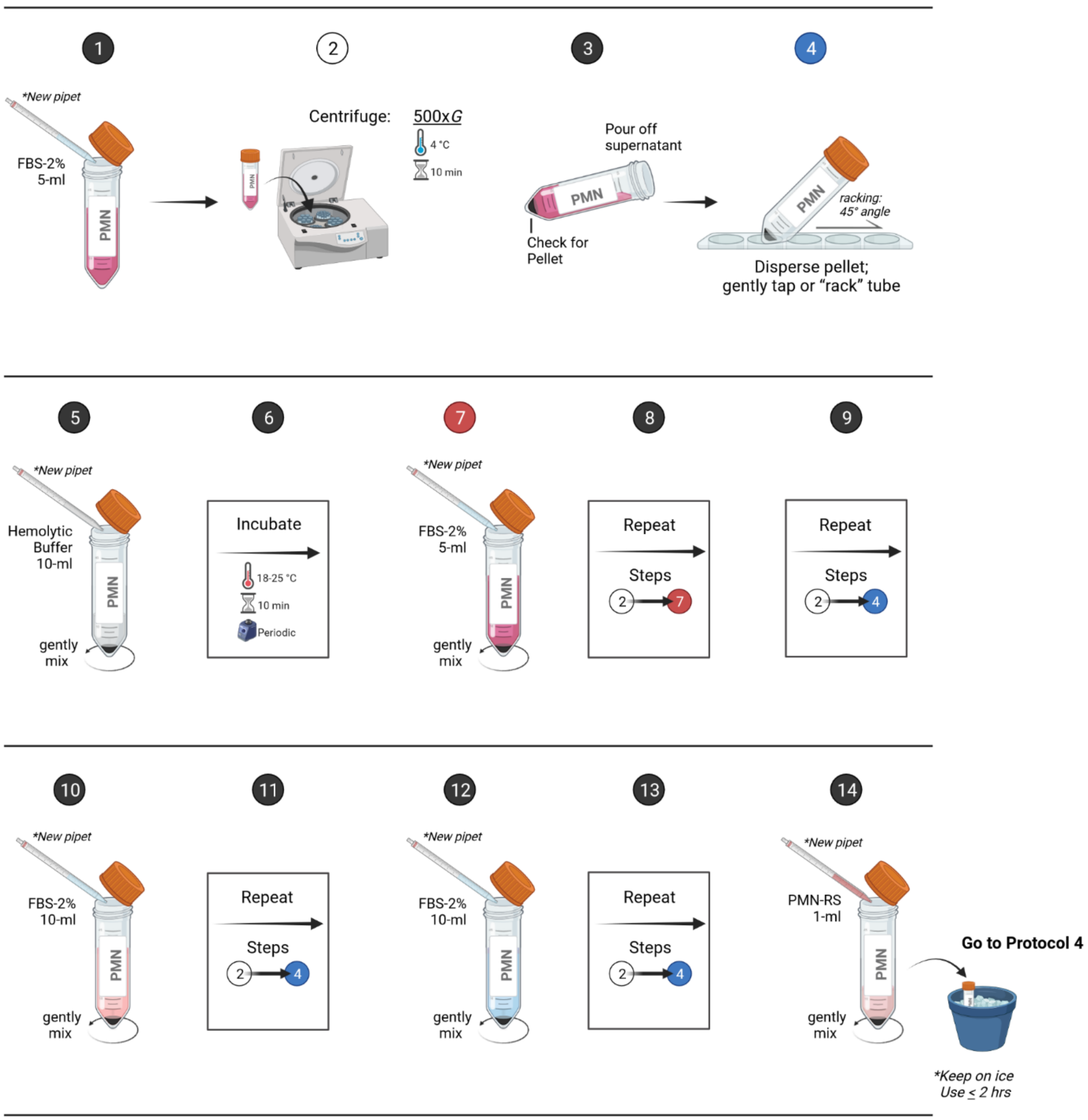

Protocol 2: *PMN Isolation*

PMN (Neutrophil) Isolation

1| With a new sterile pipet, add 5-ml cold PBS containing 2% FBS to ‘PMN’ tube
2| Centrifuge for 10 min at 400×*G*, **4°C**
3| Remove supernatant
4| Disperse cell pellet by gently tapping tube or “racking”
5| Using a new sterile pipet, add 10-ml EL buffer
6| Incubate for 10 min at **Room Temperature** (18-25°C), intermittently vortexing
7| Repeat steps 1-3
8| With a new sterile pipet, add 1-ml ‘PMN-RS’ mixture
9| Keep on ice, **use within 2 hr**

PROTOCOL 3: **Cryopreservation of PBMCs**

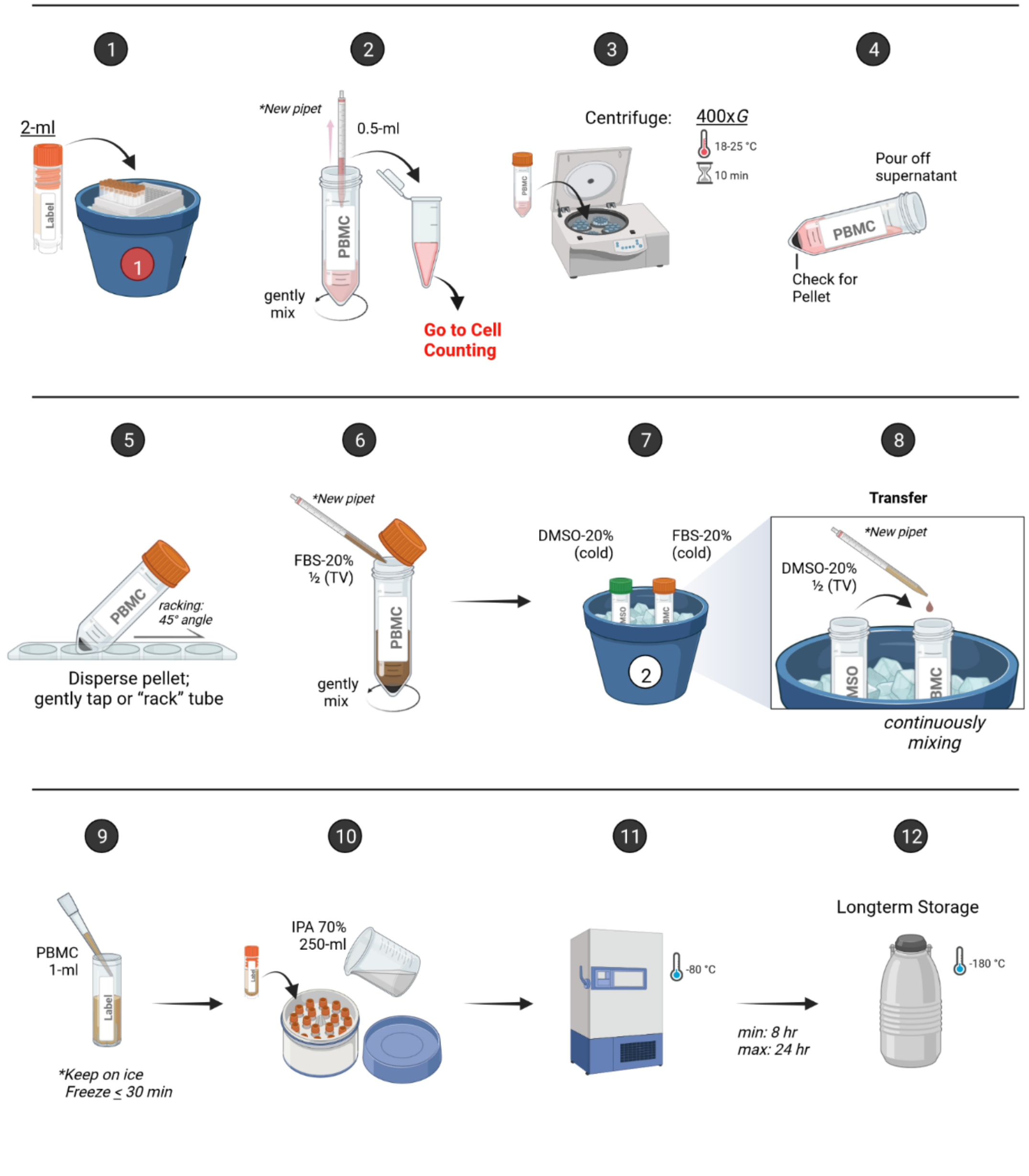

Protocol 3: *PBMC Cryopreservation*

Cryopreserve PBMCs

Prepare 2 melting ice baths in a shallow tray

1| Put 2-ml cryovials, with subject codes, in a cryovial holder and place in one ice bath

2| Pipet 20-µl of resuspended PBMCs, from 'PBMC' tube, onto the hemacytometer

| Count cells located in the four outer squares of the hemacytometer, using an optical microscope

| Calculate cell count: **total cell number (cells/ml) = avg cell count × 10^4 × 5**

| Calculate medium volume needed for a cell concentration of 4 × 10^6 cells/ml: **volume of medium (ml) = total cell number/(4 × 10^6)**

3| Centrifuge 'PMBC' tube for 10 min at 400×*G*, **Room Temperature** (18-25°C)

4| Pour off supernatant

5| Gently disperse cell pellet

6| With a new sterile pipet, add FBS-20% equal to ½ total volume (calculated in step 2)

| Place 'PBMC' tubes, containing lymphocyte suspension, in the second ice bath

7| With a new sterile pipet, add DMSO-20% equal to ½ total volume (calculated in step 2) to a new 50-ml tube, labeled 'DMSO'

| Place tubes in the same ice bath with 'PBMC' tubes

**(All tubes should stay on ice during the following steps)*

8| With a new sterile pipet, slowly add 'DMSO' tube contents dropwise, continuously mixing to prevent gradients

**(Volume of medium added to the 'PMBC' tube should be: Total Volume (TV) = ½TV [FBS-20%] + ½TV [DMSO-20%])*

9| Using the same pipet, transfer **1-ml** of suspension to appropriate 2-ml cryovials (pre-chilled and labeled)

| Screw caps tightly

| Keep cryovials on ice water until freezing, **within 30 min**

10| Transfer vials into Mr. frosty container

11| Store at **-80°C** (min: 8 hr, max: 24 hr)

12| Move to liquid nitrogen for long-term storage

**(Typically, 2-3 cryovials (4 to 6 million PBMCs/ea) are obtained from a single CPT using this protocol)*

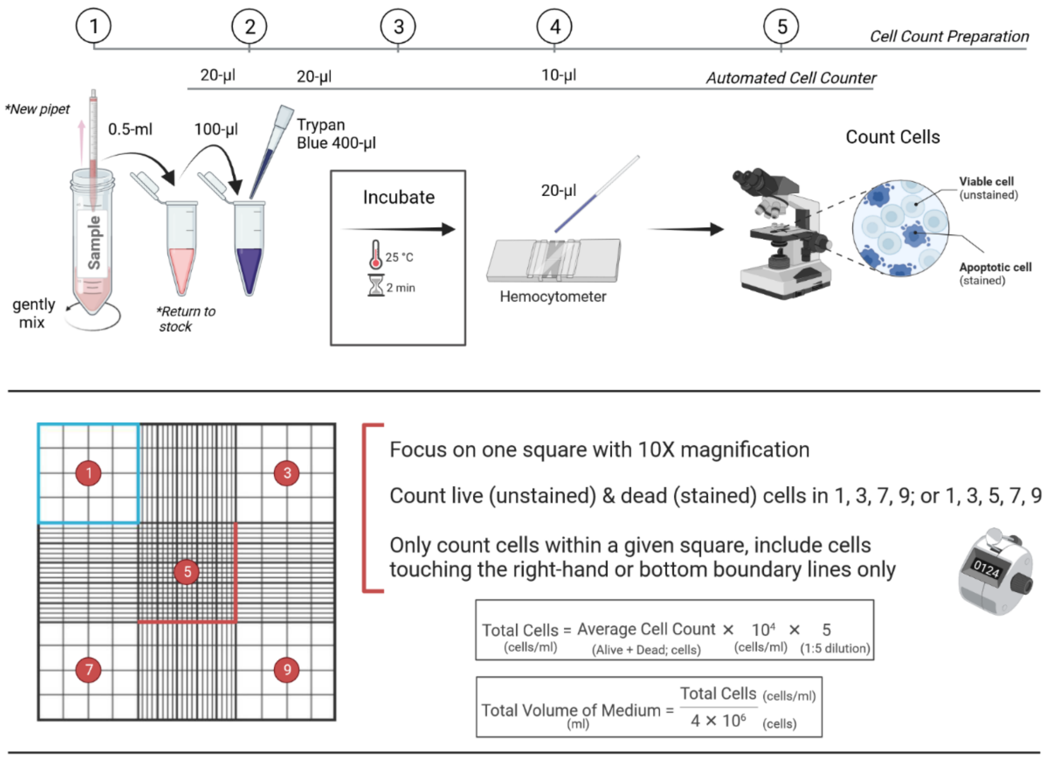

Cell Counting Procedure + Calculations

PROTOCOL 4: Preparation of **PMNs for Immunophenotyping**

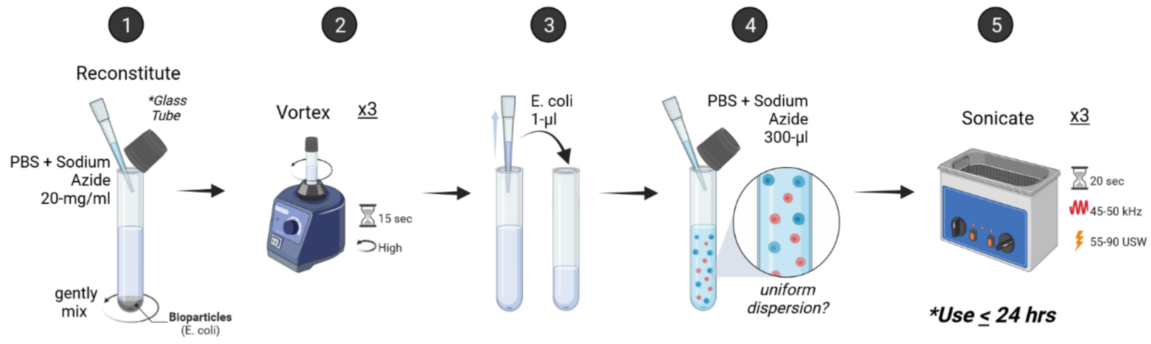

AF488-labeled E. Coli Preparation

Prepare AF488-labeled E. coli Bioparticles:

1| Reconstitute in a glass tube or vial at 20mg/ml in PBS (without sodium azide)
2| Cap tube, gently swirl particles into suspension
3| Vigorously vortex particles 3 × 15 sec (highest setting)
**(Reconstituted suspensions should be used within one day; Protect from light)*
4| Dilute a small volume of the particle suspension with PBS ~50-300 fold and inspect for uniformity of dispersion
5| Sonicate just before use

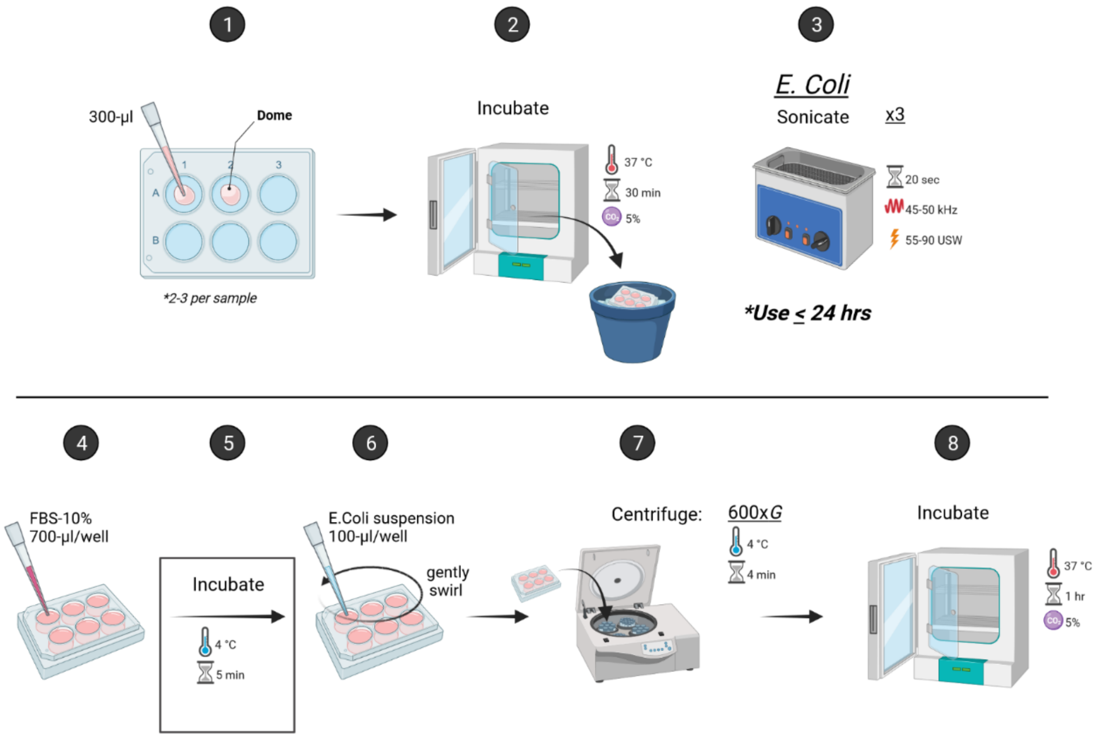

Protocol 4: *Neutrophil Plating + Infection*

Plating & Infecting primary adherent human neutrophils

Add 25-mm tissue culture plastic coverslips to 6-well plates

1| Gently pipet 300-µl cell suspension onto the middle of the coverslips, allowing it to form "a dome"

**(2-3 coverslips per condition normally required for sufficient number of cells)*

2| Incubate for 30 min in a humidified incubator at **37°C**, 5% CO_2

| Remove plates from incubator and place on ice, or pre-chilled (**4°C**) shipping packs

3| Sonicate E. Coli; 3 x 20 seconds; 45-50 kilohertz; 55-90 ultrasonic watts (until suspended homogeneously)

4| Add 700-µl/well RPMI + 10% FBS

5| Incubate for 5 min at **4°C**

6| Add 100-µl/well of AF488-labeled E.coli suspension; Gently swirl plate to mix

7| Centrifuge plates for 4 min at 600×*G*, **4°C** (with swinging bucket rotor and microplate carriers)

8| Return plates to incubator for **1 hr** at **37°C**, 5% CO_2

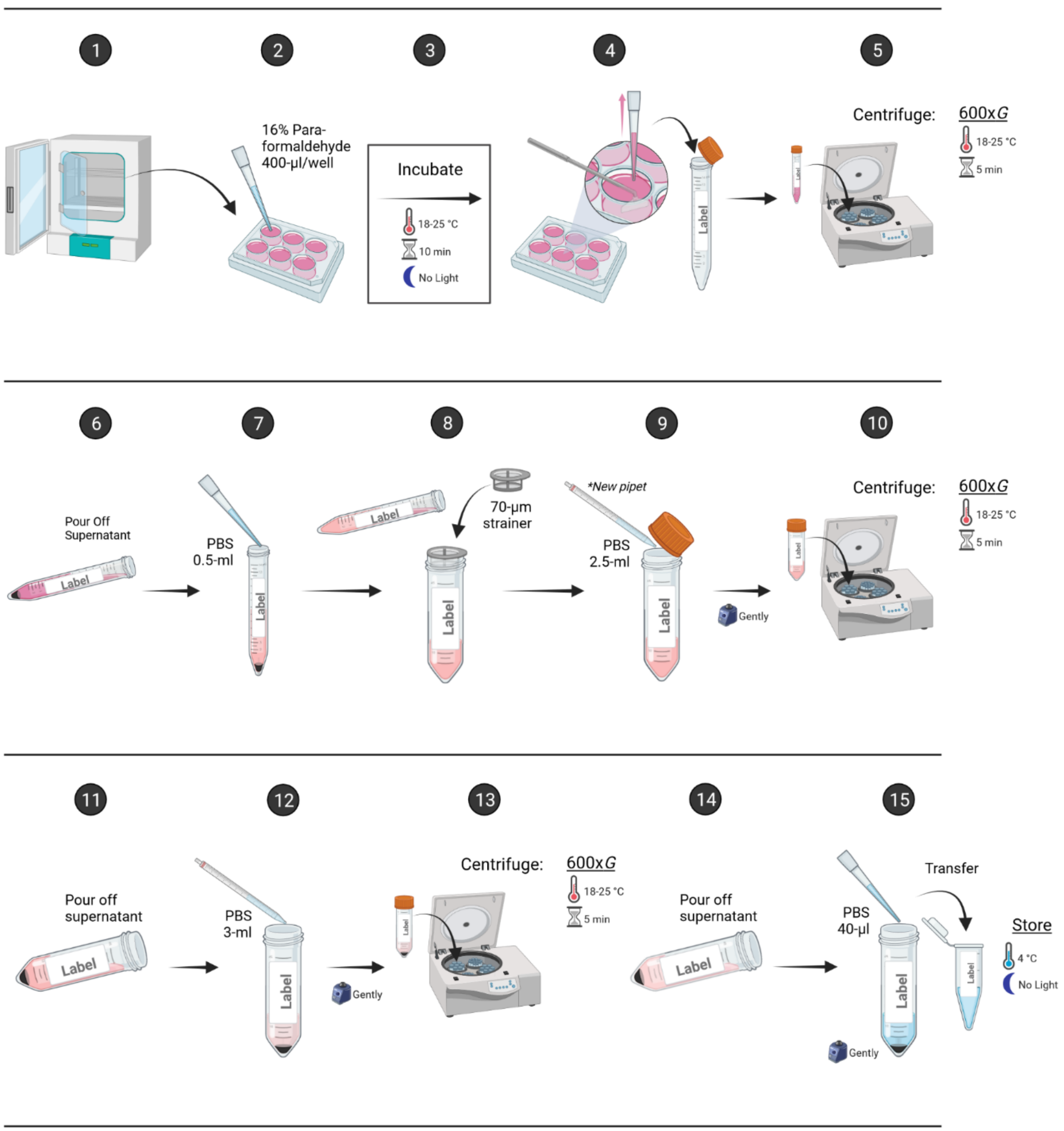

Protocol 4: *Neutrophil Fixation + Collection*

Fixation and Collection

1| Label 15-ml conical tubes, 1/subject
2| Remove plates from incubator
| Add 400-µl of 16% paraformaldehyde solution (final concentration of 4%)
3| Incubate for 10 min at **Room Temperature** (18-25°C), protected from light
4| Remove neutrophils from coverslips using a cell scraper
**(collect replicate wells in a single labeled 15-ml conical tube per subject)*
5| Centrifuge for 5 min at 600×*G*, **Room Temperature** (18-25°C)
6| Remove supernatant
7| Add 0.5-ml PBS
8| Pour through a 70-µm cell strainer to remove any clumps
9| Add 2.5-ml PBS, vortex gently
10| Centrifuge for 5 min at 600×*G*, **Room Temperature** (18-25°C)
11| Remove supernatant
12| Add 3-ml PBS, vortex gently
13| Centrifuge for 5 min at 600×*G*, **Room Temperature** (18-25°C)
14| Remove Supernatant
| Repeat steps 12-15, if needed
15| Add 40-µl PBS, vortex gently
| Transfer to 1.5-ml Eppendorf tubes (labeled) and store at **4°C** until analysis
**(Transport should be on ice and protected from light)*

PROTOCOL 5: Thawing and Preparing PBMC's

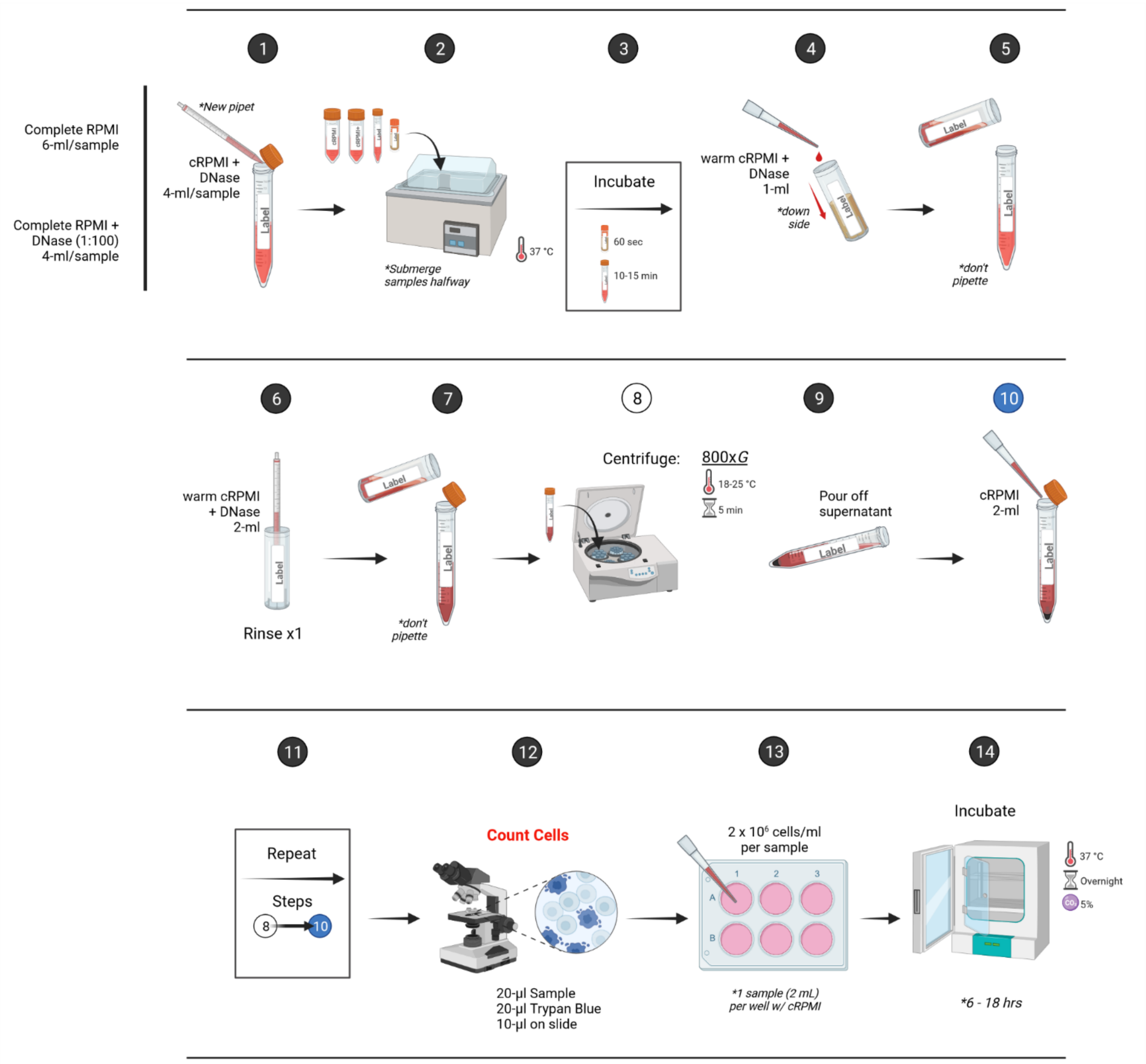

Protocol 5: *Thawing & Preparing PBMC's*

Preparation of cells

1| Determine the total number of PBMCs needed for the experiment, taking into account the number of stimulations to be performed, including a negative and positive control well for each sample. Select cell vials to be thawed accordingly.
2| Obtain 2-ml complete RPMI (culture medium) and 5-ml of Complete RPMI + DNase (1:100) in separate tubes and submerge in a 37 degrees C water bath for a minimum of 15 minutes.
3| Thaw cell sample vials in a 37 degrees C water bath (for 60 seconds) until there is a pea-sized pellet of ice remaining.
4| Add 1-ml of warm cRPMI + DNase down the side of the warmed sample.
5| Transfer the cell suspensions to a correspondingly labeled 15-ml conical tube.
6| Use 2-ml of warm cRPMI + DNase down the empty tube of the sample cryovial to rinse and pour (do not pipette) to the labeled 15-ml conical tube.
7| Vortex the 15-ml conical tube to suspend cells.
8| Centrifuge at 800g for 5 min at room temperature (18–28 degrees C).
9| Decant supernatant.
10| Resuspend cells in 2-ml of culture medium.

Steps 1-10 are for thawing the frozen PBMCs. Steps 11-14 are for recovering and resting the frozen PBMCs for stimulation assays.

11| Repeat centrifugation, decant supernatant and resuspend cells in 2-ml of culture medium.
12| In an Eppendorf, mix 20-µl of sample with 20-µl of Trypan blue (make sure to resuspend by vortexing after mixture).
12a| Add 10µl of the suspended cell mixture on a hemocytometer slide.
12b| Count cells to ensure an adequate number is available for the ICS experiment.
13| Place cells in labeled T-25 culture vials at density of 2 x106 PBMCs per ml
14| Culture overnight (6 – 18 hours) in an incubator at 37 degrees C and 5% CO_2.

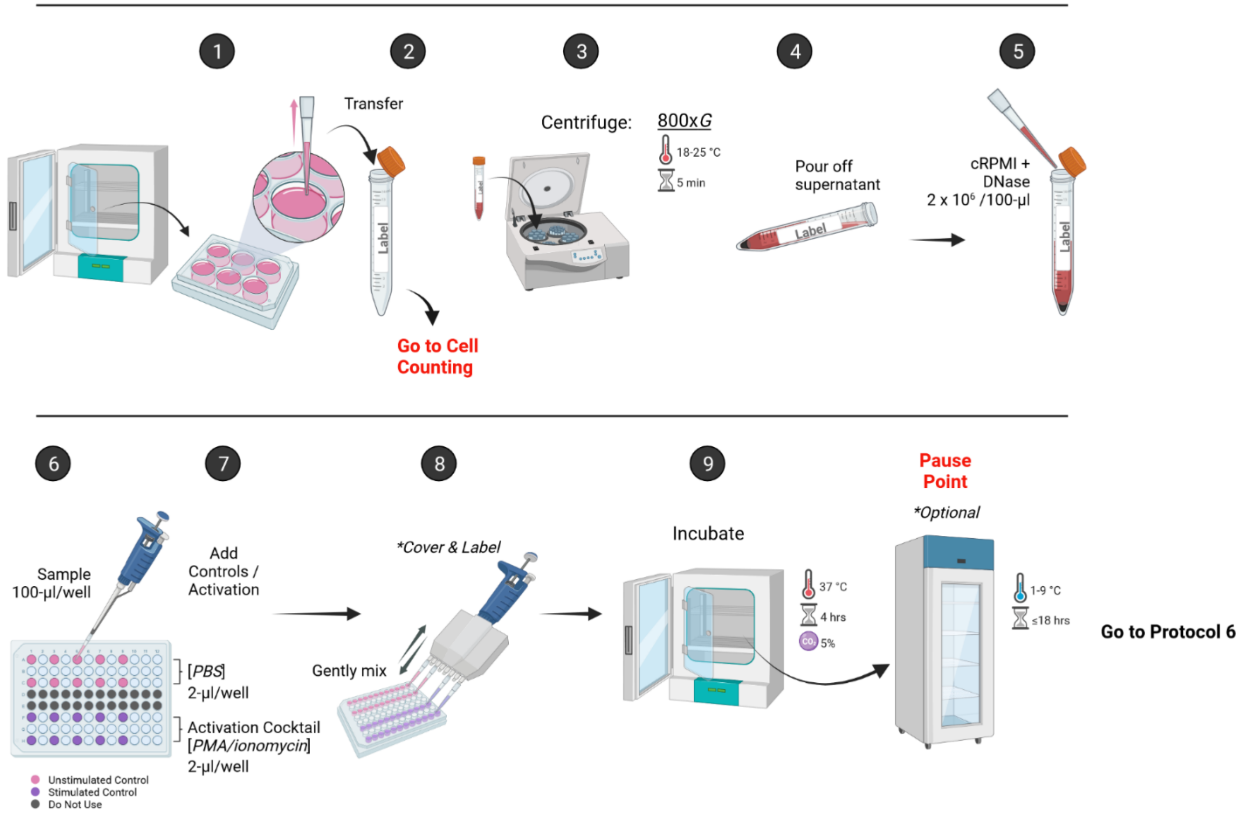

Protocol 5: *PBMC Plate Preparation & Stimulation*

PBMC Plate Preparation and Stimulation

1| Transfer cells from T-25 culture vials to a labeled 15-ml conical tube.
2| Count cells to determine overnight recovery and viability (using same Trypan method as above).
3| Centrifuge tube at 800g for 5 min at room temperature (18–28 degrees C).
4| Decant supernatant.
5| Dilute cells to a density of 2x106 PBMCs per 100-µl in culture medium with DNase.
*Use this equation to determine dilution: (desired concentration / given concentration)*total volume
* Label the plate cover with sample and peptide identification.
6| Pipet 100 ul of cells into their appropriate wells.
7| Pipet 100 ul of stimulation (PMA/ionomycin – diluted to a 1:10 ratio with cRPMI) and negative control (PBS) mixes to the appropriate wells on the plate.
8| Mix cells and peptide negative control and positive control mixes with a multichannel pipette.
9| Incubate for 4 h in an incubator at 37 degrees C and 5% CO2 for 6-18 hours.

'PAUSE POINT After incubation, plates containing cell samples may be stored in the refrigerator at 1–9 degrees C for up to 18 h.

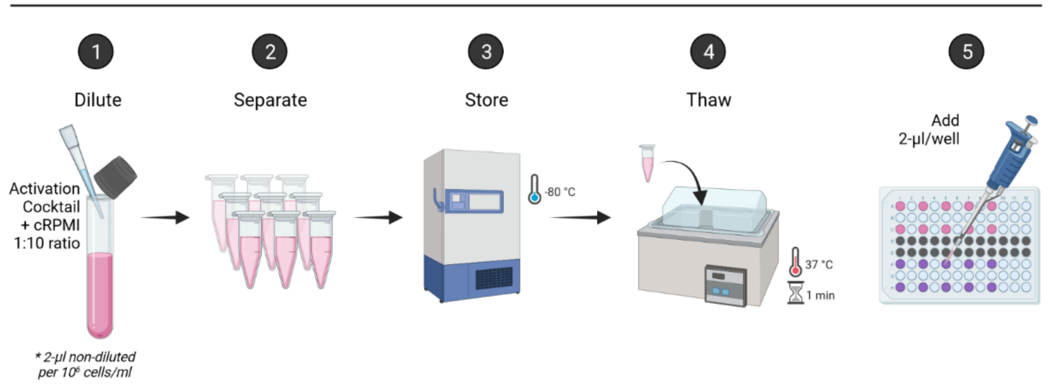

Leukocyte Activation Cocktail

PROTOCOL 6: PBMC Staining

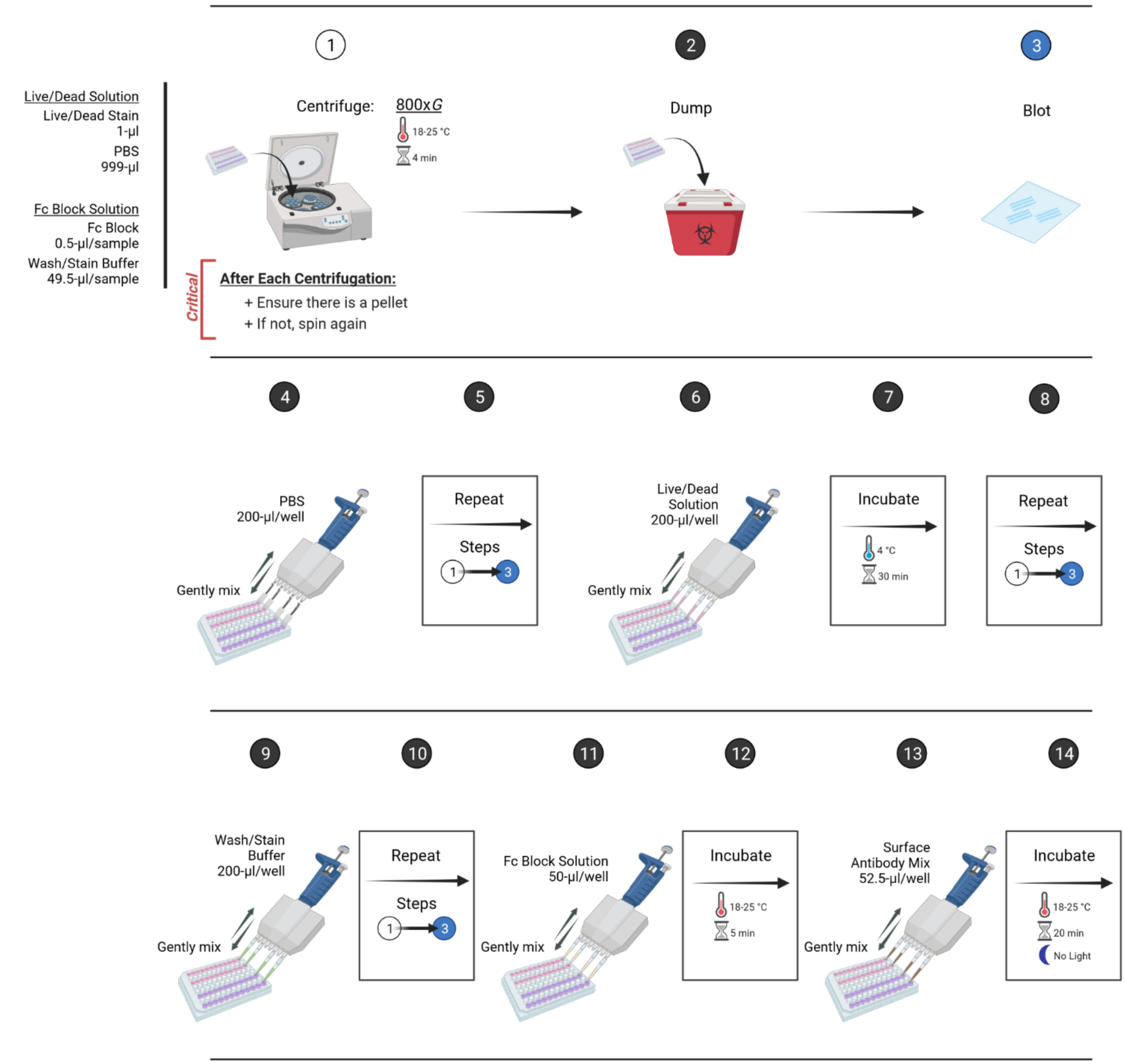

Protocol 6: *PBMC Staining*

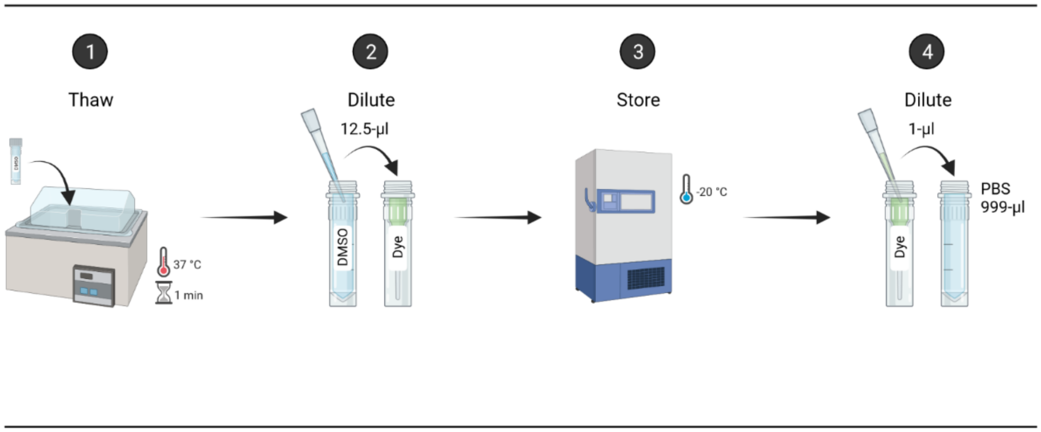

Live/Dead Solution

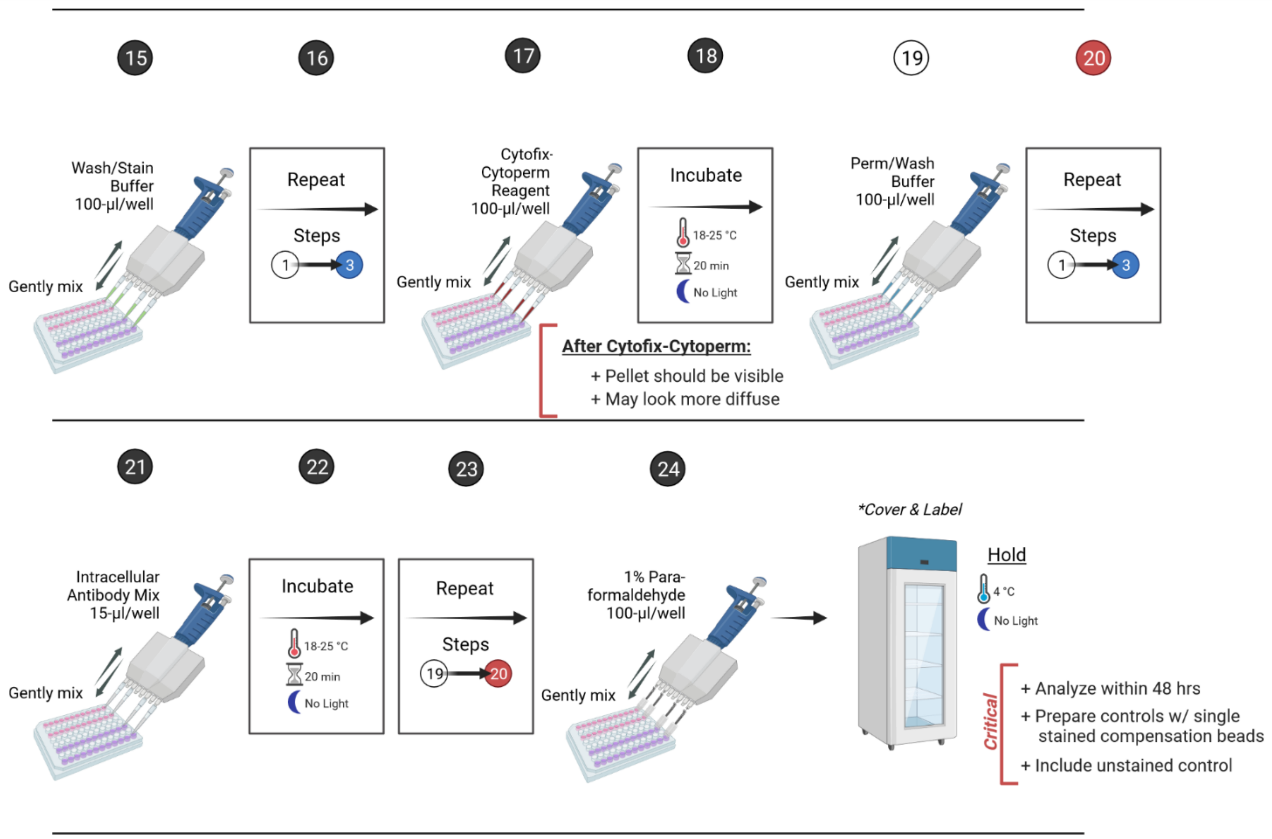

Protocol 6: *PBMC Staining*

After stimulation, remove plates from the incubator.

1| Centrifuge the plate at 800g for 4 min at room temperature.
2| Flick supernatant into biohazard waste container under a laminar flow hood.
3| Immediately and quickly blot plate on a blue underpad.
4| Add 200ul of 1xPBS to each of the wells, mixing well.
5| Repeat steps 1-3
6| Add 200ul of Live/dead stain solution.
7| Incubate 30 min at 4 degrees C.
8| Repeat steps 1-3
9| Add 200ul of wash/stain buffer to each of the wells, mixing well.
10| Repeat steps 1-3
11| Add 50ul Fc Block solution to each sample well, mixing well.
12| Incubate for 5 mins at RT.
13| Add 52.5ul of surface staining mix to each of the wells, mixing well.
14| Incubate for 20 min at room temperature in the dark.
15| Add 100ul of wash/stain buffer to each of the wells.
16| Repeat steps 1-3
17| Add 100ul of Cytofix-Cytoperm reagent to each of the wells, mixing well.
18| Incubate for 20 min at room temperature in the dark.
19| Add 100ul of Perm/Wash buffer to each of the wells.
20| Repeat steps 1-3
21| Add 15ul (5ul per antibody by three antibodies) of intracellular antibody mix to the wells, mixing well.
22| Incubate for 20 min at room temperature in the dark.
23| Repeat steps 19 and 20
24| Resuspend PBMCs in 100 ul of 1% paraformaldehyde. Hold plate(s) at 4 degrees C, protected from light with aluminum foil, until analysis.

* Process samples by flow cytometry within 48 h.

CRITICAL STEP After each centrifugation step, check to ensure there is a visible pellet. If no pellet is seen, centrifuge the plate again. Once the Cytofix-Cytoperm reagent has been added, the pellet may look more diffuse but should still be visible.

| Prepare compensation controls using single-stained compensation beads (such as anti–mouse k-chain beads).

CRITICAL STEP An unstained control must also be included when using compensation beads.

| Analyze sample files using an analysis program such as FlowJo, or Cell Stream analysis software.

Vitamin C HPLC Protocol:

The plasma and buffy coat vitamin C (total ascorbic acid) were measured using Agilent 1220 series high-performance liquid chromatography (HPLC) equipped with G1330B FC/ ALS autosampler thermostat to maintain the sample temperature between 6-8°C throughout the analysis. 10 µl of the plasma/buffy coat samples were injected into an Eclipse Plus C18 (250 mm × 4.6 mm, 5 µm) column (Agilent, Santa Clara, CA) maintained at 25°C for the separation of ascorbates. Two mobile phases, (A) 0.03 mol L-1 phosphoric acid and (B) methanol was used with a flow rate of 0.5 ml/min. A gradient program with, 100–0% B (0–8 min), 0-10% B (3 min), 10-90% B for 4 min, 90-0% B (1min), and remain isocratic for 4 min for the separation of vitamin C. The peak was monitored at 243 nm, both ascorbic acid and dehydroascorbic acid were measured separately to quantify the total vitamin C content and the results were expressed as ascorbic acid equivalents in µg/ml of plasma/buffy coat sample.
