# Supplementary material for: Comparative Effectiveness of Ascorbic Acid vs. Calcium Ascorbate Ingestion on Pharmacokinetic Profiles and Immune Biomarkers in Healthy Adults: A Preliminary Study
Source: Nutrients. 2024 Oct 2;16(19):3358. doi: 10.3390/nu16193358 (PMC11479081; doi:10.3390/nu16193358)
Supplement: Supplementary file 1 [file nutrients-16-03358-s001.zip › Supplemental Tables.pdf]

Table S1a. Baseline demographics for low-dose study.

Variable	Crossover Treatments			p-Value
	Female	Male	Mean	
N size	27	19	46	
Age [years]	27 ± 12	23 ± 4	26 ± 10	0.156
Height [cm]	164.0 ± 6.6	173.7 ± 6.7	168.0 ± 8.2	<0.001
Weight [kg]	60.7 ± 9.2	73.2 ± 11.2	65.9 ± 11.7	<0.001
BMI [kg/m^2]	22.5 ± 2.9	24.2 ± 3.2	23.2 ± 3.1	0.068
Heart Rate [bpm]	73 ± 10	71 ± 10	72 ± 10	0.641
Systolic BP [mmHg]	109 ± 10	117 ± 11	112 ± 11	0.013
Dyastolic BP [mmHg]	71 ± 6	75 ± 8	73 ± 7	0.064

Data are expressed as means ± standard deviations for both treatments A and B. Sex differences were assesed using One-way ANOVA.

Table S1b. Baseline demographics for high-dose study.

Variable	Crossover Treatments			p-Value
	Female	Male	Mean	
N size	24	23	47	
Age [years]	25 ± 11	25 ± 7	25 ± 9	0.853
Height [cm]	164.6 ± 7.9	176.7 ± 6.6	170.5 ± 9.4	<0.001
Weight [kg]	63.0 ± 11.3	77.6 ± 11.4	70.2 ± 13.5	<0.001
BMI [kg/m^2]	23.0 ± 2.6	24.8 ± 3.5	23.9 ± 3.2	0.054
Heart Rate [bpm]	72 ± 8	68 ± 10	70 ± 9	0.137
Systolic BP [mmHg]	111 ± 9	120 ± 13	115 ± 12	0.008
Dyastolic BP [mmHg]	70 ± 7	74 ± 7	72 ± 7	0.036

Data are expressed as means ± standard deviations for both treatments A and B. Sex differences were assesed using One-way ANOVA.

Table S2a. Baseline serum health markers for the low-dose study.

	Crossover Treatments			Sex
	Female	Male	Mean	p-Value
N size	27	19	46	
Total Cholesterol [mg/dL]	171.26 ± 35.95	160.74 ± 24.32	166.91 ± 31.79	0.274
Triglycerides [mg/dL]	75.78 ± 26.35	106.05 ± 45.96	88.28 ± 38.38	0.007
HDL Cholesterol [mg/dL]	62.70 ± 14.63	40.47 ± 9.03	53.52 ± 16.70	<0.001
LDL Cholesterol [mg/dL]	92.04 ± 29.03	99.89 ± 22.98	95.28 ± 26.71	0.331
Non-HDL Cholesterol [mg/dL]	108.56 ± 31.73	120.26 ± 28.75	113.39 ± 30.76	0.207
VLDL Cholesterol [mg/dL]	16.52 ± 3.62	20.37 ± 6.47	18.11 ± 5.29	0.013
LDL/HDL Ratio	1.53 ± 0.59	2.67 ± 1.15	2.00 ± 1.03	<0.001
Total Cholesterol/HDL Ratio	2.81 ± 0.67	4.22 ± 1.42	3.39 ± 1.25	<0.001
Glucose [mg/dL]	89.44 ± 7.81	97.05 ± 10.50	92.59 ± 9.68	0.007
Blood Urea Nitrogen [mg/dL]	12.19 ± 4.05	12.53 ± 3.47	12.33 ± 3.78	0.767
Creatinine [mg/dL]	0.81 ± 0.16	0.94 ± 0.12	0.87 ± 0.16	0.005
Estimated Glomerular Filtration Rate [African American] [ml/min/1.73]	116.52 ± 22.19	128.26 ± 15.57	121.37 ± 20.39	0.053
Estimated Glomerular Filtration Rate [Non-African American] [ml/min/1.73]	100.44 ± 19.11	110.63 ± 13.64	104.65 ± 17.64	0.053
BUN/Creatinine Ratio	15.85 ± 5.08	13.37 ± 3.37	14.83 ± 4.58	0.070
Sodium [meq/L]	139.81 ± 2.56	140.84 ± 2.19	140.24 ± 2.44	0.162
Potassium [meq/L]	4.52 ± 0.94	4.31 ± 0.50	4.43 ± 0.79	0.362
Chloride [meq/L]	103.67 ± 2.77	102.11 ± 2.56	103.02 ± 2.77	0.059
Carbon Dioxide [meq/L]	23.56 ± 1.91	24.95 ± 1.78	24.13 ± 1.96	0.016
Calcium [mg/dL]	9.32 ± 0.42	9.43 ± 0.35	9.37 ± 0.39	0.383
Total Proteins [g/dL]	6.93 ± 0.42	7.18 ± 0.51	7.03 ± 0.47	0.073
Albumin [g/dL]	4.47 ± 0.29	4.69 ± 0.23	4.56 ± 0.29	0.010
Globulin [g/dL]	2.45 ± 0.36	2.49 ± 0.36	2.47 ± 0.36	0.729
Albumin/Globulin Ratio	1.87 ± 0.32	1.93 ± 0.27	1.89 ± 0.30	0.512
Bilirubin [mg/dL]	0.45 ± 0.33	0.67 ± 0.44	0.54 ± 0.39	0.055
Alkaline Phosphatase [U/L]	60.30 ± 8.82	82.84 ± 16.48	69.61 ± 16.72	<0.001
Aspartate Aminotransferase [U/L]	18.11 ± 3.64	21.37 ± 4.27	19.46 ± 4.19	0.008
Alanine Aminotransferase [U/L]	14.11 ± 5.08	22.05 ± 10.12	17.39 ± 8.46	0.001

Data are expressed as means ± standard deviations for both treatments A and B. Sex differences were assesed using One-way ANOVA.

Table S2b. Baseline serum health markers for the high-dose study.

	Crossover Treatments			Sex
	Female	Male	Mean	p-Value
N size	24	23	47	
Total Cholesterol [mg/dL]	185.00 ± 35.39	162.78 ± 25.79	174.13 ± 32.72	0.018
Triglycerides [mg/dL]	93.38 ± 35.97	102.30 ± 48.62	97.74 ± 42.40	0.477
HDL Cholesterol [mg/dL]	60.42 ± 17.38	46.74 ± 11.22	53.72 ± 16.09	0.003
LDL Cholesterol [mg/dL]	105.33 ± 32.41	96.39 ± 23.01	100.96 ± 28.26	0.283
Non-HDL Cholesterol [mg/dL]	124.58 ± 36.38	116.04 ± 27.43	120.40 ± 32.25	0.370
VLDL Cholesterol [mg/dL]	19.25 ± 5.27	19.65 ± 6.31	19.45 ± 5.74	0.813
LDL/HDL Ratio	1.91 ± 0.81	2.20 ± 0.80	2.05 ± 0.81	0.212
Total Cholesterol/HDL Ratio	3.26 ± 0.94	3.66 ± 0.99	3.46 ± 0.98	0.160
Glucose [mg/dL]	91.17 ± 8.30	92.43 ± 8.52	91.79 ± 8.34	0.608
Blood Urea Nitrogen [mg/dL]	13.13 ± 4.35	13.39 ± 4.26	13.26 ± 4.26	0.833
Creatinine [mg/dL]	0.79 ± 0.11	0.91 ± 0.12	0.85 ± 0.13	0.001
Estimated Glomerular Filtration Rate [African American] [ml/min/1.73]	122.00 ± 20.74	132.57 ± 17.24	127.17 ± 19.64	0.065
Estimated Glomerular Filtration Rate [Non-African American] [ml/min/1.73]	105.38 ± 17.84	114.35 ± 14.86	109.77 ± 16.89	0.068
BUN/Creatinine Ratio	16.92 ± 5.95	14.83 ± 5.01	15.89 ± 5.55	0.200
Sodium [meq/L]	139.79 ± 2.13	139.17 ± 3.35	139.49 ± 2.78	0.453
Potassium [meq/L]	4.51 ± 0.58	4.97 ± 1.63	4.73 ± 1.22	0.200
Chloride [meq/L]	103.46 ± 2.48	102.04 ± 1.61	102.77 ± 2.20	0.026
Carbon Dioxide [meq/L]	23.17 ± 2.04	24.96 ± 1.64	24.04 ± 2.04	0.002
Calcium [mg/dL]	9.42 ± 0.22	9.51 ± 0.39	9.47 ± 0.31	0.317
Total Proteins [g/dL]	6.93 ± 0.47	7.07 ± 0.33	7.00 ± 0.41	0.262
Albumin [g/dL]	4.53 ± 0.23	4.69 ± 0.21	4.60 ± 0.23	0.015
Globulin [g/dL]	2.40 ± 0.38	2.38 ± 0.25	2.39 ± 0.32	0.786
Albumin/Globulin Ratio	1.94 ± 0.32	1.99 ± 0.21	1.96 ± 0.27	0.541
Bilirubin [mg/dL]	0.40 ± 0.31	0.71 ± 0.47	0.55 ± 0.43	0.010
Alkaline Phosphatase [U/L]	63.50 ± 19.79	72.30 ± 18.32	67.81 ± 19.39	0.121
Aspartate Aminotransferase [U/L]	19.83 ± 10.47	22.48 ± 6.40	21.13 ± 8.73	0.304
Alanine Aminotransferase [U/L]	15.38 ± 9.30	20.70 ± 10.27	17.98 ± 10.04	0.069

Data are expressed as means ± standard deviations for both treatments A and B. Sex differences were assesed using One-way ANOVA.

Table S3a. Plasma vitamin C concentrations observed in the low-dose study.

			Hour							Mean			
**Variable**	**Treatment**	**N**	**0**	**1**	**2**	**4**	**8**	**24**	**32**	**(SEM)**	**Effect**	**p-Value**	**η_p^2**
AA - Plasma	AA	***46***	1.71 ± 1.29	3.07 ± 1.84 †	4.02 ± 2.24 †	3.80 ± 2.10 †	3.43 ± 1.95 †	2.48 ± 1.47 †	2.40 ± 1.37 †	2.99 ± 0.25	T	<0.001	0.536
[μg/mL]	CA	***46***	1.58 ± 1.37	2.97 ± 2.04 †	3.78 ± 2.19 †	3.65 ± 2.06 †	3.29 ± 2.04 †	2.60 ± 1.57 †	2.55 ± 1.41 †	2.92 ± 0.25	T x T	0.478	0.010
	Time	***92***	1.65 ± 1.32	3.02 ± 1.93 †	3.90 ± 2.21 †	3.72 ± 2.07 †	3.36 ± 1.99 †	2.54 ± 1.51 †	2.48 ± 1.39 †	2.95 ± 0.17			
DHA - Plasma	AA	***46***	1.39 ± 1.74	1.35 ± 0.79	1.66 ± 0.98	1.85 ± 0.95	1.73 ± 0.88	1.34 ± 0.66	1.39 ± 0.71	1.53 ± 0.11	T	0.011	0.052
[μg/mL]	CA	***46***	1.62 ± 1.85	1.47 ± 1.08	1.93 ± 1.30	1.91 ± 1.30	1.84 ± 1.17	1.48 ± 0.87	1.42 ± 0.89	1.67 ± 0.11	T x T	0.779	0.002
	Time	***92***	1.51 ± 1.79	1.41 ± 0.94	1.79 ± 1.15	1.88 ± 1.13	1.78 ± 1.03	1.41 ± 0.77	1.40 ± 0.80	1.60 ± 0.08			
Total Vitamin C - Plasma	AA	***46***	3.10 ± 2.00	4.42 ± 2.25 †	5.68 ± 2.58 †	5.64 ± 2.50 †	5.15 ± 2.32 †	3.83 ± 1.85 †	3.79 ± 1.74 †	4.52 ± 0.28	T	<0.001	0.393
[μg/mL]	CA	***46***	3.21 ± 2.11	4.44 ± 2.51 †	5.71 ± 2.57 †	5.56 ± 2.41 †	5.13 ± 2.45 †	4.08 ± 1.88 †	3.97 ± 1.76 †	4.59 ± 0.28	T x T	0.817	0.002
	Time	***92***	3.15 ± 2.05	4.43 ± 2.37 †	5.69 ± 2.56 †	5.60 ± 2.44 †	5.14 ± 2.37 †	3.95 ± 1.86 †	3.88 ± 1.74 †	4.55 ± 0.20			

Data are expressed as means ± standard deviations for Treatments A and B. Data were analyzed using a multivariate and univariate General Linear Model with repeated measures. P-levels, with partial ETA squared (η_p^2), are listed for univariate within-subject (Greenhouse-Geisser) time (T), and treatment x time (T x T) effects. Multivariate Wilk's Lambda showed significant time (p<0.001, η_p^2 = 0..329), but not treatment x time (p = 0.883, η_p^2 = 0.006), within-subject effects. Pairwise comparisons, with LSD confidence interval adjustment, for simple main effects are indicated by the following superscripts: difference from baseline value, † = p<0.05 (‡ = p>0.05 to p<0.10); between treatments, ∗ = p<0.05 (⁑ = >0.05 to p<0.10). η_p^2 effect size values of 0.01 - 0.05 = small, 0.06 - 0.13 = medium, and >0.14 = large.

Table S3b. Plasma vitamin C concentrations observed in the high-dose study.

			Hour							Mean			
**Variable**	**Treatment**	**N**	**0**	**1**	**2**	**4**	**8**	**24**	**32**	**(SEM)**	**Effect**	**p-Value**	**η_p^2**
AA - Plasma	AA	***47***	2.25 ± 1.68	5.47 ± 3.13 †	7.46 ± 3.87 †	7.46 ± 3.40 †	6.21 ± 2.78 †	2.74 ± 1.27 †	5.18 ± 2.23 †	5.25 ± 0.32	T	<0.001	0.619
[μg/mL]	CA	***47***	2.63 ± 1.83	5.41 ± 2.65 †	7.32 ± 3.21 †	7.10 ± 2.98 †	5.69 ± 2.85 †	2.67 ± 1.35	4.68 ± 2.33 †	5.07 ± 0.32	T x T	0.439	0.010
	Time	***94***	2.44 ± 1.76	5.44 ± 2.88 †	7.39 ± 3.54 †	7.28 ± 3.18 †	5.95 ± 2.81 †	2.71 ± 1.30 †	4.93 ± 2.28 †	5.16 ± 0.23			
DHA - Plasma	AA	***47***	3.11 ± 1.09	3.42 ± 1.32 ‡	3.78 ± 1.23 †	4.05 ± 1.49 †	3.92 ± 1.23 †	2.06 ± 0.67 †	2.78 ± 0.72 ‡	3.30 ± 0.12	T	<0.001	0.477
[μg/mL]	CA	***47***	2.27 ± 0.96	3.35 ± 1.12 †	4.05 ± 1.17 †	4.31 ± 1.54 †	3.94 ± 1.23 †	2.05 ± 0.69 ‡	2.86 ± 1.14 †	3.26 ± 0.12	T x T	<0.001	0.049
	Time	***94***	2.69 ± 1.11 ⁑	3.38 ± 1.22 †	3.92 ± 1.20 †	4.18 ± 1.51 †	3.93 ± 1.22 †	2.06 ± 0.68 †	2.82 ± 0.95	3.28 ± 0.09			
Total Vitamin C - Plasma	AA	***47***	5.36 ± 2.11	8.89 ± 3.79 †	11.24 ± 4.18 †	11.51 ± 3.88 †	10.13 ± 3.12 †	4.81 ± 1.60 †	7.96 ± 2.69 †	8.56 ± 0.40	T	<0.001	0.696
[μg/mL]	CA	***47***	4.90 ± 2.53	8.77 ± 3.36 †	11.37 ± 3.91 †	11.40 ± 3.85 †	9.63 ± 3.46 †	4.72 ± 1.77	7.54 ± 3.06 †	8.33 ± 0.40	T x T	0.815	0.004
	Time	***94***	5.13 ± 2.33	8.83 ± 3.56 †	11.30 ± 4.03 †	11.46 ± 3.85 †	9.88 ± 3.29 †	4.76 ± 1.68 †	7.75 ± 2.87 †	8.44 ± 0.28			

Data are expressed as means ± standard deviations for Treatments A and B. Data were analyzed using a multivariate and univariate General Linear Model with repeated measures. P-levels, with partial ETA squared (η_p^2), are listed for univariate within-subject (Greenhouse-Geisser) time (T), and treatment x time (T x T) effects. Multivariate Wilk's Lambda showed significant time (p<0.001, η_p^2 = 0.447) and treatment x time (p<0.001, η_p^2 = 0.031), within-subject effects. Pairwise comparisons, with LSD confidence interval adjustment, for simple main effects are indicated by the following superscripts: difference from baseline value, † = p<0.05 (‡ = p>0.05 to p<0.10); between treatments, ∗ = p<0.05 (⁑ = >0.05 to p<0.10). η_p^2 effect size values of 0.01 - 0.05 = small, 0.06 - 0.13 = medium, and >0.14 = large. DHA = dehydroAA.

Table S3c. Low dose study plasma vitamin C concentration Area Under the Curve (AUC)

			Hour						Mean				Cumulative
**Variable**	**Treatment**	**N**	**0 - 1**	**1 - 2**	**2 - 4**	**4 - 8**	**8 - 24**	**24 - 32**	**(SEM)**	**Effect**	**p-Value**	**η_p^2**	**(Mean ± SD)**
AA - Plasma	AA	***46***	2.39 ± 1.52	3.54 ± 1.97 †	7.81 ± 4.21 †	14.45 ± 7.94 †	47.29 ± 26.63 †	19.55 ± 11.13 †	15.84 ± 1.32	T	<0.001	0.744	95.03 ± 52.20
AUC [μg-hr/mL]	CA	***46***	2.28 ± 1.64	3.38 ± 2.05 †	7.43 ± 4.19 †	13.87 ± 8.12 †	47.13 ± 28.30 †	20.61 ± 11.76 †	15.78 ± 1.32	T x T	0.795	0.001	94.69 ± 55.15
	Time	***92***	2.33 ± 1.57	3.46 ± 2.00 †	7.62 ± 4.18 †	14.16 ± 7.99 †	47.21 ± 27.32 †	20.08 ± 11.40 †	15.81 ± 0.93				94.86 ± 53.40
DHA - Plasma	AA	***46***	1.37 ± 0.89	1.51 ± 0.82	3.50 ± 1.76 †	7.15 ± 3.40 †	24.57 ± 11.17 †	10.92 ± 5.23 †	8.17 ± 0.64	T	<0.001	0.768	49.02 ± 21.09
AUC [μg-hr/mL]	CA	***46***	1.54 ± 1.11	1.70 ± 1.14	3.84 ± 2.56 †	7.50 ± 4.81 †	26.53 ± 15.53 †	11.58 ± 6.74 †	8.78 ± 0.64	T x T	0.547	0.005	52.70 ± 30.35
	Time	***92***	1.46 ± 1.00	1.60 ± 0.99	3.67 ± 2.19 †	7.32 ± 4.15 †	25.55 ± 13.49 †	11.25 ± 6.01 †	8.48 ± 0.45				50.86 ± 26.05
Total Vitamin C - Plasma	AA	***46***	3.76 ± 1.69	5.05 ± 2.35 †	11.32 ± 5.00 †	21.59 ± 9.30 †	71.85 ± 32.26 †	30.47 ± 14.10 †	24.01 ± 1.59	T	<0.001	0.825	144.05 ± 63.21
AUC [μg-hr/mL]	CA	***46***	3.82 ± 1.93	5.08 ± 2.44 †	11.27 ± 4.91 †	21.38 ± 9.60 †	73.66 ± 34.23 †	32.19 ± 14.35 †	24.57 ± 1.59	T x T	0.722	0.002	147.40 ± 66.33
	Time	***92***	3.79 ± 1.81	5.06 ± 2.38 †	11.30 ± 4.93 †	21.48 ± 9.40 †	72.76 ± 33.09 †	31.33 ± 14.17 †	24.29 ± 1.13				145.72 ± 64.45

Data are expressed as means ± standard deviations for Treatments A and B. Data were analyzed using a multivariate and univariate General Linear Model with repeated measures. P-levels, with partial ETA squared (η_p^2), are listed for univariate within-subject (Greenhouse-Geisser) time (T), and treatment x time (T x T) effects. Multivariate Wilk's Lambda showed significant time (p<0.001, η_p^2 = 0.597), but not treatment x time (p = 0.990, η_p^2 = 0.003), within-subject effects. Pairwise comparisons, with LSD confidence interval adjustment, for simple main effects are indicated by the following superscripts: difference from baseline value, † = p<0.05 (‡ = p>0.05 to p<0.10); between treatments, ∗ = p<0.05 (⁑ = >0.05 to p<0.10). η_p^2 effect size values of 0.01 - 0.05 = small, 0.06 - 0.13 = medium, and >0.14 = large. Treatment differences in Cumulative AUC values were assessed using One-way ANOVA. DHA = dehydroAA.

Table S3d. High dose study plasma vitamin C concentration Area Under the Curve (AUC)

			Hour						Mean				Cumulative
**Variable**	**Treatment**	**N**	**0 - 1**	**1 - 2**	**2 - 4**	**4 - 8**	**8 - 24**	**24 - 32**	**(SEM)**	**Effect**	**p-Value**	**η_p^2**	**(Mean ± SD)**
AA - Plasma	AA	***47***	3.86 ± 2.27	6.47 ± 3.22 †	14.92 ± 7.05 †	27.34 ± 11.85 †	71.63 ± 30.12 †	31.68 ± 13.16 †	25.98 ± 1.55	T	0.331	0.012	155.89 ± 63.92
AUC [μg-hr/mL]	CA	***47***	4.02 ± 2.16	6.37 ± 2.81 †	14.41 ± 5.97 †	25.58 ± 11.02 †	66.90 ± 31.75 †	29.39 ± 13.64 †	24.44 ± 1.55	T x T	0.293	0.013	146.66 ± 63.58
	Time	***94***	3.94 ± 2.21	6.42 ± 3.01 †	14.67 ± 6.51 †	26.46 ± 11.41 †	69.26 ± 30.87 †	30.53 ± 13.38 †	25.21 ± 1.10				151.28 ± 63.58
DHA - Plasma	AA	***47***	3.26 ± 1.04	3.60 ± 1.17 †	7.83 ± 2.57 †	15.94 ± 5.04 †	47.87 ± 12.67 †	19.38 ± 5.04 †	16.31 ± 0.60	T	<0.001	0.279	97.89 ± 24.74
AUC [μg-hr/mL]	CA	***47***	2.81 ± 0.93	3.70 ± 1.06 †	8.36 ± 2.56 †	16.48 ± 5.17 †	47.91 ± 12.93 †	19.64 ± 6.28 †	16.48 ± 0.60	T x T	<0.001	0.053	98.91 ± 24.62
	Time	***94***	3.04 ± 1.00 *	3.65 ± 1.12 †	8.10 ± 2.56 †	16.21 ± 5.09 †	47.89 ± 12.73 †	19.51 ± 5.66 †	16.40 ± 0.42				98.40 ± 24.55
Total Vitamin C - Plasma	AA	***47***	7.12 ± 2.75	10.06 ± 3.68 †	22.75 ± 7.80 †	43.29 ± 13.31 †	119.49 ± 35.87 †	51.06 ± 16.41 †	42.30 ± 1.91	T	<0.001	0.133	253.78 ± 75.93
AUC [μg-hr/mL]	CA	***47***	6.83 ± 2.84	10.07 ± 3.49 †	22.77 ± 7.51 †	42.07 ± 13.75 †	114.80 ± 40.21 †	49.03 ± 17.87 †	40.93 ± 1.91	T x T	0.052	0.029	245.57 ± 80.92
	Time	***94***	6.98 ± 2.78	10.07 ± 3.57 †	22.76 ± 7.61 †	42.68 ± 13.47 †	117.15 ± 37.97 †	50.04 ± 17.09 †	41.61 ± 1.35				249.68 ± 78.15

Data are expressed as means ± standard deviations for Treatments A and B. Data were analyzed using a multivariate and univariate General Linear Model with repeated measures. P-levels, with partial ETA squared (η_p^2), are listed for univariate within-subject (Greenhouse-Geisser) time (T), and treatment x time (T x T) effects. Multivariate Wilk's Lambda showed significant time (p<0.001, η_p^2 = 0.727), but not treatment x time (p = 0.930, η_p^2 = 0.005), within-subject effects. Pairwise comparisons, with LSD confidence interval adjustment, for simple main effects are indicated by the following superscripts: difference from baseline value, † = p<0.05 (‡ = p>0.05 to p<0.10); between treatments, ∗ = p<0.05 (⁑ = >0.05 to p<0.10). η_p^2 effect size values of 0.01 - 0.05 = small, 0.06 - 0.13 = medium, and >0.14 = large. Treatment differences in Cumulative AUC values were assessed using One-way ANOVA. DHA = dehydroAA.

Table S4a. Leukocyte AA, DHA, and total vitamin C levels for the low dose study determined from buffy coat samples.

Variable	Treatment	N	Hour 0	1	2	4	8	24	32	Mean (SEM)	Effect	p-Value	η_p^2
AA - Buffy Coat	AA	***45***	3.84 ± 9.84	7.08 ± 17.19	3.01 ± 8.46	3.91 ± 9.13	5.47 ± 10.98	6.19 ± 15.07	2.46 ± 8.51	4.57 ± 1.07	T	0.147	0.019
[μg/mL]	CA	***45***	4.36 ± 8.25	5.96 ± 11.51	3.72 ± 6.98	4.15 ± 7.95	3.71 ± 8.35	6.19 ± 13.28	5.45 ± 10.85	4.79 ± 1.07	T x T	0.618	0.008
	Time	***90***	4.10 ± 9.03	6.52 ± 14.56	3.36 ± 7.72	4.03 ± 8.51	4.59 ± 9.74	6.19 ± 14.12	3.96 ± 9.81	4.68 ± 0.76			
DHA - Buffy Coat	AA	***45***	2.24 ± 4.84	3.27 ± 7.11 ‡	3.29 ± 8.29	3.54 ± 8.01	2.42 ± 4.37	2.85 ± 7.01	2.06 ± 4.78	2.81 ± 0.64	T	0.529	0.009
[μg/mL]	CA	***45***	3.12 ± 5.38	4.17 ± 6.48	3.60 ± 5.36	3.37 ± 5.44	2.80 ± 5.10	4.38 ± 6.47	4.05 ± 6.03	3.64 ± 0.64	T x T	0.698	0.006
	Time	***90***	2.68 ± 5.10	3.72 ± 6.78	3.44 ± 6.95	3.45 ± 6.81	2.61 ± 4.73	3.62 ± 6.75	3.06 ± 5.50	3.23 ± 0.45			
Total Vitamin C - Buffy Coat	AA	***45***	6.08 ± 11.61	10.35 ± 21.93	6.30 ± 13.34	7.45 ± 14.80	7.89 ± 13.94	9.04 ± 17.99	4.52 ± 10.74	7.37 ± 1.55	T	0.151	0.019
[μg/mL]	CA	***45***	7.48 ± 11.22	10.13 ± 15.75	7.31 ± 10.62	7.51 ± 11.77	6.51 ± 12.21	10.57 ± 17.19	9.51 ± 14.39	8.43 ± 1.55	T x T	0.553	0.009
	Time	***90***	6.78 ± 11.38	10.24 ± 18.99	6.81 ± 12.00	7.48 ± 13.29	7.20 ± 13.05	9.80 ± 17.51	7.02 ± 12.87 ⁑	7.90 ± 1.10			

Data are expressed as means ± standard deviations for Treatments A and B. Data were analyzed using a multivariate and univariate General Linear Model with repeated measures. P-levels, with partial ETA squared (η_p^2), are listed for univariate within-subject (Greenhouse-Geisser) time (T), and treatment x time (T x T) effects. Multivariate Wilk's Lambda showed no significant time ($p = 0.295$, $\eta_p^2 = 0.013$) or treatment x time ($p = 0.870$, $\eta_p^2 = 0.006$), within-subject effects. Pairwise comparisons, with LSD confidence interval adjustment, for simple main effects are indicated by the following superscripts: difference from baseline value, † = p<0.05 (‡ = p>0.05 to p<0.10); between treatments, ∗ = p<0.05 (⁑ = >0.05 to p<0.10). η_p^2 effect size values of 0.01 - 0.05 = small, 0.06 - 0.13 = medium, and >0.14 = large. DHA = dehydroAA.

Table S4b. Leukocyte AA, DHA, and total vitamin C levels for the high dose study determined from buffy coat samples.

Variable	Treatment	N	Hour 0	1	2	4	8	24	32	Mean (SEM)	Effect	p-Value	η_p^2
AA - Buffy Coat	AA	***47***	1.51 ± 3.99	0.54 ± 2.22 †	0.71 ± 2.63 †	0.97 ± 3.86	0.77 ± 2.66 †	1.57 ± 5.27	0.97 ± 3.08 †	1.01 ± 0.28	T	0.331	0.012
[μg/mL]	CA	***46***	0.06 ± 0.18	0.08 ± 0.30	0.11 ± 0.25	0.12 ± 0.37	0.26 ± 0.44	0.16 ± 0.52	0.13 ± 0.26	0.13 ± 0.28	T x T	0.293	0.013
	Time	***93***	0.79 ± 2.91 ∗	0.31 ± 1.60 †	0.42 ± 1.89	0.55 ± 2.77	0.52 ± 1.92	0.87 ± 3.81 ⁑	0.55 ± 2.23 ⁑	0.57 ± 0.20 ∗			
DHA - Buffy Coat	AA	***47***	1.56 ± 1.17	1.17 ± 1.31 †	1.48 ± 1.84	1.34 ± 1.15	1.63 ± 2.11	3.52 ± 2.13 †	1.68 ± 1.27	1.77 ± 0.14	T	<0.001	0.279
[μg/mL]	CA	***46***	1.91 ± 0.98	1.14 ± 0.76 †	1.13 ± 0.83 †	1.04 ± 0.82 †	1.52 ± 0.90 †	2.34 ± 0.95 ‡	1.36 ± 0.85 †	1.49 ± 0.14	T x T	<0.001	0.053
	Time	***93***	1.73 ± 1.09	1.15 ± 1.07 †	1.31 ± 1.44 †	1.19 ± 1.01 †	1.58 ± 1.62	2.94 ± 1.75 †∗	1.52 ± 1.09	1.63 ± 0.10			
Total Vitamin C - Buffy Coat	AA	***47***	3.07 ± 5.05	1.71 ± 3.02 †	2.20 ± 3.64 †	2.31 ± 4.57	2.40 ± 3.56 †	5.09 ± 6.79 †	2.65 ± 3.66	2.77 ± 0.39	T	<0.001	0.897
[μg/mL]	CA	***46***	1.96 ± 1.09	1.22 ± 0.85 †	1.24 ± 0.94 †	1.16 ± 1.00	1.78 ± 1.09	2.50 ± 1.18	1.49 ± 0.96	1.62 ± 0.40	T x T	0.052	0.029
	Time	***93***	2.52 ± 3.69	1.46 ± 2.23 †	1.72 ± 2.70 †⁑	1.74 ± 3.36 †⁑	2.10 ± 2.65 †	3.81 ± 5.04 †∗	2.08 ± 2.73 ‡∗	2.20 ± 0.28 ∗			

Data are expressed as means ± standard deviations for Treatments A and B. Data were analyzed using a multivariate and univariate General Linear Model with repeated measures. P-levels, with partial ETA squared (η_p^2), are listed for univariate within-subject (Greenhouse-Geisser) time (T), and treatment x time (T x T) effects. Multivariate Wilk's Lambda showed significant time ($p<0.001$, $\eta_p^2 = 0.156$) and treatment x time ($p<0.001$, $\eta_p^2 = 0.034$), within-subject effects. Pairwise comparisons, with LSD confidence interval adjustment, for simple main effects are indicated by the following superscripts: difference from baseline value, † = p<0.05 (‡ = p>0.05 to p<0.10); between treatments, ∗ = p<0.05 (⁑ = >0.05 to p<0.10). η_p^2 effect size values of 0.01 - 0.05 = small, 0.06 - 0.13 = medium, and >0.14 = large. DHA = dehydroAA.

Table S4c. Leukocyte AA, DHA, and total vitamin C levels for the low dose study were determined from buffy coat samples.

Variable	Treatment	N	Hour 0 - 1	1 - 2	2 - 4	4 - 8	8 - 24	24 - 32	Mean (SEM)	Effect	p-Value	η_p^2	Cumulative (Mean ± SD)
AA - Buffy Coat	AA	***45***	5.46 ± 10.98	5.04 ± 10.02	6.92 ± 15.87	18.75 ± 34.42 †	93.26 ± 184.19 †	34.60 ± 72.43 †	27.34 ± 6.80	T	<0.001	0.191	164.04 ± 289.98
AUC [μg-hr/mL]	CA	***45***	5.16 ± 9.32	4.84 ± 8.74	7.87 ± 13.83	15.72 ± 28.27 †	79.20 ± 143.04 †	46.56 ± 83.22 †	26.56 ± 6.80	T x T	0.601	0.004	159.34 ± 256.31
	Time	***90***	5.31 ± 10.13	4.94 ± 9.35	7.39 ± 14.81	17.24 ± 31.35 †	86.23 ± 164.13 †	40.58 ± 77.80 †	26.95 ± 4.81				161.69 ± 272.13
DHA - Buffy Coat	AA	***45***	2.75 ± 4.98	3.28 ± 6.73	6.83 ± 15.73 †	11.91 ± 20.04 †	42.13 ± 77.01 †	19.65 ± 36.40 †	14.43 ± 3.54	T	<0.001	0.241	86.55 ± 130.32
AUC [μg-hr/mL]	CA	***45***	3.65 ± 5.49	3.88 ± 5.53	6.96 ± 10.03 †	12.34 ± 19.20 †	57.49 ± 82.76 †	33.75 ± 46.12 †	19.68 ± 3.54	T x T	0.304	0.013	118.07 ± 153.34
	Time	***90***	3.20 ± 5.23	3.58 ± 6.13	6.90 ± 13.12 †	12.12 ± 19.51 †	49.81 ± 79.86 †	26.70 ± 41.91 †	17.05 ± 2.50				102.31 ± 142.38
Total Vitamin C - Buffy Coat	AA	***45***	8.21 ± 14.67	8.32 ± 14.39	13.75 ± 26.94 ‡	30.66 ± 47.50 †	135.39 ± 222.25 †	54.25 ± 90.62 †	41.76 ± 9.11	T	<0.001	0.263	250.59 ± 362.24
AUC [μg-hr/mL]	CA	***45***	8.81 ± 12.98	8.72 ± 12.49	14.83 ± 20.89 †	28.06 ± 44.48 †	136.69 ± 201.59 †	80.31 ± 113.96 †	46.24 ± 9.11	T x T	0.605	0.004	277.41 ± 371.03
	Time	***90***	8.51 ± 13.77	8.52 ± 13.40	14.29 ± 23.97 †	29.36 ± 45.77 †	136.04 ± 210.98 †	67.28 ± 103.21 †	44.00 ± 6.44				264.00 ± 364.85

Data are expressed as means ± standard deviations for Treatments A and B. Data were analyzed using a multivariate and univariate General Linear Model with repeated measures. P-levels, with partial ETA squared (η_p^2), are listed for univariate within-subject (Greenhouse-Geisser) time (T), and treatment x time (T x T) effects. Multivariate Wilk's Lambda showed significant time ($p<0.001$, $\eta_p^2 = 0.155$), but not treatment x time ($p = 0.529$, $\eta_p^2 = 0.010$), within-subject effects. Pairwise comparisons, with LSD confidence interval adjustment, for simple main effects are indicated by the following superscripts: difference from baseline value, † = p<0.05 (‡ = p>0.05 to p<0.10); between treatments, ∗ = p<0.05 (⁑ = >0.05 to p<0.10). η_p^2 effect size values of 0.01 - 0.05 = small, 0.06 - 0.13 = medium, and >0.14 = large. Treatment differences in Cumulative AUC values were assessed using One-way ANOVA. DHA = dehydroAA.

Table S4d. Leukocyte AA, DHA, and total vitamin C area under the curve values for the high dose study determined from buffy coat samples.

Variable	Treatment	N	Hour 0 - 1	1 - 2	2 - 4	4 - 8	8 - 24	24 - 32	Mean (SEM)	Effect	p-Value	η_p^2	Cumulative (Mean ± SD)
AA - Buffy Coat	AA	***47***	1.03 ± 2.84	0.63 ± 1.82 †	1.68 ± 6.40	3.48 ± 11.66 †	18.77 ± 54.12 †	10.17 ± 29.26 †	5.96 ± 1.75	T	0.007	0.072	35.75 ± 101.00
AUC [μg-hr/mL]	CA	***46***	0.07 ± 0.18	0.10 ± 0.21	0.23 ± 0.49	0.75 ± 1.16	3.32 ± 6.36	1.15 ± 2.21	0.93 ± 1.77	T x T	0.055	0.038	5.61 ± 8.95
	Time	***93***	0.55 ± 2.07 ∗	0.36 ± 1.32 ⁑	0.96 ± 4.60	2.13 ± 8.40 †	11.13 ± 39.30 †∗	5.71 ± 21.24 †∗	3.45 ± 1.25 ∗				20.84 ± 73.28 ∗
DHA - Buffy Coat	AA	***47***	1.36 ± 1.18	1.32 ± 1.44	2.82 ± 2.44 †	5.95 ± 5.90 †	41.18 ± 30.99 †	20.78 ± 11.81 †	12.24 ± 0.92	T	<0.001	0.697	73.42 ± 49.90
AUC [μg-hr/mL]	CA	***46***	1.52 ± 0.66	1.13 ± 0.65 †	2.16 ± 1.36 †	5.12 ± 2.84 †	30.91 ± 11.93 †	14.82 ± 4.86 †	9.28 ± 0.93	T x T	0.025	0.051	55.67 ± 18.23
	Time	***93***	1.44 ± 0.96	1.23 ± 1.12 †	2.50 ± 2.00 †	5.54 ± 4.64 †	36.10 ± 24.01 †∗	17.83 ± 9.50 †∗	10.76 ± 0.65 ∗				64.64 ± 38.57 ∗
Total Vitamin C - Buffy Coat	AA	***47***	2.39 ± 3.85	1.95 ± 3.00 †	4.50 ± 7.90 †	9.42 ± 15.49 †	59.95 ± 78.18 †	30.96 ± 37.79 †	18.20 ± 2.47	T	<0.001	0.412	109.18 ± 141.50
AUC [μg-hr/mL]	CA	***46***	1.59 ± 0.71	1.23 ± 0.74 †	2.40 ± 1.57	5.87 ± 3.30 †	34.23 ± 14.52 †	15.96 ± 5.89 †	10.21 ± 2.50	T x T	0.024	0.053	61.28 ± 21.97
	Time	***93***	1.99 ± 2.80	1.59 ± 2.21 †	3.46 ± 5.79 †∗	7.67 ± 11.33 †	47.23 ± 57.68 †∗	23.54 ± 28.07 †∗	14.20 ± 1.76 ∗				85.49 ± 104.05 ∗

Data are expressed as means ± standard deviations for Treatments A and B. Data were analyzed using a multivariate and univariate General Linear Model with repeated measures. P-levels, with partial ETA squared (η_p^2), are listed for univariate within-subject (Greenhouse-Geisser) time (T), and treatment x time (T x T) effects. Multivariate Wilk's Lambda showed significant time ($p<0.001$, $\eta_p^2 = 0.484$) and treatment x time ($p = 0.003$, $\eta_p^2 = 0.029$), within-subject effects. Pairwise comparisons, with LSD confidence interval adjustment, for simple main effects are indicated by the following superscripts: difference from baseline value, † = p<0.05 (‡ = p>0.05 to p<0.10); between treatments, ∗ = p<0.05 (⁑ = >0.05 to p<0.10). η_p^2 effect size values of 0.01 - 0.05 = small, 0.06 - 0.13 = medium, and >0.14 = large. Treatment differences in Cumulative AUC values were assessed using One-way ANOVA. DHA = dehydroAA.

Table S5a. Whole blood markers in response to low-dose supplementation.

Variable	Treatment	N	Hour 0	1	2	4	8	24	32	Mean (SEM)	Effect	p-Value	η_p^2
White Blood Cells	AA	*46*	6.35 ± 1.76	6.07 ± 1.71 †	6.23 ± 1.88	6.32 ± 1.81	6.77 ± 1.82 †	5.83 ± 1.55 †	6.44 ± 1.58	6.29 ± 0.22	T	<0.001	0.148
[K/UL]	CA	*46*	6.13 ± 1.50	5.85 ± 1.42 †	6.19 ± 1.54	5.99 ± 1.40	6.58 ± 1.53 †	5.79 ± 1.47 †	6.78 ± 1.63 †	6.19 ± 0.22	T x T	0.115	0.020
	Time	*92*	6.24 ± 1.63	5.96 ± 1.57 †	6.21 ± 1.71	6.15 ± 1.62	6.68 ± 1.68 †	5.81 ± 1.50 †	6.61 ± 1.61 †	6.24 ± 0.15			
Red Blood Cells (RBC's)	AA	*46*	4.59 ± 0.46	4.58 ± 0.45	4.57 ± 0.45	4.61 ± 0.47	4.56 ± 0.50	4.52 ± 0.47 †	4.43 ± 0.49 †	4.55 ± 0.07	T	<0.001	0.130
[M/UL]	CA	*46*	4.62 ± 0.53	4.61 ± 0.50	4.63 ± 0.53	4.59 ± 0.53	4.60 ± 0.56	4.57 ± 0.53 ‡	4.49 ± 0.52 †	4.59 ± 0.07	T x T	0.471	0.010
	Time	*92*	4.61 ± 0.49	4.60 ± 0.47	4.60 ± 0.49	4.60 ± 0.50	4.58 ± 0.53	4.54 ± 0.50 †	4.46 ± 0.50 †	4.57 ± 0.05			
Hemoglobin	AA	*46*	13.62 ± 1.32	13.60 ± 1.32	13.57 ± 1.40	13.66 ± 1.42	13.58 ± 1.46	13.44 ± 1.41 ‡	13.22 ± 1.48 †	13.52 ± 0.21	T	<0.001	0.111
[G/DL]	CA	*46*	13.68 ± 1.57	13.72 ± 1.55	13.73 ± 1.58	13.63 ± 1.64	13.70 ± 1.61	13.60 ± 1.62	13.32 ± 1.51 †	13.63 ± 0.21	T x T	0.690	0.007
	Time	*92*	13.65 ± 1.44	13.66 ± 1.43	13.65 ± 1.49	13.64 ± 1.52	13.64 ± 1.53	13.52 ± 1.51 ‡	13.27 ± 1.48 †	13.57 ± 0.15			
Hematocrit	AA	*46*	39.97 ± 3.55	39.87 ± 3.45	39.76 ± 3.60	40.08 ± 3.78	39.60 ± 3.97	39.33 ± 3.64 †	38.83 ± 3.86 †	39.63 ± 0.56	T	<0.001	0.088
[%]	CA	*46*	40.23 ± 4.18	40.30 ± 4.16	40.30 ± 4.15	39.95 ± 4.24	40.10 ± 4.37	39.83 ± 4.20	39.25 ± 4.03 †	39.99 ± 0.56	T x T	0.540	0.009
	Time	*92*	40.10 ± 3.86	40.08 ± 3.81	40.03 ± 3.88	40.02 ± 4.00	39.85 ± 4.16	39.58 ± 3.91 †	39.04 ± 3.93 †	39.81 ± 0.39			
Mean Corpuscular Volume	AA	*46*	87.20 ± 4.18	87.23 ± 4.01	87.22 ± 4.09	87.12 ± 4.10	87.04 ± 4.05	87.19 ± 4.21	87.83 ± 4.10 †	87.26 ± 0.58	T	0.071	0.023
[fL]	CA	*46*	87.23 ± 3.84	87.45 ± 3.87	87.30 ± 3.89	87.26 ± 4.31	87.33 ± 4.03	87.36 ± 4.12	87.51 ± 4.12	87.35 ± 0.58	T x T	0.593	0.008
	Time	*92*	87.22 ± 3.99	87.34 ± 3.92	87.26 ± 3.97	87.19 ± 4.18	87.19 ± 4.02	87.28 ± 4.15	87.67 ± 4.09 †	87.31 ± 0.41			
Mean Corpuscular Hemoglobin	AA	*46*	29.71 ± 1.68	29.74 ± 1.58	29.74 ± 1.62	29.66 ± 1.59	29.82 ± 1.61	29.78 ± 1.77	29.87 ± 1.77 ‡	29.76 ± 0.23	T	0.483	0.010
[PG]	CA	*46*	29.64 ± 1.50	29.79 ± 1.69	29.73 ± 1.66	29.73 ± 1.83	29.83 ± 1.74 ‡	29.79 ± 1.56	29.72 ± 1.74	29.75 ± 0.23	T x T	0.835	0.005
	Time	*92*	29.67 ± 1.58	29.77 ± 1.63	29.74 ± 1.63	29.70 ± 1.71	29.82 ± 1.67 †	29.78 ± 1.66	29.80 ± 1.75 ‡	29.75 ± 0.17			
Mean Corpuscular Hemoglobin	AA	*46*	34.05 ± 0.74	34.08 ± 0.71	34.10 ± 0.78	34.04 ± 0.73	34.26 ± 0.83 †	34.14 ± 0.92	34.00 ± 0.87	34.10 ± 0.10	T	0.056	0.023
Concentration [G/DL]	CA	*46*	33.96 ± 0.72	34.04 ± 0.78	34.04 ± 0.82	34.08 ± 0.93	34.14 ± 0.84 ‡	34.11 ± 0.77	33.91 ± 0.89	34.04 ± 0.10	T x T	0.967	0.002
	Time	*92*	34.01 ± 0.73	34.06 ± 0.74	34.07 ± 0.80	34.06 ± 0.83	34.20 ± 0.83 †	34.12 ± 0.84	33.96 ± 0.87	34.07 ± 0.07			
RBC Distribution Width	AA	*46*	12.57 ± 0.85	12.54 ± 0.88	12.57 ± 0.89	12.59 ± 0.91	12.57 ± 0.94	12.54 ± 1.00	12.57 ± 0.87	12.57 ± 0.12	T	0.281	0.014
[%]	CA	*46*	12.56 ± 0.80	12.52 ± 0.82	12.52 ± 0.79	12.47 ± 0.69 †	12.58 ± 0.83	12.51 ± 0.79	12.54 ± 0.81	12.53 ± 0.12	T x T	0.232	0.015
	Time	*92*	12.57 ± 0.82	12.53 ± 0.85 †	12.55 ± 0.84	12.53 ± 0.81	12.58 ± 0.88	12.53 ± 0.90	12.56 ± 0.83	12.55 ± 0.09			
Neutrophils	AA	*46*	49.29 ± 9.02	55.16 ± 9.64 †	56.40 ± 10.38 †	56.91 ± 9.52 †	56.73 ± 8.89 †	49.93 ± 8.70	55.97 ± 7.88 †	54.34 ± 1.11	T	<0.001	0.294
[%]	CA	*46*	49.11 ± 8.67	54.71 ± 8.77 †	56.11 ± 9.03 †	56.67 ± 8.17 †	56.63 ± 7.91 †	50.91 ± 8.95 †	57.85 ± 9.04 †	54.57 ± 1.11	T x T	0.619	0.007
	Time	*92*	49.20 ± 8.80	54.93 ± 9.17 †	56.25 ± 9.67 †	56.79 ± 8.82 †	56.68 ± 8.36 †	50.42 ± 8.79 †	56.91 ± 8.49 †	54.45 ± 0.79			
Lymphocytes	AA	*46*	38.67 ± 8.38	32.82 ± 8.48 †	32.41 ± 9.04 †	32.91 ± 8.54 †	32.96 ± 7.82 †	38.22 ± 8.07	33.89 ± 7.93 †	34.55 ± 1.05	T	<0.001	0.273
[%]	CA	*46*	38.86 ± 8.04	33.53 ± 7.94 †	32.92 ± 8.11 †	33.11 ± 7.81 †	33.01 ± 7.13 †	37.83 ± 8.94	32.24 ± 8.54 †	34.50 ± 1.05	T x T	0.572	0.008
	Time	*92*	38.77 ± 8.17	33.18 ± 8.18 †	32.67 ± 8.54 †	33.01 ± 8.14 †	32.98 ± 7.44 †	38.03 ± 8.47	33.07 ± 8.23 †	34.53 ± 0.74			
Monocytes	AA	*46*	8.00 ± 1.44	8.19 ± 1.87	7.71 ± 1.74	7.18 ± 1.92 †	7.40 ± 2.19 †	8.08 ± 2.01	7.23 ± 2.08 †	7.68 ± 0.23	T	<0.001	0.163
[%]	CA	*46*	8.31 ± 1.81	8.03 ± 1.72 ‡	7.65 ± 1.75 †	7.23 ± 1.63 †	7.48 ± 1.70 †	7.97 ± 1.64 ‡	7.29 ± 1.59 †	7.71 ± 0.23	T x T	0.660	0.007
	Time	*92*	8.15 ± 1.64	8.11 ± 1.79	7.68 ± 1.74 †	7.20 ± 1.77 †	7.44 ± 1.95 †	8.02 ± 1.83	7.26 ± 1.84 †	7.70 ± 0.17			
Eosinophils	AA	*46*	3.05 ± 1.95	2.86 ± 1.91	2.50 ± 1.83 †	2.10 ± 1.61 †	1.94 ± 1.63 †	2.84 ± 2.01 ‡	1.95 ± 1.41 †	2.46 ± 0.27	T	0.011	0.064
[%]	CA	*46*	2.88 ± 2.09	3.87 ± 8.63	2.33 ± 1.60 †	1.97 ± 1.32 †	1.70 ± 1.18 †	2.53 ± 1.76 †	1.72 ± 1.26 †	2.43 ± 0.27	T x T	0.344	0.011
	Time	*92*	2.97 ± 2.02	3.36 ± 6.24	2.41 ± 1.71 †	2.04 ± 1.47 †	1.82 ± 1.42 †	2.69 ± 1.89 †	1.84 ± 1.33 †	2.45 ± 0.19			
Basophils	AA	*46*	0.66 ± 0.26	0.65 ± 0.25	0.68 ± 0.40	0.64 ± 0.27	0.64 ± 0.36	0.68 ± 0.27	0.58 ± 0.23 †	0.65 ± 0.04	T	0.001	0.047
[%]	CA	*46*	0.71 ± 0.30	0.72 ± 0.30	0.67 ± 0.27	0.68 ± 0.30	0.65 ± 0.26	0.68 ± 0.31	0.57 ± 0.24 †	0.67 ± 0.04	T x T	0.595	0.008
	Time	*92*	0.68 ± 0.28	0.68 ± 0.28	0.67 ± 0.34	0.66 ± 0.28	0.65 ± 0.32	0.68 ± 0.29	0.58 ± 0.23 †	0.66 ± 0.03			
Platelets	AA	*46*	263.09 ± 53.16	259.46 ± 51.71	261.36 ± 53.95	265.10 ± 52.30	268.00 ± 52.86	263.48 ± 53.72	268.11 ± 52.37 ‡	264.08 ± 7.57	T	<0.001	0.057
[K/UL]	CA	*46*	261.89 ± 54.62	256.28 ± 52.70 †	257.47 ± 52.18 ‡	260.90 ± 48.40	267.63 ± 56.61 ‡	258.55 ± 51.91	261.39 ± 53.19	260.59 ± 7.57	T x T	0.722	0.006
	Time	*92*	262.49 ± 53.60	257.87 ± 51.94 †	259.41 ± 52.82 ‡	263.00 ± 50.16	267.82 ± 54.47 †	261.02 ± 52.59	264.75 ± 52.60	262.34 ± 5.35			

Data are expressed as means ± standard deviations for Treatments A and B. Data were analyzed using a multivariate and univariate General Linear Model with repeated measures. P-levels, with partial ETA squared (η_p^2), are listed for univariate within-subject (Greenhouse-Geisser) time (T), and treatment x time (T x T) effects. Multivariate Wilk's Lambda showed significant time ($p<0.001$, $\eta_p^2 = 0.160$), but not treatment x time ($p = 0.885$, $\eta_p^2 = 0.021$), within-subject effects. Pairwise comparisons, with LSD confidence interval adjustment, for simple main effects are indicated by the following superscripts: difference from baseline value, † = p<0.05 (‡ = p>0.05 to p<0.10); between Treatments, ∗ = p<0.05 (⁑ = >0.05 to p<0.10). η_p^2 effect size values of 0.01 - 0.05 = small, 0.06 - 0.13 = medium, and >0.14 = large.

Table S5b. Whole blood markers in response to high-dose supplementation.

Variable	Treatment	N	Hour 0	1	2	4	8	24	32	Mean (SEM)	Effect	p-Value	η_p^2
White Blood Cells	AA	*47*	6.14 ± 1.42	5.72 ± 1.29 †	5.90 ± 1.30 †	5.91 ± 1.23 ‡	6.59 ± 1.39 †	5.66 ± 1.20 †	6.57 ± 1.30 †	6.07 ± 0.17	T	<0.001	0.184
[K/UL]	CA	*47*	6.21 ± 1.42	6.01 ± 1.37 †	6.20 ± 1.37	6.04 ± 1.34	6.58 ± 1.48 †	5.76 ± 1.09 †	6.50 ± 1.24 ‡	6.18 ± 0.17	T x T	0.445	0.010
	Time	*94*	6.17 ± 1.41	5.86 ± 1.33 †	6.05 ± 1.33	5.98 ± 1.28 †	6.58 ± 1.43 †	5.71 ± 1.14 †	6.53 ± 1.26 †	6.13 ± 0.12			
Red Blood Cells (RBC's)	AA	*47*	4.66 ± 0.48	4.68 ± 0.52	4.71 ± 0.53 ‡	4.72 ± 0.54 ‡	4.69 ± 0.52	4.62 ± 0.52	4.60 ± 0.50 †	4.67 ± 0.07	T	<0.001	0.117
[M/UL]	CA	*47*	4.70 ± 0.45	4.73 ± 0.44	4.79 ± 0.49 †	4.77 ± 0.50 †	4.70 ± 0.51	4.69 ± 0.45	4.62 ± 0.45 †	4.71 ± 0.07	T x T	0.444	0.010
	Time	*94*	4.68 ± 0.46	4.70 ± 0.48	4.75 ± 0.51 †	4.75 ± 0.52 †	4.70 ± 0.51	4.65 ± 0.49	4.61 ± 0.47 †	4.69 ± 0.05			
Hemoglobin	AA	*47*	13.62 ± 1.45	13.61 ± 1.56	13.72 ± 1.56	13.74 ± 1.64	13.64 ± 1.64	13.52 ± 1.58	13.40 ± 1.56 †	13.61 ± 0.21	T	<0.001	0.131
[G/DL]	CA	*47*	13.72 ± 1.37	13.81 ± 1.45	13.96 ± 1.47 †	13.93 ± 1.49 †	13.75 ± 1.52	13.69 ± 1.35	13.46 ± 1.41 †	13.76 ± 0.21	T x T	0.594	0.008
	Time	*94*	13.67 ± 1.40	13.71 ± 1.50	13.84 ± 1.51 †	13.84 ± 1.56 †	13.69 ± 1.57	13.60 ± 1.46	13.43 ± 1.48 †	13.68 ± 0.15			
Hematocrit	AA	*47*	40.19 ± 3.95	40.38 ± 4.22	40.66 ± 4.27 †	40.68 ± 4.44 ‡	40.30 ± 4.24	40.00 ± 4.27	39.78 ± 4.33	40.28 ± 0.57	T	<0.001	0.104
[%]	CA	*47*	40.40 ± 3.67	40.84 ± 3.71 †	41.28 ± 3.86 †	41.15 ± 3.94 †	40.54 ± 4.12	40.34 ± 3.70	39.99 ± 3.85	40.65 ± 0.57	T x T	0.836	0.005
	Time	*94*	40.29 ± 3.79	40.61 ± 3.96 †	40.97 ± 4.06 †	40.92 ± 4.18 †	40.42 ± 4.16	40.17 ± 3.98	39.89 ± 4.08 †	40.47 ± 0.40			
Mean Corpuscular Volume	AA	*47*	86.33 ± 3.67	86.45 ± 3.91	86.49 ± 3.82	86.37 ± 3.85	86.00 ± 3.97	86.68 ± 3.32	86.51 ± 4.13	86.40 ± 0.51	T	0.283	0.014
[fL]	CA	*47*	86.13 ± 3.42	86.46 ± 3.42 ‡	86.31 ± 3.21	86.34 ± 3.63	86.41 ± 3.66	86.16 ± 3.72	86.72 ± 3.62 †	86.36 ± 0.51	T x T	0.210	0.016
	Time	*94*	86.23 ± 3.53	86.46 ± 3.65 ‡	86.40 ± 3.51	86.35 ± 3.72	86.21 ± 3.80	86.42 ± 3.52	86.62 ± 3.86 †	86.38 ± 0.36			
Mean Corpuscular Hemoglobin	AA	*47*	29.26 ± 1.75	29.13 ± 1.84	29.17 ± 1.73	29.17 ± 1.82	29.07 ± 1.72	29.27 ± 1.44	29.13 ± 1.77	29.17 ± 0.23	T	0.857	0.004
[PG]	CA	*47*	29.24 ± 1.66	29.22 ± 1.61	29.18 ± 1.59	29.21 ± 1.64	29.29 ± 1.60	29.23 ± 1.52	29.18 ± 1.48	29.22 ± 0.23	T x T	0.777	0.005
	Time	*94*	29.25 ± 1.69	29.18 ± 1.72	29.18 ± 1.66	29.19 ± 1.72	29.18 ± 1.66	29.25 ± 1.47	29.16 ± 1.62	29.20 ± 0.16			
Mean Corpuscular Hemoglobin	AA	*47*	33.89 ± 0.99	33.68 ± 1.09 ‡	33.70 ± 0.97 ‡	33.75 ± 1.03	33.80 ± 1.02	33.77 ± 0.94	33.65 ± 0.90 ‡	33.75 ± 0.12	T	0.034	0.025
Concentration [G/DL]	CA	*47*	33.94 ± 1.09	33.79 ± 0.96	33.79 ± 0.96	33.82 ± 0.91	33.89 ± 0.94	33.92 ± 0.91	33.64 ± 0.90 †	33.83 ± 0.12	T x T	0.968	0.002
	Time	*94*	33.92 ± 1.03	33.74 ± 1.03 †	33.75 ± 0.96 †	33.79 ± 0.97	33.84 ± 0.98	33.85 ± 0.92	33.64 ± 0.89 †	33.79 ± 0.08			
RBC Distribution Width	AA	*47*	12.75 ± 0.68	12.73 ± 0.69	12.73 ± 0.71	12.73 ± 0.68	12.79 ± 0.78	12.66 ± 0.65 ‡	12.74 ± 0.74	12.73 ± 0.10	T	0.210	0.016
[%]	CA	*47*	12.76 ± 0.69	12.76 ± 0.68	12.71 ± 0.70	12.70 ± 0.68 †	12.79 ± 0.71	12.75 ± 0.74	12.76 ± 0.68	12.75 ± 0.10	T x T	0.601	0.007
	Time	*94*	12.75 ± 0.68	12.74 ± 0.68	12.72 ± 0.70	12.71 ± 0.68 ‡	12.79 ± 0.74	12.71 ± 0.69	12.75 ± 0.70	12.74 ± 0.07			
Neutrophils	AA	*47*	47.85 ± 7.81	52.67 ± 8.71 †	53.94 ± 9.12 †	52.85 ± 8.46 †	55.01 ± 7.78 †	49.33 ± 7.25 ‡	56.37 ± 8.96 †	52.57 ± 1.02	T	<0.001	0.313
[%]	CA	*47*	47.54 ± 8.76	52.89 ± 8.73 †	55.11 ± 8.93 †	55.77 ± 6.94 †	54.77 ± 7.22 †	48.86 ± 7.85 ‡	54.16 ± 7.73 †	52.73 ± 1.02	T x T	0.028	0.030
	Time	*94*	47.69 ± 8.26	52.78 ± 8.68 †	54.53 ± 9.00 †	54.31 ± 7.83 †⁑	54.89 ± 7.47 †	49.09 ± 7.52 †	55.26 ± 8.39 †	52.65 ± 0.72			
Lymphocytes	AA	*47*	39.61 ± 7.84	34.80 ± 7.94 †	33.95 ± 8.06 †	35.79 ± 7.89 †	33.94 ± 7.22 †	38.89 ± 7.33	33.08 ± 7.79 †	35.72 ± 0.99	T	<0.001	0.292
[%]	CA	*47*	39.81 ± 8.96	34.57 ± 8.03 †	33.10 ± 7.72 †	33.34 ± 5.96 †	34.40 ± 6.68 †	38.92 ± 8.47	35.09 ± 7.45 †	35.60 ± 0.99	T x T	0.030	0.029
	Time	*94*	39.71 ± 8.37	34.69 ± 7.94 †	33.53 ± 7.86 †	34.57 ± 7.06 †⁑	34.17 ± 6.92 †	38.91 ± 7.88	34.09 ± 7.65 †	35.66 ± 0.70			
Monocytes	AA	*47*	7.87 ± 1.95	8.04 ± 2.13	7.85 ± 1.91	7.44 ± 1.96 †	7.46 ± 1.88 †	7.75 ± 1.93	7.32 ± 2.16 †	7.68 ± 0.26	T	<0.001	0.122
[%]	CA	*47*	7.97 ± 1.93	7.97 ± 1.81	7.56 ± 1.86 †	7.10 ± 1.76 †	7.34 ± 1.59 †	8.03 ± 1.82	7.45 ± 2.25 †	7.63 ± 0.26	T x T	0.108	0.020
	Time	*94*	7.92 ± 1.93	8.00 ± 1.97	7.71 ± 1.88 ‡	7.27 ± 1.86 †	7.40 ± 1.73 †	7.89 ± 1.87	7.39 ± 2.19 †	7.65 ± 0.18			
Eosinophils	AA	*47*	3.60 ± 2.53	3.49 ± 2.68	3.23 ± 2.77 †	2.99 ± 2.81 †	2.69 ± 2.65 †	3.40 ± 2.56	2.31 ± 2.09 †	3.10 ± 0.39	T	<0.001	0.338
[%]	CA	*47*	3.70 ± 3.15	3.58 ± 3.18	3.34 ± 3.29 †	2.87 ± 2.86 †	2.54 ± 2.55 †	3.35 ± 2.57 †	2.41 ± 2.06 †	3.11 ± 0.39	T x T	0.613	0.007
	Time	*94*	3.65 ± 2.84	3.54 ± 2.92	3.29 ± 3.03 †	2.93 ± 2.82 †	2.61 ± 2.59 †	3.37 ± 2.55 †	2.36 ± 2.07 †	3.11 ± 0.27			
Basophils	AA	*47*	0.67 ± 0.30	0.68 ± 0.32	0.67 ± 0.34	0.63 ± 0.30	0.59 ± 0.34 †	0.63 ± 0.32	0.60 ± 0.26 ‡	0.64 ± 0.04	T	0.002	0.040
[%]	CA	*47*	0.63 ± 0.26	0.68 ± 0.32	0.63 ± 0.22	0.61 ± 0.25	0.57 ± 0.26 ‡	0.63 ± 0.28	0.61 ± 0.30	0.62 ± 0.04	T x T	0.897	0.004
	Time	*94*	0.65 ± 0.28	0.68 ± 0.32	0.65 ± 0.29	0.62 ± 0.27	0.58 ± 0.30 †	0.63 ± 0.30	0.60 ± 0.28 ‡	0.63 ± 0.03			
Platelets	AA	*47*	266.09 ± 75.53	255.89 ± 59.03 †	262.30 ± 62.11	264.90 ± 64.49	270.77 ± 63.67	259.96 ± 66.93	269.02 ± 62.79	264.13 ± 8.94	T	0.002	0.042
[K/UL]	CA	*47*	261.62 ± 61.71	260.76 ± 59.69	262.26 ± 60.77	265.04 ± 63.95	264.96 ± 62.10	260.15 ± 60.21	264.79 ± 60.62	262.79 ± 8.94	T x T	0.331	0.012
	Time	*94*	263.85 ± 68.63	258.32 ± 59.09 ‡	262.28 ± 61.11	264.97 ± 63.88	267.86 ± 62.62	260.05 ± 63.32	266.90 ± 61.42	263.46 ± 6.32			

Data are expressed as means ± standard deviations for Treatments A and B. Data were analyzed using a multivariate and univariate General Linear Model with repeated measures. P-levels, with partial ETA squared (η_p^2), are listed for univariate within-subject (Greenhouse-Geisser) time (T), and treatment x time (T x T) effects. Multivariate Wilk's Lambda showed significant time ($p<0.001$, $\eta_p^2 = 0.183$), but not treatment x time ($p = 0.564$, $\eta_p^2 = 0.024$), within-subject effects. Pairwise comparisons, with LSD confidence interval adjustment, for simple main effects are indicated by the following superscripts: difference from baseline value, † = p<0.05 (‡ = p>0.05 to p<0.10); between Treatments, ∗ = p<0.05 (⁑ = >0.05 to p<0.10). η_p^2 effect size values of 0.01 - 0.05 = small, 0.06 - 0.13 = medium, and >0.14 = large.

Table S6. Dose and body weight adjusted plasma AA, DHA, and total vitamin C pharmacokinetic analysis from plasma values.

	Variable	Treatment	N	Weight kg	Dosage µg	Dosage µg/kg	Cmax µg/mL	N	Tmax hr	AUC (0-t) µg-hr/mL	AUC∞ (area) µg-hr/mL	AUC∞ (expo) µg-hr/mL	AUMC∞ (area) µg-hr*hr/mL	AUMC∞ (expo) µg-hr*hr/mL	MRT (area) hr	MRT (expo) hr	Vd (obs area) mL	Vd (area) mL	Vd (area) /kg mL/kg	Vd (expo) mL
Low Dose	AA	AA	*45*	65.87 ± 11.83	250,000 ± 0	3,915.34 ± 696.85	4.24 ± 2.16	*46*	2	94.06 ± 52.37	124.1 ± 564.6	180.7 ± 667.7	103,084 ± 331,037	140,951 ± 402,449	11.3 ± 197.5	35.3 ± 220.5	87 ± 593,776	119,682 ± 597,034	2,017 ± 10,055	113,860 ± 322,874
		CA	*44*	65.49 ± 11.24	250,000 ± 0	3,929.09 ± 678.02	4.12 ± 2.29	*46*	2	95.26 ± 56.34	-253.7 ± 3,258.6	-193.7 ± 3,271.6	2,973,459 ± 19,072,411	3,006,268 ± 19,068,591	-121.1 ± 931.7	-93.5 ± 939.4	-225,384 ± 2,391,365	120,375 ± 348,385	1,972 ± 5,939	120,806 ± 444,085
		p-Value		0.876	0	0.925	0.808	χ^2	0.164	0.918	0.446	0.454	0.316	0.316	0.354	0.373	0.541	0.995	0.979	0.933
	DHA	AA	*42*	65.81 ± 12.10	250,000 ± 0	3,923.83 ± 711.61	2.31 ± 0.78	*43*	4	51.07 ± 16.96	79.1 ± 380.1	83.5 ± 381.3	114,248 ± 434,264	114,502 ± 434,197	57.6 ± 308.7	57.3 ± 303.6	272,655 ± 1,801,120	19,568 ± 550,866	410 ± 8,941	226,403 ± 667,923
		CA	*42*	65.20 ± 11.41	250,000 ± 0	3,949.44 ± 686.94	2.60 ± 1.27	*43*	2	56.50 ± 28.63	33.0 ± 430.0	11.8 ± 486.6	58,126 ± 225,665	75,628 ± 249,522	104.2 ± 548.6	25.6 ± 234.8	149,419 ± 918,497	-566,460 ± 4,589,950	-6,142 ± 55,117	20,177 ± 440,239
		p-Value		0.812	0	0.867	0.218	χ^2	0.203	0.293	0.604	0.454	0.459	0.616	0.632	0.594	0.694	0.414	0.449	0.099
	Total Vitamin C	AA	*45*	65.87 ± 11.83	250,000 ± 0	3,915.34 ± 696.85	6.59 ± 2.07	*46*	2	142.05 ± 62.44	1,618.5 ± 8,616.8	1,621.8 ± 8,616.0	28,449,534 ± 188,226,056	28,450,799 ± 188,225,808	531.8 ± 3,284.0	547.3 ± 3,281.3	1,414,080 ± 9,109,026	1,636 ± 1,243,826	-499 ± 20,681	152,555 ± 620,172
		CA	*44*	65.49 ± 11.24	250,000 ± 0	3,929.09 ± 678.02	6.47 ± 2.38	*46*	2	150.91 ± 64.86	-161.5 ± 5,688.7	-179.7 ± 5,689.8	6,849,990 ± 33,396,879	6,857,261 ± 33,395,490	1.1 ± 1,272.9	-9.4 ± 1,271.9	126,262 ± 2,121,013	160,280 ± 598,082	2,374 ± 8,583	96,289 ± 175,790
		p-Value		0.876	0	0.925	0.799	χ^2	0.770	0.513	0.254	0.249	0.455	0.456	0.320	0.296	0.363	0.447	0.396	0.564
High Dose	AA	AA	*45*	69.39 ± 13.14	500,000 ± 0	7,469.12 ± 1,458.11	8.54 ± 3.41	*47*	2	159.77 ± 61.28	99.5 ± 51.9	164.9 ± 377.2	1,680 ± 2,741	31,146 ± 114,662	17.0 ± 21.0	15.1 ± 70.2	-14,258 ± 120,789	-84,611 ± 134,784	-1,286 ± 2,044	-4,305 ± 140,558
		CA	*44*	69.38 ± 13.00	500,000 ± 0	7,466.60 ± 1,455.50	8.11 ± 3.15	*47*	2	147.16 ± 59.90	102.8 ± 188.2	122.2 ± 261.9	13,981 ± 79,124	19,677 ± 79,403	13.3 ± 84.9	6.4 ± 80.0	-33,783 ± 328,756	-33,687 ± 458,704	-720 ± 6,692	-81,510 ± 547,842
		p-Value		0.997	0	0.994	0.544	χ^2	0.853	0.329	0.910	0.537	0.300	0.585	0.779	0.586	0.710	0.477	0.589	0.363
	DHA	AA	*45*	69.74 ± 13.63	500,000 ± 0	7,445.48 ± 1,485.78	4.75 ± 1.66	*47*	8, 24	100.29 ± 32.93	201.9 ± 956.5	236.7 ± 1,627.7	399,536 ± 2,582,055	907,314 ± 3,879,576	141.7 ± 536.7	85.6 ± 569.7	196,511 ± 1,827,211	-817,606 ± 3,430,645	-12,836 ± 56,597	-119,353 ± 726,474
		CA	*45*	69.96 ± 13.42	500,000 ± 0	7,417.15 ± 1,476.61	4.77 ± 1.44	*47*	8	98.22 ± 24.88	-40.6 ± 540.8	-77.6 ± 559.1	85,793 ± 517,863	93,086 ± 517,446	275.6 ± 2,375.0	-47.8 ± 167.7	-227,578 ± 719,252	-2,571,884 ± 20,346,346	-42,629 ± 333,308	619,648 ± 4,171,257
		p-Value		0.941	0	0.928	0.962	χ^2	0.436	0.738	0.142	0.224	0.426	0.166	0.713	0.136	0.151	0.570	0.556	0.245
	Total Vitamin C	AA	*46*	69.95 ± 13.55	500,000 ± 0	7,420.69 ± 1,478.77	12.34 ± 3.96	*47*	2	254.56 ± 76.58	159.5 ± 347.3	106.8 ± 2,205.4	26,164 ± 121,326	446,485 ± 1,855,553	12.0 ± 77.5	-18.5 ± 199.6	-30,735 ± 197,234	-51,816 ± 73,695	-749 ± 1,029	46,869 ± 270,616
		CA	*45*	69.96 ± 13.42	500,000 ± 0	7,417.15 ± 1,476.61	12.30 ± 4.10	*47*	2	242.47 ± 80.42	93.4 ± 148.8	126.2 ± 377.8	5,570 ± 17,160	21,668 ± 51,878	8.2 ± 39.9	-4.9 ± 50.9	-47,132 ± 58,229	-81,559 ± 81,078	-1,177 ± 1,090	-48,130 ± 104,237
		p-Value		0.999	0	0.991	0.963	χ^2	0.674	0.464	0.243	0.954	0.263	0.128	0.770	0.659	0.594	0.070	0.058	0.030

Data are expressed as means ± standard deviations for the Ester-C and AA treatments. Tmax values are presented as time points with the highest frequency counts and significance shown with chi-squared (χ^2). Univariate and χ^2 p-values are listed under each variable. Differences between treatments are indicated by the following superscripts: ⁎ = p<0.05 and ⁑ = p>0.05 to p<0.10. Partial Eta squared effect sizes (ηp2) are reported as indicators of magnitude of effect where 0.01 was considered a small effect, 0.06 was considered a medium effect, and 0.14 was administered dose to be eliminated). MRT (expo) = MRT calculated from exponential terms. Vd (obs area) = Based only on observed data. Use when final slope tends to overestimate. AUC∞ Vd (area) = Vd based on AUC. Widely used value, but reflects Vd only during elimination phase. Vd (area) / kg = Above value divided by body weight in kg. Vd (expo) = Vd calculated using exponential terms. Vss (area) = Steady state calculation based on trapezoid calculation of curve areas. Vss (expo) = Steady state calculation based on exponential terms. CL (obs area) = S kinetics. D/A Phase Intercept = Distribution/Absorption phase intercept. For oral doses, the sign of the last coefficient (absorption phase) should be negative. D/A Phase Slope = Distribution/Absorption Phase Slope. Obtained by linear regression analysis of selected region on graph. D/A Phase Rate = Distribution/Absorption Phase Rate. Rate = 2.303 x slope. D/A Phase Half-life = Time or concentration to distribute/absorp by one-half. Remains constant for 1st order kinetics.

Vss (area) mL	Vss (expo) mL	CL (obs area) mL/hr	CL (area) mL/hr	CL (area) /kg mL/hr/kg	CL (expo) mL/hr	E Phase Intercept µg/mL	E Phase Slope 1/hr	E Phase Rate 1/hr	E Phase Half-life hr	DA Phase Intercept µg/mL	DA Phase Slope 1/hr	DA Phase Rate 1/hr	DA Phase Halflife hr	A Phase Intercept µg/mL	A Phase Slope 1/hr	A Phase Rate 1/hr	A Phase Halflife hr	Effect	p-Level	η_p^2
574,716 ± 1,960,326	-221,727 ± 2,771,736	4,260 ± 4,055	1,089 ± 15,308	11.9 ± 255.8	1,474 ± 14,163	12.49 ± 58.41	-0.004 ± 0.024	0.008 ± 0.054	3.9 ± 132.6	12.51 ± 63.28	-0.036 ± 0.062	0.084 ± 0.144	19.19 ± 73.95	-27.34 ± 122.19	-0.255 ± 0.454	0.587 ± 1.046	1.40 ± 3.88	Treatment	0.881	0.279
281,424 ± 407,913	683,845 ± 2,593,867	6,111 ± 11,605	3,057 ± 10,283	46.0 ± 151.8	1,275 ± 18,048	4.31 ± 7.37	0.001 ± 0.017	-0.001 ± 0.040	-82.9 ± 645.1	3.27 ± 7.01	-0.032 ± 0.086	0.074 ± 0.197	18.57 ± 88.13	-13.61 ± 25.86	-0.270 ± 0.754	0.621 ± 1.737	-0.42 ± 7.34			
0.334	0.115	0.316	0.479	0.448	0.954	0.359	0.350	0.344	0.379	0.339	0.780	0.782	0.971	0.468	0.910	0.910	0.146			
920,151 ± 2,383,579	2,467,951 ± 12,953,254	5,490 ± 2,020	4,657 ± 16,952	68.9 ± 275.0	-4,091 ± 26,332	2.91 ± 5.42	0.001 ± 0.022	-0.003 ± 0.051	31.2 ± 213.0	3.30 ± 5.16	-0.023 ± 0.107	0.052 ± 0.246	6.69 ± 13.67	-5.84 ± 10.04	-0.154 ± 0.286	0.355 ± 0.658	1.78 ± 5.03	Treatment	0.487	0.386
75,431,879 ± 482,939,270	377,182 ± 1,744,235	5,254 ± 2,054	22,295 ± 144,177	262.0 ± 1,738.7	1,002 ± 20,201	3.98 ± 5.15	-0.003 ± 0.022	0.008 ± 0.051	14.8 ± 120.4	3.66 ± 4.69	-0.024 ± 0.059	0.056 ± 0.136	-5.11 ± 111.78	-8.41 ± 9.69	-0.188 ± 0.545	0.432 ± 1.255	7.99 ± 29.39			
0.320	0.303	0.595	0.433	0.479	0.323	0.354	0.344	0.339	0.666	0.743	0.929	0.933	0.499	0.236	0.728	0.727	0.181			
1,735,114 ± 7,180,499	2,826,903 ± 15,874,786	2,606 ± 2,702	1,011 ± 32,898	29.5 ± 528.9	-2,891 ± 20,274	4.56 ± 3.89	0.000 ± 0.010	0.000 ± 0.023	370.0 ± 2275.2	4.30 ± 4.07	-0.037 ± 0.123	0.086 ± 0.284	7.52 ± 16.77	-12.37 ± 15.83	-0.375 ± 0.411	0.865 ± 0.948	1.81 ± 5.98	Treatment	0.669	0.330
1,333,805 ± 5,393,470	228,188 ± 406,728	2,395 ± 2,459	-2,384 ± 17,656	-34.1 ± 253.3	-778 ± 5,096	6.41 ± 9.97	-0.002 ± 0.013	0.004 ± 0.029	2.9 ± 879.7	4.55 ± 9.94	-0.024 ± 0.085	0.056 ± 0.195	-9.89 ± 53.07	-16.26 ± 26.20	-0.351 ± 0.488	0.808 ± 1.123	0.48 ± 3.77			
0.767	0.281	0.702	0.547	0.473	0.504	0.250	0.484	0.474	0.320	0.875	0.561	0.562	0.039	0.397	0.799	0.799	0.213			
157,934 ± 385,736	615,245 ± 3,352,217	3,874 ± 2,748	6,864 ± 4,712	100.9 ± 69.1	1,678 ± 8,759	0.73 ± 1.04	0.038 ± 0.025	-0.087 ± 0.057	-5.5 ± 14.0	9.17 ± 6.14	-0.020 ± 0.038	0.047 ± 0.087	15.78 ± 49.50	-100.42 ± 369.04	-0.448 ± 0.688	1.031 ± 1.586	0.22 ± 3.61	Treatment	0.974	0.231
623,249 ± 2,320,472	700,427 ± 3,683,583	4,213 ± 2,460	6,197 ± 13,569	97.0 ± 207.2	6,625 ± 21,208	0.88 ± 1.24	0.034 ± 0.025	-0.079 ± 0.058	-3.1 ± 51.0	16.22 ± 38.25	-0.046 ± 0.082	0.106 ± 0.188	7.50 ± 20.63	-21.68 ± 44.51	-0.309 ± 0.482	0.711 ± 1.109	0.04 ± 2.34			
0.188	0.909	0.541	0.756	0.906	0.152	0.534	0.498	0.500	0.759	0.225	0.058	0.058	0.308	0.163	0.274	0.274	0.778			
40,453,392 ± 248,165,286	1,941,704 ± 7,002,796	5,500 ± 1,876	31,661 ± 109,113	494.0 ± 1,806.8	3,409 ± 23,565	1.26 ± 1.45	0.019 ± 0.019	-0.044 ± 0.043	34.6 ± 290.0	110.63 ± 714.91	-0.029 ± 0.128	0.068 ± 0.296	22.02 ± 269.86	-112.19 ± 714.54	-0.193 ± 0.465	0.445 ± 1.070	2.71 ± 12.69	Treatment	0.951	0.246
305,493,265 ± 8,581,442,302	36,119,272 ± 234,251,825	5,522 ± 1,855	76,631 ± 554,654	1,258.7 ± 9,088.4	-12,280 ± 115,866	9.72 ± 55.40	0.017 ± 0.027	-0.039 ± 0.063	-31.1 ± 110.3	12.35 ± 54.30	-0.020 ± 0.054	0.046 ± 0.125	-2.96 ± 37.33	-23.69 ± 110.01	-0.328 ± 0.514	0.754 ± 1.184	0.93 ± 2.19			
0.326	0.331	0.956	0.595	0.581	0.376	0.308	0.693	0.688	0.159	0.360	0.652	0.651	0.540	0.414	0.196	0.196	0.358			
73,315 ± 92,208	419,643 ± 3,114,460	2,302 ± 1,389	4,100 ± 2,490	58.8 ± 32.3	-158 ± 10,332	1.38 ± 1.30	0.030 ± 0.017	-0.069 ± 0.040	-7.9 ± 52.6	12.88 ± 7.44	-0.022 ± 0.033	0.050 ± 0.077	-6.01 ± 138.42	-40.02 ± 121.76	-0.329 ± 0.641	0.757 ± 1.476	1.11 ± 3.09	Treatment	0.796	0.296
97,295 ± 105,296	105,041 ± 103,907	2,437 ± 1,364	5,004 ± 2,774	72.0 ± 37.0	3,093 ± 4,784	1.50 ± 1.64	0.028 ± 0.016	-0.064 ± 0.037	-14.5 ± 19.5	15.52 ± 17.79	-0.033 ± 0.048	0.076 ± 0.111	10.58 ± 29.73	-34.17 ± 75.48	-0.377 ± 0.516	0.869 ± 1.188	0.54 ± 2.31			
0.251	0.500	0.641	0.105	0.073	0.058	0.695	0.516	0.522	0.431	0.356	0.200	0.201	0.434	0.784	0.691	0.691	0.320			

idered a large effect size. DHA = dehydroAA. Cmax = Maximum observed concentration (from data). Tmax = Time at maximum observed concentration. AUC (0-t) = Cumulative area under curve for experimental time points only: AUC(0-t). AUC∞ (area) = The AUC∞ is the most widely used parameter of the curve area. Trapezoid rule used. AUC∞ (expo) = S (intercept ÷ rate). Based on sum of exponential terms. AUMC∞ (area) = Based on trapezoid calculations. AUMC∞ (expo) = Calculated from exponential terms. MRT (area) = Mean Residence Time (time for 63.2% o nic clearance based on observed data points: AUC(0-t). CL (area) = Based on AUC∞. Operative during elimination phase. CL (area) / kg = Above value divided by body weight in kg. E Phase Intercept = Elimination phase intercept. For oral doses, the sign of the last coefficient (absorption phase) should be negative. E Phase Slope = Elimination Phase Slope. Obtained by linear regression analysis of selected region on graph. E Phase Rate = Elimination Phase Rate. Rate = 2.303 x slope. E Phase Half-life = Time for concentration to diminish by one-half. Remains constant for 1st orde

Table S7a. Low-dose study polymorphonuclear leukocyte (PMN) functionality assessment.

Variable	Treatment	N	Hour 0	Hour 24	Mean (SEM)	Effect	p-Value	η_p^2
Neutrophils	AA	***45***	74.44 ± 29.71	73.19 ± 30.10	73.81 ± 4.08	T	0.222	0.017
[%]	CA	***43***	76.40 ± 32.97	68.84 ± 35.51	72.62 ± 4.17	T x T	0.381	0.009
	Time	***88***	75.40 ± 31.18	71.06 ± 32.74	73.22 ± 2.92			
Without Phagocytosed Bacteria	AA	***45***	77.22 ± 17.50	76.19 ± 16.32	76.71 ± 2.03	T	0.176	0.021
[%]	CA	***43***	71.63 ± 18.28	78.53 ± 15.46 †	75.08 ± 2.07	T x T	0.068	0.038
	Time	***88***	74.49 ± 18.00	77.33 ± 15.86	75.89 ± 1.45			
With Phagocytosed Bacteria	AA	***45***	22.46 ± 17.12	23.40 ± 15.93	22.93 ± 1.98	T	0.169	0.022
[%]	CA	***43***	27.93 ± 17.79	21.15 ± 15.13 †	24.54 ± 2.02	T x T	0.070	0.038
	Time	***88***	25.13 ± 17.57	22.30 ± 15.50	23.74 ± 1.41			

Data are expressed as means ± standard deviations for Treatments A and B. Data were analyzed using a multivariate and univariate General Linear Model with repeated measures. P-levels, with partial ETA squared (η_p^2), are listed for univariate within-subject (Greenhouse-Geisser) time (T), and treatment x time (T x T) effects. Multivariate Wilk's Lambda showed no significant time ($p = 0.210$, $\eta_p^2 = 0.052$) or treatment x time ($p = 0.208$, $\eta_p^2 = 0.052$), within-subject effects. Pairwise comparisons, with LSD confidence interval adjustment, for simple main effects are indicated by the following superscripts: difference from baseline value, † = p<0.05 (‡ = p>0.05 to p<0.10); between treatments, ∗ = p<0.05 (⁑ = >0.05 to p<0.10). η_p^2 effect size values of 0.01 - 0.05 = small, 0.06 - 0.13 = medium, and >0.14 = large.

Table S7b. High-dose study polymorphonuclear leukocyte (PMN) functionality assessment.

Variable	Treatment	N	Hour 0	Hour 24	Mean (SEM)	Effect	p-Value	η_p^2
Neutrophils	AA	***43***	66.51 ± 32.74	68.88 ± 30.04	67.69 ± 3.44	T	0.322	0.011
[%]	CA	***47***	69.16 ± 30.30	75.51 ± 29.53	72.34 ± 3.29	T x T	0.650	0.002
	Time	***90***	67.89 ± 31.34	72.34 ± 29.80	70.01 ± 2.38			
Without Phagocytosed Bacteria	AA	***43***	70.10 ± 14.53	69.42 ± 17.97	69.76 ± 2.12	T	0.419	0.007
[%]	CA	***47***	70.14 ± 14.30	68.44 ± 15.04	69.29 ± 2.03	T x T	0.729	0.001
	Time	***90***	70.12 ± 14.33	68.91 ± 16.42	69.53 ± 1.47			
With Phagocytosed Bacteria	AA	***43***	29.51 ± 14.10	30.10 ± 17.45	29.81 ± 2.05	T	0.413	0.008
[%]	CA	***47***	29.34 ± 13.72	31.08 ± 14.57	30.21 ± 1.96	T x T	0.688	0.002
	Time	***90***	29.42 ± 13.83	30.61 ± 15.93	30.01 ± 1.42			

Data are expressed as means ± standard deviations for Treatments A and B. Data were analyzed using a multivariate and univariate General Linear Model with repeated measures. P-levels, with partial ETA squared (η_p^2), are listed for univariate within-subject (Greenhouse-Geisser) time (T), and treatment x time (T x T) effects. Multivariate Wilk's Lambda showed no significant time ($p = 0.621$, $\eta_p^2 = 0.020$) or treatment x time ($p = 0.414$, $\eta_p^2 = 0.033$), within-subject effects. Pairwise comparisons, with LSD confidence interval adjustment, for simple main effects are indicated by the following superscripts: difference from baseline value, † = p<0.05 (‡ = p>0.05 to p<0.10); between treatments, ∗ = p<0.05 (⁑ = >0.05 to p<0.10). η_p^2 effect size values of 0.01 - 0.05 = small, 0.06 - 0.13 = medium, and >0.14 = large.

Table S8. Peripheral Blood Mononuclear Cells (PBMC) cell differentiation analysis results.

**Antibody** | Cell Population [%]	**reatment**	**N**	**Hour** 0	**Hour** 24	**Mean (SEM)**	**Effect**	**p-Value**	η_p^2
**CD3+** | Total T cells in the blood	AA	***33***	24.17 ± 19.96	30.43 ± 17.79	27.30 ± 2.45	T	0.355	0.013
CD3+ / CD45+	CA	***33***	23.49 ± 20.36	23.63 ± 21.10	23.56 ± 2.45	T x T	0.376	0.012
	Time	***66***	23.83 ± 20.01	27.03 ± 19.66	25.43 ± 1.74			
**CD4+** | T helper cells	AA	***33***	50.44 ± 17.65	34.60 ± 18.43 †	42.52 ± 2.57	T	<0.001	0.239
CD4+ / CD3+	CA	***33***	46.18 ± 17.56	36.33 ± 21.29 †	41.26 ± 2.57	T x T	0.301	0.017
	Time	***66***	48.31 ± 17.60	35.47 ± 19.78 †	41.89 ± 1.82			
**CD4+; CD38+** | Activated T helper cells	AA	***33***	30.25 ± 20.87	30.08 ± 21.96	30.16 ± 2.68	T	0.882	<0.001
CD4+; CD38+ / CD4+	CA	***33***	28.19 ± 21.65	29.48 ± 22.30	28.83 ± 2.68	T x T	0.846	<0.001
	Time	***66***	29.22 ± 21.12	29.78 ± 21.96	29.50 ± 1.90			
**CD4+; IFNg+** | T helper cells expressing IFNg	AA	***33***	3.22 ± 4.80	2.74 ± 4.33	2.98 ± 0.45	T	0.453	0.009
CD4+; IFNg+ / CD4+	CA	***33***	2.38 ± 3.48	1.75 ± 2.73	2.06 ± 0.45	T x T	0.917	<0.001
	Time	***66***	2.80 ± 4.18	2.25 ± 3.63	2.52 ± 0.32			
**CD4+; IL2+** | T helper cells expressing IL2	AA	***33***	15.88 ± 14.49	27.02 ± 25.12 ‡	21.45 ± 2.17	T	0.005	0.114
CD4+; IL2+ / CD4+	CA	***33***	14.27 ± 13.57	26.64 ± 26.47 †	20.46 ± 2.17	T x T	0.881	<0.001
	Time	***66***	15.07 ± 13.95	26.83 ± 25.61 †	20.95 ± 1.54			
**CD4+; TNFa+** | T helper cells expressing TNFa	AA	***33***	25.77 ± 19.31	38.99 ± 25.82 †	32.38 ± 2.72	T	0.015	0.089
CD4+; TNFa+ / CD4+	CA	***33***	25.11 ± 19.28	29.63 ± 19.72	27.37 ± 2.72	T x T	0.223	0.023
	Time	***66***	25.44 ± 19.15	34.31 ± 23.28 †	29.87 ± 1.92			
**Tregs (CD25+ & CD127+)** | Regulatory T cells	AA	***33***	8.10 ± 8.22	12.42 ± 18.45	10.26 ± 1.37	T	0.808	<0.001
Tregs / CD4+	CA	***33***	9.54 ± 12.46	6.33 ± 5.18	7.93 ± 1.37	T x T	0.102	0.041
	Time	***66***	8.82 ± 10.50	9.37 ± 13.80 *	9.10 ± 0.97			
**CD8+** | Cytotoxic T cells	AA	***33***	22.46 ± 11.45	23.11 ± 10.60	22.79 ± 1.43	T	0.171	0.029
CD8+ / CD3+	CA	***33***	26.46 ± 13.48	20.34 ± 10.07 †	23.40 ± 1.43	T x T	0.091	0.044
	Time	***66***	24.46 ± 12.57	21.73 ± 10.35	23.10 ± 1.01			
**CD8+; CD38+** | Activated Cytotoxic T cells	AA	***33***	12.49 ± 9.58	37.35 ± 28.48 †	24.92 ± 2.38	T	<0.001	0.314
CD8+; CD38+ / CD8+	CA	***33***	15.28 ± 12.81	34.15 ± 27.56 †	24.71 ± 2.38	T x T	0.461	0.009
	Time	***66***	13.88 ± 11.31	35.75 ± 27.85 †	24.82 ± 1.68			
**CD8+; IFNg+** | Cytotoxic T cells expressing IFNg	AA	***33***	1.20 ± 1.79	9.59 ± 11.40 †	5.39 ± 1.12	T	<0.001	0.272
CD8+; IFNg+ / CD8+	CA	***33***	1.36 ± 2.07	8.57 ± 14.03 †	4.97 ± 1.12	T x T	0.713	0.002
	Time	***66***	1.28 ± 1.92	9.08 ± 12.69 †	5.18 ± 0.79			
**CD8+; IL2+** | Cytotoxic T cells expressing IL2	AA	***33***	19.91 ± 16.68	23.75 ± 20.34	21.83 ± 2.18	T	0.104	0.041
CD8+; IL2+ / CD8+	CA	***33***	18.46 ± 17.96	25.28 ± 17.36	21.87 ± 2.18	T x T	0.646	0.003
	Time	***66***	19.18 ± 17.21	24.51 ± 18.78	21.85 ± 1.54			
**CD8+; TNFa+** | Cytotoxic T cells expressing TNFa	AA	***33***	20.71 ± 17.49	42.76 ± 21.80 †	31.74 ± 2.37	T	<0.001	0.388
CD8+; TNFa+ / CD8+	CA	***33***	19.59 ± 17.07	40.97 ± 20.94 †	30.28 ± 2.37	T x T	0.922	<0.001
	Time	***66***	20.15 ± 17.16	41.87 ± 21.23 †	31.01 ± 1.68			
**NK cells (CD16+ & CD56+)** | Natural Killer Cells	AA	***33***	13.78 ± 10.11	20.26 ± 15.56	17.02 ± 2.03	T	0.968	<0.001
NK cells / CD3-	CA	***33***	25.98 ± 25.21	19.78 ± 17.19	22.88 ± 2.03	T x T	0.062	0.054
	Time	***66***	19.88 ± 20.03 *	20.02 ± 16.27	19.95 ± 1.43 *			
**NKG2D+** | NK Cells expressing NKG2D	AA	***33***	39.08 ± 30.65	31.46 ± 26.57	35.27 ± 3.27	T	0.100	0.042
NKG2D+ / NK cells	CA	***33***	37.86 ± 32.49	26.25 ± 30.01	32.05 ± 3.27	T x T	0.731	0.002
	Total	***66***	38.47 ± 31.35	28.85 ± 28.25	33.66 ± 2.31			

Data are expressed as means ± standard deviations for Treatments A and B. Data were analyzed using a multivariate and univariate General Linear Model with repeated measures. P-levels, with partial ETA squared (η_p^2), are listed for univariate within-subject (Greenhouse-Geisser) time (T), and treatment x time (T x T) effects. Multivariate Wilk's Lambda showed significant time ($p<0.001$, $\eta_p^2 = 0.707$), but not treatment x time ($p = 0.457$, $\eta_p^2 = 0.217$), within-subject effects. Pairwise comparisons, with LSD confidence interval adjustment, for simple main effects are indicated by the following superscripts: difference from baseline value, † = p<0.05 (‡ = p>0.05 to p<0.10); between treatments, ∗ = p<0.05 (⁑ = >0.05 to p<0.10). η_p^2 effect size values of 0.01 - 0.05 = small, 0.06 - 0.13 = medium, and >0.14 = large.
